# A 2nd-generation scalable synthesis of the HIV-1 entry inhibitor CJF-III-288 enabled by photoredox catalysis

**DOI:** 10.1039/d5cc06802a

**Published:** 2026-01-05

**Authors:** Jonathan W. Nadraws, Maithili S. Pokle, Amos B. Smith

**Affiliations:** a Department of Chemistry, University of Pennsylvania 231 South 34th St Philadelphia Pennsylvania 19104 USA jnadraws@sas.upenn.edu; b Maithili S. Pokle – Department of Chemistry, University of Pennsylvania 231 South 34th St Philadelphia Pennsylvania 19104 USA mapokle@sas.upenn.edu; c Department of Chemistry, University of Pennsylvania 231 South 34th St Philadelphia Pennsylvania 19104 USA smithab@sas.upenn.edu

## Abstract

The development and optimization of a 2nd-Generation process synthesis for the human immunodeficiency virus-1 entry inhibitor CJF-III-288 bis-trifloroacetate salt 1 is reported. The route eliminates the use of noble metals, decreases the step count nearly by half, and increases the overall yield tenfold.

## Abstract 2

The development of a 2nd-generation synthesis for the human immunodeficiency virus-1 entry inhibitor CJF-III-288 bis-trifloroacetate salt 1 is reported.

## Graphical abstract

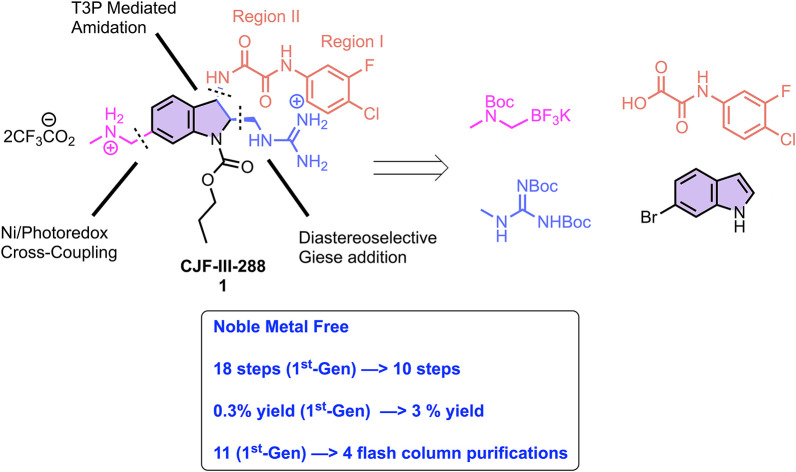

As of 2023, 39.9 million people worldwide were living with the human immunodeficiency virus (HIV), with 1.3 million new infections that year alone.^1^ HIV infection without treatment leads to acquired immunodeficiency syndrome (AIDS), a range of conditions that weaken the immune system thereby increasing the risk of acquiring other common and/or opportunistic infections, greatly reducing life-expectancy.^2^ The highly active antiretroviral therapy (HAART) regimen results in almost total restoration of normal immune function and a normal lifespan, but does not eliminate viral reservoirs, necessitating lifelong adherence to treatment.^3^ Thus, in order to end the HIV epidemic, curative treatment is needed.

Targeting the viral envelope glycoprotein (Env), the only virus-specific protein on the surface of virus particles and infected cells, comprises a selective tactic to block the viral entry process and target the viral reservoir.^4^ CD4-mimetic compounds (CD4mcs) developed in the Smith Group bind to Env and expose gp120 epitopes recognized by otherwise “non-neutralizing” antibodies, blocking viral entry and allowing the neutralization of infectious HIV-1 virus particles. Our lead CD4mc CJF-III-288 displays promising low micromolar activity against many primary strains of HIV-1 and enhances binding of HIV+ plasma to HIV infected cells and increases HIV+ plasma mediated antibody-dependent cellular cytotoxicity (ADCC) compared to previous generations of CD4mc.^4^ These results justified the development of a more efficient synthesis of CJF-III-288 to access larger quantities of the compound to facilitate animal studies. The established route to synthesize CJF-III-288, developed initially to enable structure–activity relationship (SAR) studies of the indoline CD4mcs, is a laborious and time-consuming 18 steps long, with 11 flash column purifications and an overall yield of 0.3% (Fig. 1).^4^ The use of noble and toxic metals, such as osmium, palladium, and tin, is also undesirable from a cost and safety standpoint, and triphosgene's use poses a significant safety hazard in the lab when used on scale. The length derives from protecting group and functional group manipulations, and the reliance on two-electron transformations for installing the amine functionality. The need to build up negative charge at the transmetalating carbon of the alpha-amino coupling partners may lead to poor reactivity; the same is true for the corresponding Michael addition donors.^5^ Additionally, these reactions often require harsh nucleophiles and bases that pose issues for functional group sensitivity. Alternatively, alpha-amino radicals have seen extensive use in photoredox catalysis as their high stability and nucleophilicity makes them useful reagents in a variety of reactions.^6^ Radical chemistry is also generally more functional group tolerant and avoids many of the substrate sensitivity issues seen in two electron chemistry.

**Fig. 1:**
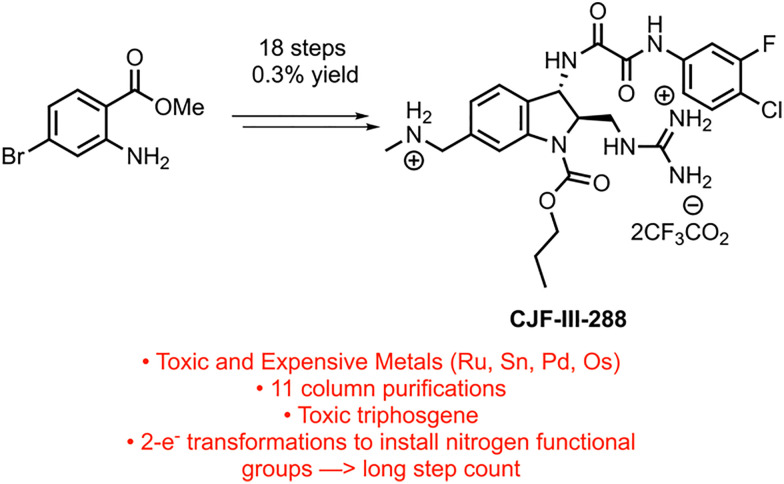
1st-generation synthesis.

The methylamino methyl side chain and methyl guanidine substituent were identified as areas where the implementation of photoredox chemistry would lead to significant improvements in the efficiency of the route. The 2nd-generation retrosynthesis of CJF-III-288 begins with late-stage introduction of the aryl oxalamide moiety *via* amidation of amine 2, which in turn could be prepared *via* Curtius rearrangement from carboxylic acid 3 (Scheme 1). Installation of the methylamino methyl side chain was envisioned using Nickel/photoredox dual catalysis with aryl bromide 4 as the electrophile. Diastereoselective Giese addition into Michael acceptor 5 with judicious choice of chiral auxiliary (X) was proposed to set the stereogenicity of the 2 and 3 positions of the indoline core and install the guanidine motif in one step. Early stage carbamylation would obviate the need to swap out the carbamate group at a later stage, as in the 1st-generation synthesis. Finally, an early stage Vilsmeier–Haack formylation would be implemented to install the carbonyl group at the 3 position of the indoline core, beginning from commercially available 6-bromoindole 6.

**Scheme 1:**
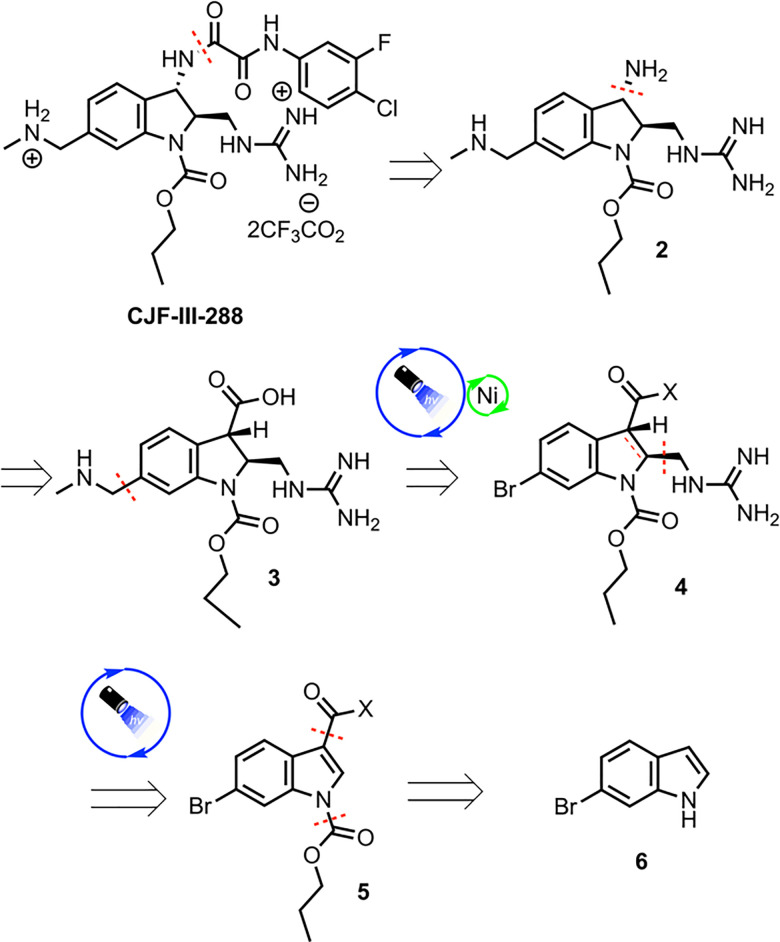
Retrosynthesis of CJF-III-288.

Investigation of a 2nd-generation synthesis of CJF-III-288 begins with Vilsmeier–Haack formylation of 6 (Scheme 2), which proceeds in near quantitative yield on 105-gram scale.^7^ An earlier attempt to convert 6 directly to the carboxylic acid was unsuccessful, as the resulting acid lacked enough solubility in common organic solvents to make it useful for further chemistry. Carbamylation of 7 with propyl chloroformate then furnishes propyl carbamate 8, again in excellent yield on nearly an 80-gram scale. A Pinnick oxidation of aldehyde 8 next leads to carboxylic acid 9 in an excellent 87% yield on a 91-gram scale.^8^ These first three steps proceed without silica-gel chromatography or recrystallization in an overall 84% yield. Acid 9 serves as the coupling partner with the commercially available, inexpensive (+)-camphorsultam 10 to yield 11 in 78% yield after silica-gel chromatography. The resulting amide 11 was envisioned as a Giese acceptor in a photoredox Giese addition using a guanidine-based radical donor.

**Scheme 2:**
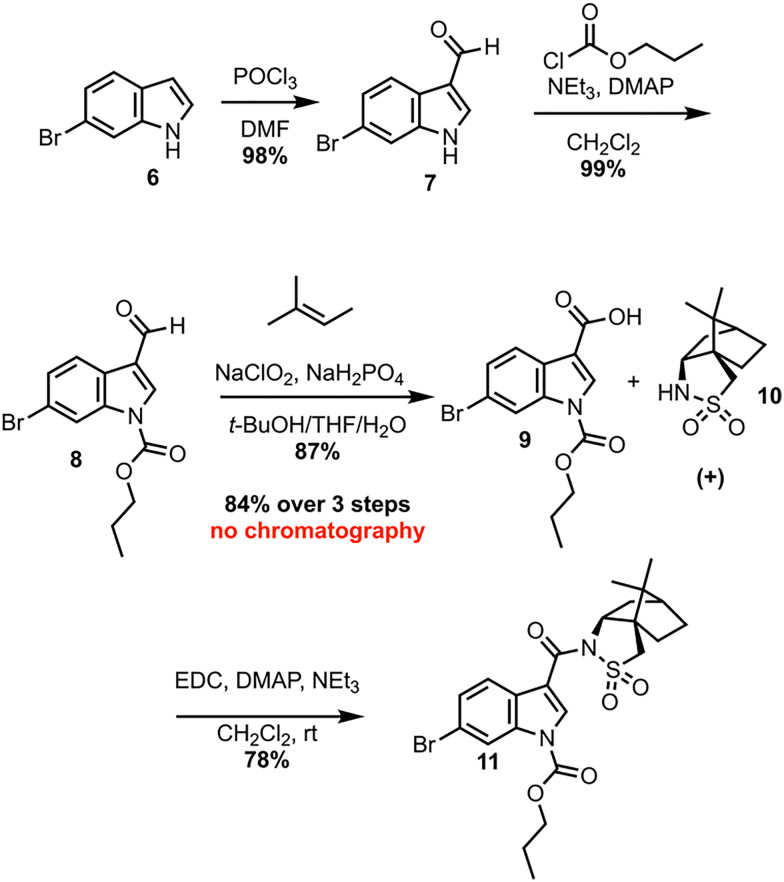
Early-stage synthesis of CJF-III-288.

The dearomatization of indoles bearing a champhorsultam moiety as a chiral auxiliary is established chemistry, with high diastereoselectivities observed in previous investigations.^9^ Hydrogen atom transfer (HAT) catalysis was selected for its good atom economy, operational simplicity, and the ease by which the C–H radical precursors can be accessed (Scheme 3). Investigations began with globally protected precursor 12 to minimize potentially reactive guanidine nitrogen sites. HAT catalysts are chemical species that abstract homolytically labile hydrogen atoms from molecules. Under numerous protocols employing HAT catalysts and catalytic systems, such as Eosin Y, the “push–pull” benzophenone catalyst 4-Methoxy-4′-trifluoromethylbenzophenone and copper cocatalyst, and a dual catalytic system employing iridium and quinuclidine catalysts, no formation of the adduct 13 was observed.^10–12^ A prohibitively large C–H bond dissociation energy (BDE) of 12, or diminished nucleophilicity (due to either enhanced stability or steric hindrance) of the corresponding radical, was hypothesized as potential reasons for the lack of reactivity. Consequently, precursor 14, with no *tert*-butoxycarbonyl (Boc) group at the nitrogen alpha to the reactive center, was employed for further investigations. Utilizing tetrabutylammonium decatungstate (TBADT) as the HAT catalyst, the desired Giese adduct 15 was observed by liquid chromatography-mass spectrometry (LC-MS) analysis, in low conversion. After additional optimization, the Giese adduct 15 was isolated in a good yield of 59% with 10.4 : 1 *trans* : *cis* diastereoselectivity, and the absolute stereochemistry was confirmed by X-ray crystallography (see SI). The reaction can be performed in up to 60.3 mmol scale in batch, and the TBADT catalyst can be synthesized in house on multi-gram scale for less than a dollar per gram. Discussion of the optimization of the Giese addition may be found in the SI.

**Scheme 3:**
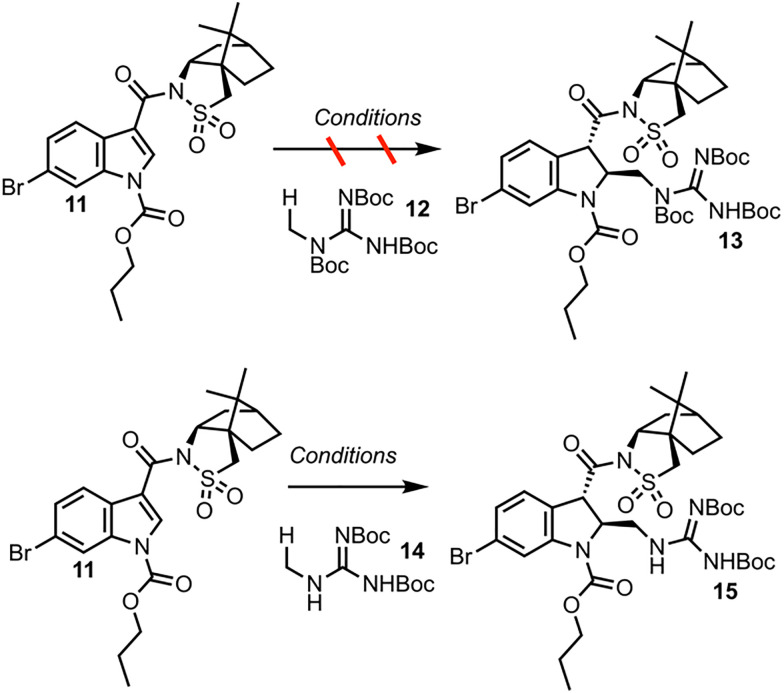
Investigation of the Giese addition.

Numerous protocols exist for the dual photoredox/Ni catalyzed cross coupling of alpha-amino bearing coupling partners and aryl halides. The protocols first investigated were those employing the most easily synthetically accessible alpha-amino radical precursors (Scheme 4). With 15 as the aryl halide coupling partner, Dual Ir/Ni catalysis with Boc sarcosine 16 afforded only decomposition of the starting materials and none of the desired product 17.^13^ A HAT protocol employing 4-methoxy-4′-trifluoromethylbenzophenone and Boc dimethylamine 18 afforded the desired cross-coupling adduct 17 as observed by LC-MS analysis, albeit in low conversion, likely due to the poor photo physics of the benzophenone triplet state sensitizers.^14^ To remedy this, protocols and radical precursors that would lead to more efficient generation of the desired alpha amino radical were sought out. Employing redox-active ester 19 in a net-reductive cross coupling protocol with stoichiometric photoreductant Hantzsch ester affords 17 in 13% yield on 2 mmol scale.^15^ While an appreciable amount of the desired product was obtained, the yield was low due to a largely inefficient reaction producing significant protodehalogenation side product and leaving a significant amount of unreacted starting material. The net-reductive reaction conditions and rapid fragmentation of 19 likely leads to fast generation of the corresponding alpha amino radical, but in the absence of an appreciable amount of oxidative addition complex, non-productive processes such as radical quenching and ultimately protodehalogenation of 15 predominate. The solution was to employ a net-neutral process where the build-up of reactive intermediates could be minimized. Thus, employing CzIPN as the organic photocatalyst and (2,2′-bipyridine)nickel dichloride (NiCl_2_(bpy)), along with trifluoroborate precursor 20, the desired adduct 17 was obtained in 75% yield after flash column chromatography on 7.6 mmol scale.^16^ Discussion of the optimization of the cross-coupling may be found in the SI.

**Scheme 4:**
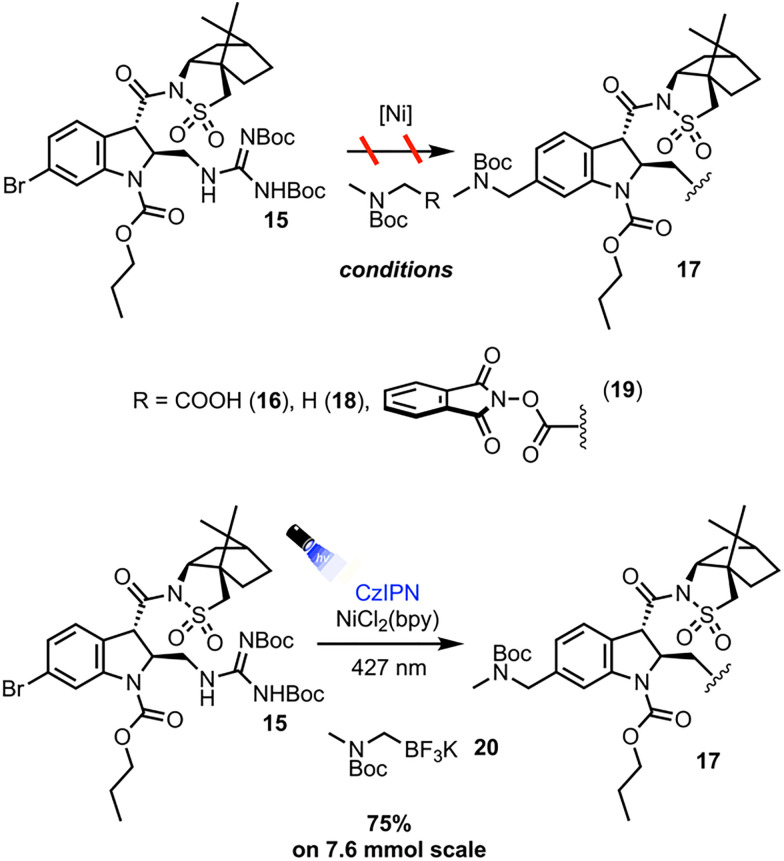
Investigation of the cross-coupling.

The final synthetic challenge left to be overcome in the 2nd-generation synthesis of CJF-III-288 was the conversion of the carbonyl group at position 3 of the indoline to the primary amine 21 (Scheme 5). Applying Curtius conditions to 17 with treatment with diphenyl phosphoryl azide (DPPA) under refluxing conditions, instead of resulting in intermolecular capture of the resulting phosphate ester with azide to afford acyl azide, results in intramolecular capture by the guanidine moiety to give 22. The solution was to employ a reaction mechanistically analogous to the Curtius rearrangement but proceeding through less electrophilic intermediates, the lesser known Lössen rearrangement.^17^ Aminolysis of 17 with hydroxylamine furnishes hydroxamic acid 23. Compound 23 is used in a self-propagative, “pseudo-catalytic” Lössen rearrangement, wherein condensation with acetonitrile first occurs to activate the substrate *in situ*, which undergoes rearrangement to isocyanate 24. Compound 24 is either hydrolyzed, or enters the self-propagative catalytic cycle with another equivalent of 23 to afford primary amine 21. Data detailing the optimization of these two transformations is found in the SI.

**Scheme 5:**
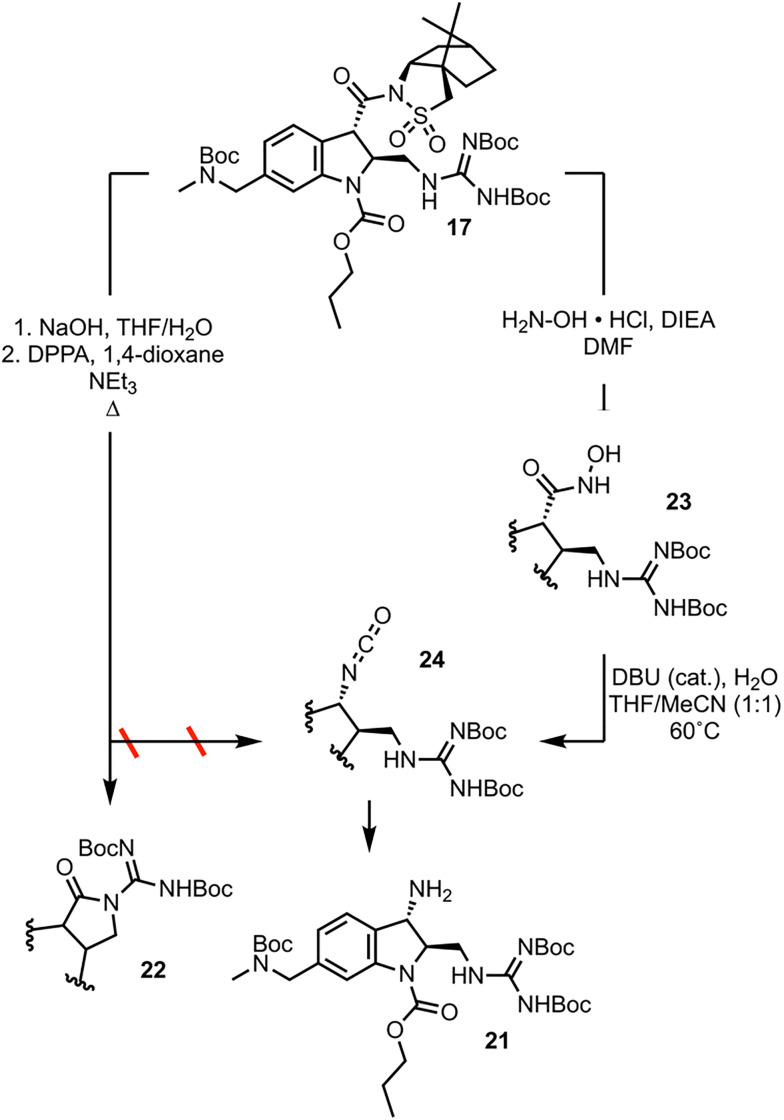
Development of the late-stage synthesis.

The synthesis was completed by coupling of the *meta*-fluoro-*para*-chloro acid 25 to amine 21 using propanephosphonic acid anhydride (T3P), effective for sterically encumbered substrates on large scale (Scheme 6).^18,19^ Global Boc deprotection of 26 with TFA yields CJF-III-288 in nearly quantitative yield. The synthesis was further validated by scaling up the cross coupling of 15 and 20, which can be performed on a 33.16 mmol scale in a single batch. The sequence from intermediate 15 to 26 was performed on a 79 mmol scale, affording 5.66 grams of 26 after a single flash column purification. The global Boc deprotection yielded 4.90 grams of 1.

**Scheme 6:**
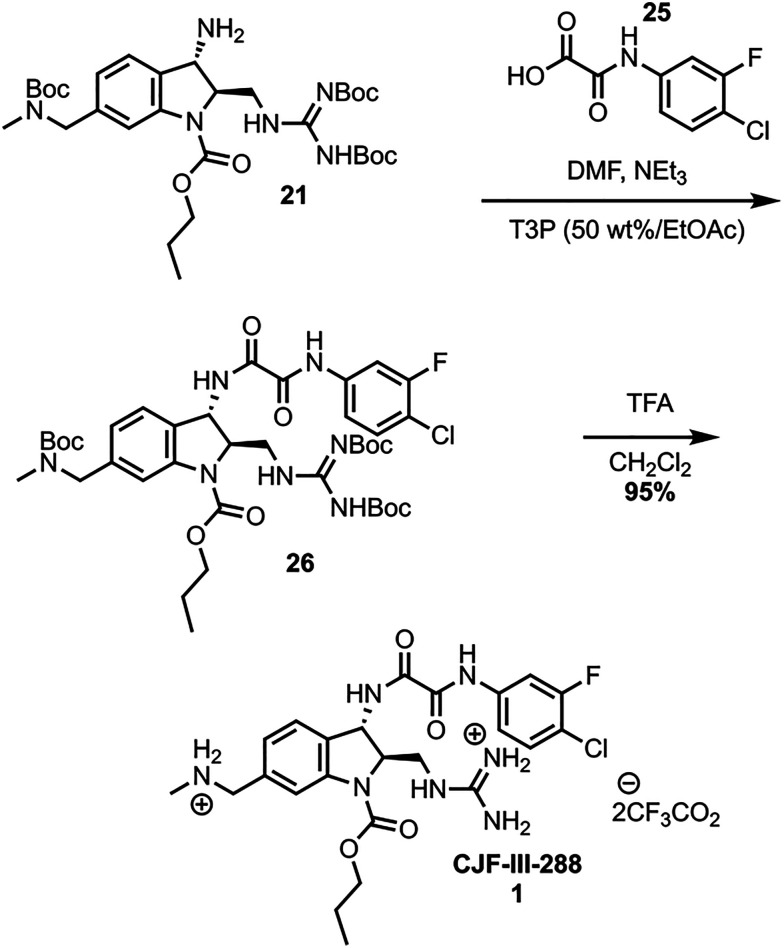
Endgame synthesis.

In summary, the 2nd-generation synthesis of CJF-III-288, beginning on over a hundred-gram scale, proceeds in 10 steps to yield 1 in an overall yield of 3%. This reduces the step count almost by half compared to the original of 18 steps, and the overall yield has increased tenfold compared to the original yield of 0.3%. This was achieved by reducing the step count of the installation of the guanidine group and stereochemistry from 5 steps to 1 step, and of the methylamino methyl group from 3 steps to 1 step, compared to the first-generation synthesis. The route has been used to synthesize 5.7 grams of 1, which are currently being used in *in vivo* biological studies to determine the efficacy of CJF-III-288 to prevent and cure HIV infection in monkey models. Additionally, the photochemistry developed for the synthesis holds promise for use in further analogue synthesis. The modular nature of both reactions allows incorporation of diverse alkyl, aryl, methylamino, and methyloxy functionality at both the 2 and 6 positions of the indoline core and further optimization of the potency and physicochemical properties of the indole CD4mcs.

## Conflicts of interest

There are no conflicts to declare.

## Supplementary Material

CC-062-D5CC06802A-s001

CC-062-D5CC06802A-s002

## Data Availability

The data underlying this study, including experimental procedures, characterization data, ^1^H-NMR/^13^C-NMR spectra, and crystal structures are available in the supplementary information (SI). Supplementary information is available. See DOI: https://doi.org/10.1039/d5cc06802a. Raw FID data is openly available in Dryad Databank at DOI: https://doi.org/10.5061/dryad.4b8gthtsf. CCDC 2484437 contains the supplementary crystallographic data for this paper.^20^
