# Supplementary material for: A 2nd-generation scalable synthesis of the HIV-1 entry inhibitor CJF-III-288 enabled by photoredox catalysis
Source: Chem Commun (Camb). 2026 Jan 5;62(9):2981–5. doi: 10.1039/d5cc06802a (PMC12794802; doi:10.1039/d5cc06802a)
Supplement: CC-062-D5CC06802A-s001 [file CC-062-D5CC06802A-s001.pdf]

Supplementary Information (SI) for Chemical Communications.
This journal is © The Royal Society of Chemistry 2026

Supporting Information

A 2nd-Generation Scalable Synthesis of the HIV-1 Entry Inhibitor CJF-III-288 Enabled by Photoredox Catalysis

Jonathan W. Nadraws*, Maithili S. Pokle, and Amos B. Smith III

Department of Chemistry, University of Pennsylvania, Philadelphia, PA 19104, U.S.A.

jnadraws@sas.upenn.edu

1. General Details……………………………………………………………………2

2. Experimental……………………………………………………………………5

3. Additional Discussion of the Optimization of the Giese Addition……………………25

4. Additional Discussion of the Optimization of the Photoredox Cross-Coupling…………...29

5. ^{1}H-NMR and ^{13}C-NMR spectra……………………………………………35

6. X-Ray Structure and Determination of Compound 15……………………………..66

7. References……………………………………………………………………...82

1. General Details

All reactions were conducted in flame-dried glassware under an inert atmosphere of argon, unless otherwise stated. All solvents were reagent or high-performance liquid chromatography (HPLC) grade. Anhydrous CH_2Cl_2 and NEt_3 were obtained from a calcium hydride still. Anhydrous THF was obtained from a Pure SolveTM PS-400 system under an argon atmosphere. DMF, MeCN, and DIEA were dried over 4 Å molecular sieves. All reagents were purchased from commercially available sources and used as received. Reactions monitored by thin layer chromatography (TLC) were performed on pre-coated silica gel 60 F-254 plates (40-55 micron, 230-400 mesh) and visualized by UV light or staining with potassium permanganate (potassium permanganate, potassium carbonate, sodium hydroxide) and heating. Yields refer to chromatographically and spectroscopically pure compounds. Proton (^{1}H) and carbon (^{13}C) NMR spectra were recorded on a Bruker Avance III 500-MHz spectrometer, a Bruker DRX500 500-MHz spectrometer, a Bruker NEO600 600-MHz spectrometer, or a Bruker NEO400 400-MHz spectrometer. Chemical shifts (δ) are reported in parts per million (ppm) relative to methanol (δ 3.31), acetone (δ 2.05), chloroform (δ 7.26), and dimethyl sulfoxide (δ 2.50) for ^{1}H NMR, and methanol (δ 49.15), acetone (δ 29.8), chloroform (δ77.16), and dimethyl sulfoxide (δ39.52) for ^{13}C NMR. High resolution mass spectra (HRMS) were recorded at the University of Pennsylvania Mass Spectroscopy Service Center on either a VG Micromass 70/70H or VG ZAB-E spectrometer. UPLC-MS analysis was performed on a Waters Acquity ultra performance liquid chromatography (UPLC) instrument (Milford, MA) equipped with a binary gradient pump and a Tunable UV (TUV) UV detector. A Waters (Milford, MA) Acquity UPLC HSS C18 column (2.1 x 50 mm, 1.8 μm particle size) along with a prefilter was used with the column temperature maintained at 30 °C. A binary eluent system was employed with mobile phase A (water with 0.1% formic acid) and mobile phase B (acetonitrile with 0.1% formic acid) with a flow rate of 0.5 mL/min and a gradient program as follows: 0.00-0.50 min, 5% B; 0.50-2.50 min, linear from 5% to 95% B; 2.50-3.00 min, 95% B, 3.00-3.50 min, 5% B. For preparation of the eluents, Optima™ LC/MS- grade

water, acetonitrile and formic acid were purchased from Fisher Scientific and used without further purification. The sample injection volume was 2 µL. The TUV detector was set to 254 nm. The UPLC instrument was coupled to a Waters SQD sinle quadrupole mass spectrometer (Milford, MA) with a Zspray electrospray ionization source using nitrogen as desolvation and nebulization gas. Source conditions were as follows: capillary voltage, 3.00 kV; cone voltage, 35 V; desolvation temperature, 350 °C; desolvation gas flow rate, 500 L/h; cone gas flow rate 0 L/h. The mass spectrometer was operated in both positive and negative mode scanning m/z 200-2000. Preparative scale HPLC was performed with a Gilson 333/334 preparative pump system equipped with a 5 mL injection loop, Sunfire C18 OBD column (5 µm packing material, 19 x 100 mm column dimensions) equipped with a UV-Vis dual wavelength (210 and 254 nm) detector and 215 liquid handling module. Solvent systems were comprised of H_2O containing 0.1% v/v trifluoroacetic acid, and acetonitrile containing 0.1% v/v trifluoroacetic acid. Lyophilization was performed in a Labconco FreeZone 12 Plus lyophilizer (0.148 mbar). The purity of new compounds was judged by NMR and LCMS (>95%).

Photoreactions were performed in batch in 40-mL clear-glass vials from Chemglass (CG-4909-05) with 0.125" polyprolyene/PTFE/silicone caps from Chemglass (CG-4911-20) using either 427 nm PR160L Kessil lamps (KSPR160L-427) or 370 nm PR160L Generation 2 Kessil lamps (PR160L-370-G2) at 100% power with cooling by SUNON fans (SP101A-1123HST.GN). Representative photos of the reaction setup are shown below.

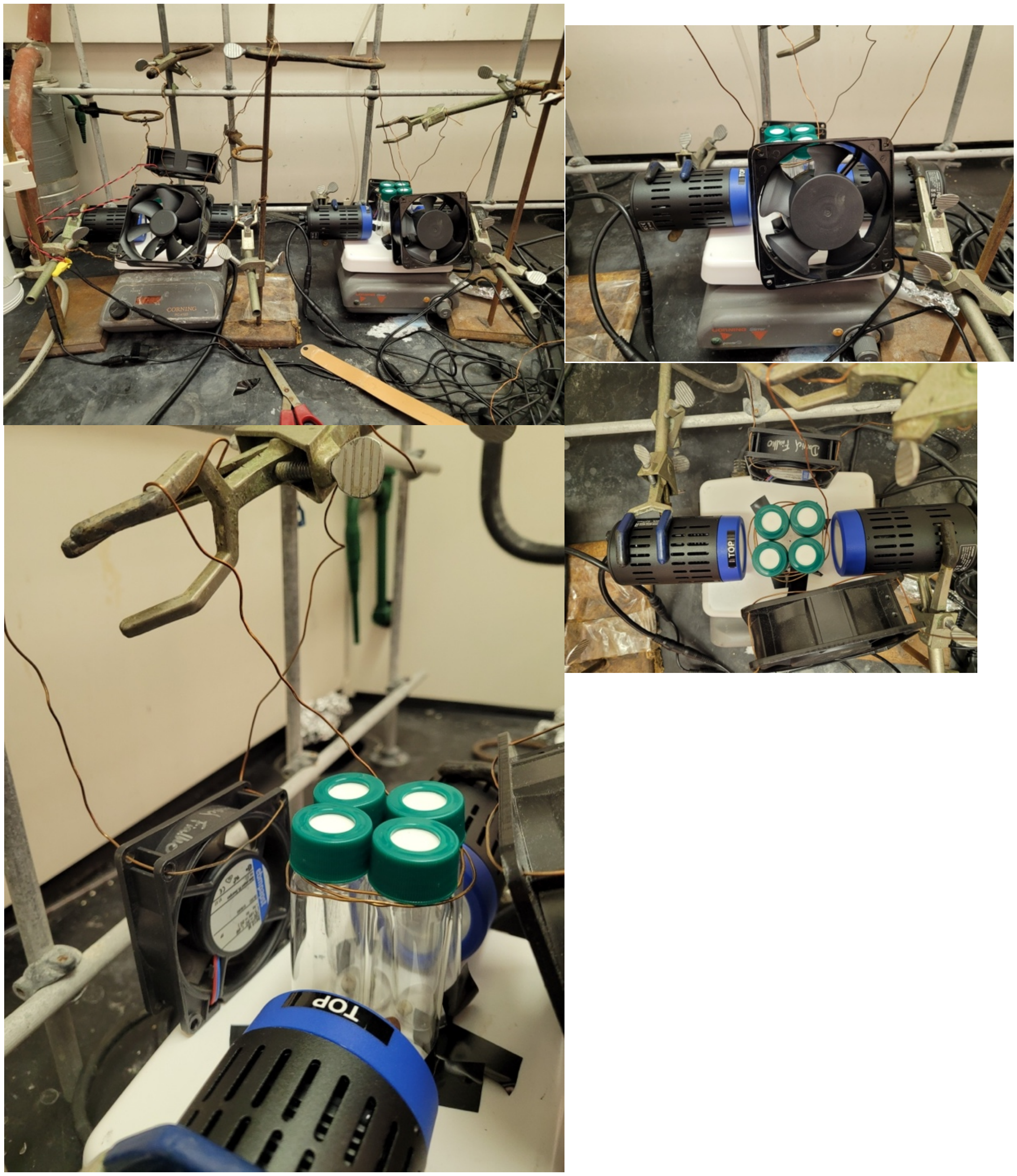

Figure S-1. Photochemical Reaction Setup. From the top left, clockwise: I. Photoreaction setup of 8 vials. II. Reaction setup top view. III. Reaction setup front view. IV. Reaction setup side view.

2. Experimental

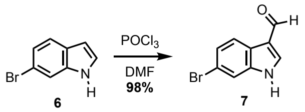

Compound 7

A flame-dried 5 L round bottom flask equipped with magnetic stirring was charged with 670 mL dry DMF, and the flask was cooled to 0 ˚C in an ice/water bath. $POCl_3$ (112 mL, 1210 mmol, 2.25 equiv) was added as a gentle stream at 0 ˚C. After stirring for 15 min, a solution of **6** (105.0 g, 535.6 mmol) in dry DMF (558 mL) was added as a stream via cannula. The ice bath was removed, and the reaction mixture was stirred at ambient temperature for one h. After, the reaction mixture was slowly quenched with 297 g of KOH in 1117 mL deionized H_2O (dH_2O), slowly enough to keep the mixture just below boiling. Approximately 1/6th of the quench solution was used, and the reaction mixture was stirred in open air overnight. The following day, the quench was completed, and the reaction mixture allowed to cool to room temperature. The reaction mixture was poured into 1180 mL sat. aq $NaHCO_3$ + 1180 mL EtOAc and allowed to sit overnight before workup. The two phases were separated, and the aqueous phase extracted with 2L EtOAc, the combined organic phases washed with sat. aq Brine (2 x 2L), 5% (w/v) aq LiCl, then dried over $MgSO_4$, gravity filtered & concentrated *in vacuo* to yield an amorphous light-brown solid. Yield = 117.9 g (98%). Spectral data matches that previously reported data for **7**.[1]

^{1}H NMR (500 MHz, DMSO-d$_6$) δ9.93 (s, 1H), 8.31 (s, 1H), 8.02 (d, *J* = 8.4 Hz, 1H), 7.71 (d, *J* = 1.6 Hz, 1H), 7.36 (dd, *J* = 8.5, 1.8, 1H);

^{13}C NMR (125 MHz, DMSO-d$_6$) δ185.2, 139.2, 138.0, 125.1, 123.2, 122.5, 118.0, 115.96, 115.2;

HRMS (ESI) *m/z*: $[M+H]^+$ Cald for C_9H_6BrNO 223.9706; Found 223.9711.

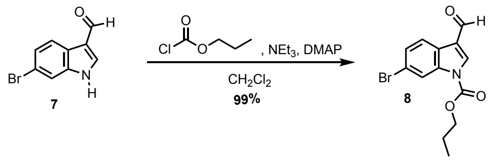

Compound 8

A flame-dried 3 L round bottom flask equipped with magnetic stirring was charged with **7** (79.8 g, 356 mmol, 1 equiv) and DMAP (435.2 mg, 35.62 mmol, 0.1 equiv), then dry CH_2Cl_2 (937 mL) was added via a flame-dried addition funnel. The flask was purged with Ar, then NEt_3 (52.2 mL, 374 mmol, 1.05 equiv) and propyl chloroformate (41.9 mL, 374 mmol, 1.05 equiv) were added sequentially, with a vent needle in the flask during chloroformate addition. At 2.5 h, after confirming the reaction was complete by LC-MS analysis, the reaction mixture was poured into 4500 mL 1:1 CH_2Cl_2 – sat. a. $NaHCO_3$, and the organics washed with $NaHCO_3$. The $NaHCO_3$ layer was extracted with CH_2Cl_2 (2 x 550 mL), and the combined organics were washed with aqueous 1 M HCl (1800 mL), then the HCl layer was extracted with CH_2Cl_2 (2 x 550 mL). The combined organics were dried over $MgSO_4$, gravity filtered and concentrated *in vacuo* to yield an amorphous peach colored solid. Yield = 109.58 g (99%).

*Note: During chloroformate addition the reaction mixture visibly refluxed and was purged with Ar until refluxing ceased.

^{1}H NMR (500 MHz, $CDCl_3$) δ10.01 (s, 1H), 8.26 (s, 1H), 8.14 (s, 1H), 8.06 (d, J = 8.4 Hz, 1H), 7.42 (dd, J = 8.4, 1.6 Hz, 1H), 4.44 (t, J = 6.7 Hz, 2H), 1.88 (sx, J = 7.2 Hz, 2H), 1.08 (t, J = 7.5 Hz, 3H);

^{13}C NMR (125 MHz, $CDCl_3$) δ185.3, 149.8, 136.3, 136.0, 128.0, 124.7, 123.2, 121.6. 112.0, 118.2, 70.1, 22.0, 10.3;

HRMS (ESI) *m/z*: $[M+H]^+$ Cald for $C_{13}H_{12}BrNO_3$, 309.0001; Found 308.9998.

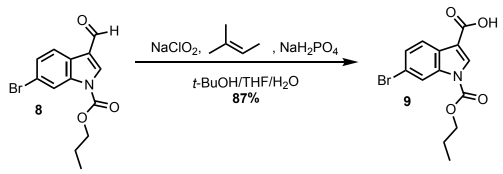

Compound 9

An air-dried 8 L round bottom flask equipped with mechanical stirring was charged with **8** (91.2 g, 294 mmol, 1 equiv), then $NaH_2PO_4 \cdot 2\ H_2O$ (137.6 g, 882.3 mmol, 3 equiv). then *t*-BuOH (1610 mL), all in open air. After, 2-methylbut-2-ene solution (2 M in THF, 294 mL, 588 mmol, 2 equiv), was added in open air, and the reaction mixture was stirred briefly, followed by addition of $NaClO_2$ (79.8 g, 882 mmol, 3 equiv) in 138 mL dH_2O poured from a glass beaker/flask. The reaction mixture was stirred overnight. After, the reaction mixture was poured into a 6L separatory funnel with 770 mL dH_2O + 770 mL EtOAc., and the mixture shaken. The aqueous and organic phases were separated, and the aqueous phase acidified with concentrated HCl to pH ~2-3. The aqueous phase was extracted with EtOAc (3 x 450 mL). After, the combined organics were washed with sat. aq brine (890 mL), dried over $MgSO_4$, gravity filtered and concentrated *in vacuo*. The crude material was suspended in 740 mL EtOAc and washed with 533 mL sat. aq $NaHCO_3$, resulting in the precipitation of a of a white solid. The entire suspension was filtered on a Büchner funnel. The solid was washed with EtOAc until the filtrate ran clear. The collected filtrate, which still had undissolved solid, was filtered on another Büchner funnel, and the solid was washed with EtOAc until the filtrate ran clear. This process was repeated until no more solid was present in the filtrate, for a total of six filtrations. The solid was transferred to a 2 L separatory funnel, suspended in 1200 mL EtOAc, and washed with 1 M HCl (300 mL, then 200 mL). The aqueous and organic phases were separated, and the organics diluted to 2750 mL EtOAc, which finally brought all solid into solution. The organics were dried over $MgSO_4$, gravity-filtered, and concentrated *in vacuo* to yield a white powder. Yield = 83.04 g (87%)

^{1}H NMR (500 MHz, $CDCl_3$) δ8.34 (s, 1H), 8.29 (s, 1H), 8.00 (d, J = 8.4 Hz, 1H), 7.46 (dd, J = 8.4 Hz, 1.7 Hz, 1H), 4.45 (t, J = 6.7 Hz, 2H), 1.90 (sx, J = 7.2 Hz, 2H), 1.09 (t, J = 7.4 Hz, 3H);

^{13}C NMR (125 MHz, $CDCl_3$) δ169.2, 150.0, 136.2, 133.3, 127.8, 126.2, 122.9, 119.4, 118.4, 112.1, 70.1, 22.0, 10.4;

HRMS (ESI) *m/z*: $[M+H]^-$ Cald for $C_{13}H_{11}BrNO_4$, 323.987694; Found 323.987696.

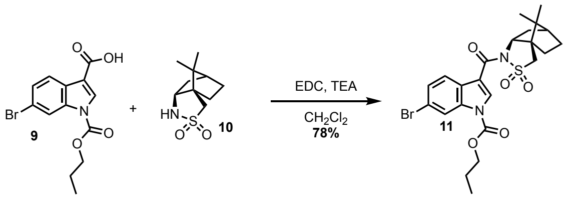

Compound 11

A flame-dried 3 L round bottom flask equipped with magnetic stirring was charged with **9** (57.540 g, 176.42 mmol, 1 equiv), **10** (37.986 g, 176.42 mmol., 1 equiv), EDC (40.587 g, 211.71 mmol, 1.2 equiv), and DMAP (4.311 g, 35.28 mmol, 0.2 equiv) in open air. Freshly distilled, dry CH_2Cl_2 (882 mL), then freshly distilled, dry TEA (49.2 mL, 353 mmol, 2 equiv) were added, the reaction mixture purged with Ar and stirred overnight. After, the reaction mixture was diluted with 230 mL CH_2Cl_2, washed with 830 mL 1 M HCl, then 1 L sat. aq $NaHCO_3$, then the reaction mixture was dried over $MgSO_4$, gravity filtered and concentrated *in vacuo*. The crude material was loaded onto 400g of celite and purified by flash column chromatography (15% EtOAc/Hexanes, $R_f = 0.26$) to yield a white, foamy solid. Yield = 71.66 g (78%).

^{1}H NMR (500 MHz, $CDCl_3$) δ8.52 (s, 1H), 8.34 (s, 1H), 7.93 (d, *J* = 8.5 Hz, 1H), 7.46 (dd, *J* = 8.5, 1.51 Hz, 1H), 4.44 (m, 2H), 4.27 (dd, *J* = 7.9, 4.7 Hz, 1H), 3.58 (d, *J* = 13.7 Hz, 1H), 3.47 (d, *J* = 13.7 Hz, 1H), 2.16-1.83 (m, 7H), 1.52-1.37 (m, 2H), 1.32 (s, 3H), 1.08 (t, *J* = 7.5 Hz, 3H), 1.02 (s, 3H);

^{13}C NMR (125 MHz, $CDCl_3$) δ163.2, 150.1, 135.9, 132.8, 127.7, 127.1, 122.8, 119.5, 118.3, 114.5, 69.9, 66.0, 53.7, 48.1, 47.9, 45.2, 38.5, 33.3, 26.6, 22.1, 21.3, 20.00, 10.3;

$[\alpha]_D^{22}$ = +204 (*c* 0.152, $CHCl_3$);

HRMS (ESI) *m/z*: $[M+H]^-$ Cald for $C_{23}H_{27}BrN_2O_5S$, 523.0902; Found 523.0894.

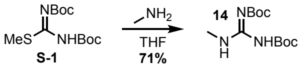

Compound 14

S-1 (164.44 g, 566.28 mmol, 1 equiv), methylamine (40 wt% solution in H_2O, 131.1 mL, 1699 mmol, 3 equiv), and THF (4719 mL) were combined in an air-dried 8 L round bottom flask equipped with magnetic stirring in open air. The reaction mixture was stirred overnight in open air. After, the reaction mixture was transferred to an Erlenmeyer flask, dried over $MgSO_4$, gravity filtered and concentrated *in vacuo*. The crude material was dissolved in 2627 mL 61.7 % MeOH/H_2O at 70 ˚C in a 4 L Erlenmeyer flask, in a Yamamoto BM200 heating bath, and allowed to cool to room temperature overnight. After, the flask was chilled in an ice-water bath for 30 min, and the product filtered on a large Büchner funnel, washed with 200 mL 60% MeOH/H_2O, then air-dried. Then, the product was spread out on filter paper on a warm fume hood base above a 120 ˚C oven to dry overnight, yielding a white powder. Yield = 110.63 g (71%).

CAUTION: This reaction produces methane thiol as a toxic and extremely odorous byproduct. The reaction, workup, and purification must all be completed in a fume hood to prevent exposure to methane thiol.

^{1}H NMR (500 MHz, $CDCl_3$) δ11.46 (s, 1H), 8.27 (s, 1H), 2.92 (d, J = 4.9 Hz, 3H), 1.47 (s, 9H), 1.45 (s, 9H);
^{13}C NMR (125 MHz, $CDCl_3$) δ163.5, 156.8, 153.3, 83.0, 79.3, 28.3, 28.0, 27.7;
HRMS (ESI) *m/z*: $[M+H]^+$ Cald for $C_{12}H_{23}N_3O_4$, 274.1767; Found 274.1762.

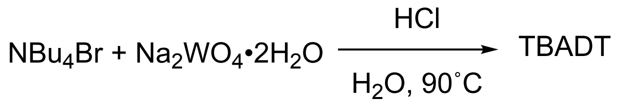

TBADT

A 4 L Erlenmeyer flask equipped with mechanical stirring was charged with NBu_4Br (19.2 g, 59.5 mmol, 1 equiv) and 1200 mL dH_2O. A separate 2 L Erlenmeyer flask equipped with mechanical stirring was charged with $Na_2WO_4 \cdot 2H_2O$ (40.0 g, 121 mmol, 2 equiv) and 1200 mL dH_2O. The 4 L flask was heated with stirring in a Yamamoto BM200 heating bath and the 2 L flask was heated in a hot-water bath, both to 90 ˚C, with stirring. Once both solutions reached 90 ˚C, both were adjusted to pH ~2 by addition of concentrated HCl. After, the $Na_2WO_4 \cdot 2H_2O$ solution was poured into the NBu_4Br solution through a glass funnel while hot, resulting in the precipitation of a white solid. The mixture was stirred for 30 min by hand, after which the reaction mixture was allowed to cool to room temperature in the bath. After, the reaction mixture was filtered on a 150 mL sintered glass funnel and allowed to air-day overnight on vacuum. After, the solid was washed with 800 mL dH_2O on the filter and allowed to air dry. After, the white solid was dried in an oven at 120 ˚C for 3 h. After, all the crude product was dissolved in MeCN and the aqueous and organic phases were separated in a separatory funnel, and the organic phase concentrated *in vacuo* and the crude product spread out over filter paper and dried in a 120 ˚C oven for 3 h. The crude product was suspended in 750 mL of CH_2Cl_2 and stirred vigorously for 2 h. After, the suspension was filtered and air-dried overnight to yield a white powder. Crop 1 = 14.9487 g. The uncollected material from the reaction flask and filtrate were combined and concentrated *in vacuo* and suspended in 460 mL of CH_2Cl_2 and stirred for 2 h. The 2nd crop of product was filtered on a 150 mL sintered glass funnel and allowed to air-dry overnight. Crop 2 = 13.6222 g.

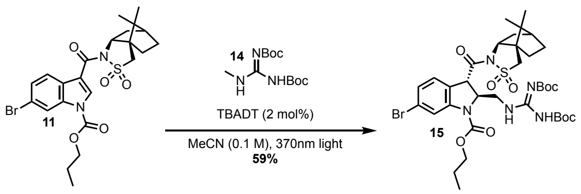

Compound 15

11 (3.94 g, 7.53 mmol, 1 equiv), **14** (10.31 g, 37.72 mmol, 5 equiv), and TBADT (500 mg, 0.2 mmol, 0.02 equiv) were added to a 40-mL Chemglass vial equipped with magnetic stirring. The flask was capped with a green cap equipped with a septum, evacuated and backfilled 3X with argon, and sparged MeCN (37.7 mL) was added. The vial was sealed with parafilm. The vials were irradiated with 370 nm Generation 2 Kessil lamps at 100% power at 2 cm distance with cooling by SUNON fans and stirring overnight. 8 vials were run in a single batch. This procedure was repeated for a total of 66.37 g. After, all vials were pooled together and dried *in vacuo* to yield a blue-grey, amorphous solid. The crude material was suspended in EtOAc and separated from TBADT by running it through a 1400 g celite plug by gravity. The EtOAc fractions were dried down, and the crude material was suspended in 1658 mL MeOH at room temperature across three portions, and **15** was allowed to precipitate over an h. After, the product was filtered on a Büchner funnel, and the filter cake was washed with 350 mL of ice-cold MeOH to afford a white powder. Yield = 59.92 g (59%).

^{1}H NMR (500 MHz, $CDCl_3$) δ11.37 (s, 1H), 8.47 (s, 1H), 8.08-7.54 (m, 1H), 7.31 (d, J = 8.6 Hz, 1H), 7.05 (d, J = 7.8, 1H), 4.99 (t, J = 5.3 Hz, 1H), 4.65-4.46 (bs, 1H), 4.32-4.11 (m, 2H), 3.86 (dd, J = 8.4, 4.6 Hz, 1H), 3.77 (m, 1H), 3.64 (m, 1H), 3.55 (d, J = 13.8 Hz, 1H), 3.47 (d, J = 13.8 Hz, 1H), 1.97-1.69 (m, 7H), 1.47 (s, 9H), 1.43 (s, 9H), 1.12, (s, 3H), 0.99 (m, 3H), 0.96 (s, 3H);

^{13}C NMR (125 MHz, $CDCl_3$) δ170.2, 163.2, 156.7, 152.9, 152.5, 126.7, 126.4, 126.1, 123.2, 119.5, 83.0, 79.2, 67.7, 65.2, 61.8, 53.3, 49.6, 48.5, 47.8, 44.4, 42.9, 37.9, 32.8, 28.3, 28.0, 26.4, 22.1, 20.8, 19.9, 10.4;

$[\alpha]_D^{22}$ = +160 (c 0.275, $CHCl_3$);

HRMS (ESI) *m/z*: $[M+H]^+$ Cald for $C_{35}H_{50}BrN_5O_9S$, 796.2591; Found 796.2602.

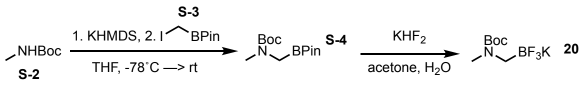

Compound 20

A flame-dried 3-neck 3 L round bottom8 flask equipped with magnetic stirring and a flame-dried 1L addition funnel was charged with **S-2** (57.919 g, 441.56 mmol, 1 equiv), and it was dissolved in 830 mL of dry THF. The solution was chilled to -78 ˚C in an acetone/dry ice bath under argon, and KHMDS ([0.7] in toluene, 637 mL, 446 mmol, 1.01 equiv) was quickly and carefully poured into the addition funnel in open air under a stream of argon. The KHMDS was added at -78 ˚C as a heavy stream through the funnel, and KHMDS crystallized on the sides of the flask was washed down with 100 mL of dry THF. After, the solution was allowed to stir at -78 ˚C for one h. After, a solution of **S-3** (118.293 g, 441.56 mmol, 1 equiv) was added as a solution in 125 mL dry THF via cannula as a steady stream, and the flask containing **S-3** was washed with an additional 10 mL dry THF, which was transferred to the reaction mixture. After, the cooling bath was removed and the reaction mixture allowed to warm to room temperature overnight with stirring under Ar. After, the reaction mixture was quenched with 415 mL sat. aq NH_4Cl, the aqueous and organic phases were separated, and the aqueous phase was extracted with EtOAc (3 x 400 mL). The combined organics were washed with sat. aq brine (2L), dried over $MgSO_4$, gravity filtered, and concentrated *in vacuo* to afford **S-4** as a light-brown oil. The crude product was used without any further purification for the next step. Mass yield = 139.28 g.

^{1}H NMR (500 MHz, $CDCl_3$) δ2.82 (s, 3H), 2.40 (s, 2H), 1.49 (s, 9H), 1.19 (s 12H).

A 4 L Erlenmeyer flask equipped with magnetic stirring was charged with crude **S-4**. Crude **S-4** was dissolved in acetone (1020 mL) and H_2O (345 mL), and KHF_2 (119.40 g, 1529 mmol, 3 equiv) was added in open air. The flask was capped, and the reaction mixture stirred vigorously in open air overnight. After, the reaction mixture was transferred to a 2 L round bottom flask and concentrated *in vacuo*. The resulting pasty solid was taken up in toluene and azeotroped three times to remove excess water. Toluene was meticulously removed by breaking up the crude solid product mechanically and drying the solid at 65 ˚C until no more solvent was observed condensing on the rotary evaporator dry ice/acetone trap. After, the extracts were suspended in 1700 mL Et_2O with stirring, filtered on a

Büchner funnel, and washed with another two 600 mL portions of Et_2O, and air-dried to yield a white powder. Yield = 67.23 g (53% over two steps).

^{1}H NMR (500 MHz, acetone-d_6) δ2.81 (s, 3H), 2.29 (s, 2H), 1.39 (s, 9H);

^{13}C NMR (125 MHz, CD_3OD) δ158.6, 79.5, 41.4, 35.8, 29.00;

HRMS (ESI) *m/z*: $[M+H]^-$ Cald for $C_7H_{13}BF_3KNO_2$, 212.107517; Found 212.107576.

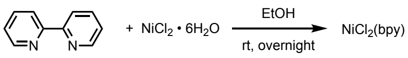

$NiCl_2$(bpy)

Two flame-dried 500-mL round bottom flasks equipped with magnetic stirring were charged with 2,2'-bypridine (5.714 g, 36.59 mmol, 1 equiv) and $NiCl_2$ • 6 H_2O (8.686 g, 36.54 mmol, 1 equiv), then both flasks charged with 114 mL 100% EtOH. After 2,2'-bypyridine had dissolved, it was poured via glass funnel into the solution of $NiCl_2$ • $6H_2O$. The reaction mixture was stirred under argon overnight, after which it was the resulting green precipitate was filtered on a 150-mL sintered-glass funnel and washed with 100% EtOH (3 x 170 mL0, then air-dried overnight. After, the product was transferred to scintillation vials and dried under high vacuum overnight to afford $NiCl_2$(bpy) as a light-green powder. Yield = 5.5913 g (43%).

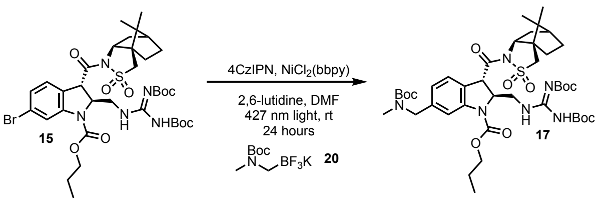

Compound 17

15 (3.00 g, 3.77 mmol, 1 equiv), **20** (1.89 g, 7.53 mmol, 2 equiv), 4-CzIPN (45 mg, 0.057 mmol, 1.5 mol%), and $NiCl_2$bpy (270 mg, 0.75 mmol, 20 mol%) were added to a 40-mL Chemglass vial equipped with magnetic stirring. The flask was sealed with a cap equipped with a septum, evacuated and backfilled 3X with argon, and sparged DMF (37.3 mL) was added, followed by 2,6-lutidine (1.53 mL, 13.2 mmol, 3.5 equiv). The vial was sealed with parafilm. 8 vials were run in a single batch, and the vials were irradiated with 427 nm Kessil lamps at 100% power at 2cm distance with cooling by SUNON fans and stirring overnight. This process was repeated for a total of 63 g of **15**. After, all vials were gravity filtered into 1L sat. aq brine, the aqueous phase extracted with EtOAc (3 x 1.5L), then combined organics washed with sat. aq brine (6 x 2L), dried over $MgSO_4$, gravity filtered and concentrated. The crude material was loaded onto 210 g of celite and purified by silica gel chromatography (25% EtOAc/Hexanes, R_f = 0.27) to yield a white powder. Yield = 61 g.

^{1}H NMR (500 MHz, $CDCl_3$, 24.85˚C) δ11.40 (bs, 1H), 8.54 (bs, 1H), 7.86-7.44 (m, 1H), 7.37 (d, *J* = 7.1 Hz, 1H), 6.91-6.70 (m, 1H), 5.01 (m, 1H), 4.66-4.08 (m, 5H), 3.84-3.69 (m, 2H), 3.66-3.50 (m, 2H), 3.46 (d, *J* = 13.8 Hz, 1H), 2.88-2.65 (m, 3H), 1.97-1.70 (m, 7H), 1.52-1.39 (m, 27H), 1.35-1.20 (m, 2H), 1.14 (s, 3H), 1.03-0.90 (m, 6H);

^{1}H NMR (500 MHz, $CDCl_3$, 34.85˚C) δ11.40 (bs, 1H), 8.54 (bs, 1H), 7.86-7.44 (m, 1H), 7.37 (d, *J* = 7.1 Hz, 1H), 6.91-6.70 (m, 1H), 5.01 (t, *J* = 5.7Hz, 1H), 4.66-4.08 (m, 5H), 3.84-3.69 (m, 2H), 3.66-3.50 (m, 2H), 3.46 (d, *J* = 13.8 Hz, 1H), 2.88-2.65 (s, 3H), 1.97-1.70 (m, 7H), 1.52-1.39 (m, 27H), 1.35-1.20 (m, 2H), 1.14 (s, 3H), 1.03-0.90 (m, 6H);

^{1}H NMR (500 MHz, $CDCl_3$, 50˚C) δ11.40 (bs, 1H), 8.54 (bs, 1H), 7.86-7.44 (m, 1H), 7.37 (d, *J* = 7.9 Hz, 1H), 6.91-6.70 (m, 1H), 5.03 (t, *J* = 6.1 Hz, 1H), 4.63-4.23 (m, 4H), 4.22-4.13 (m, 1H), 3.94-3.79 (m, 2H), 3.68-3.59 (m, 1H),

3.59-3.52 (m, 1H), 3.46 (d, $J = 13.8$ Hz, 1H), 2.81 (s, 3H), 1.97-1.70 (m, 7H), 1.52-1.39 (m, 27H), 1.35-1.20 (m, 2H), 1.14 (s, 3H), 1.03-0.90 (m, 6H);

^{13}C NMR (125 MHz, $CDCl_3$, 24.85°C) δ170.8, 156.7, 156.2, 155.8, 152.9, 142.5, 139.7, 126.7, 125.5, 122.7, 122.0, 115.9, 83.0, 79.8, 79.6, 79.4, 67.6, 65.3, 61.6, 53.5, 52.8, 52.0, 49.9, 48.5, 47.8, 44.5, 43.4, 38.1, 34.0, 32.9, 28.5, 28.3, 28.1, 26.5, 22.2, 20.9, 19.9, 10.5;

^{13}C NMR (125 MHz, $CDCl_3$, 50°C) δ171.0, 163.2, 156.8, 139.9, 125.5, 115.9, 83.1, 79.8, 79.4, 67.8, 65.5, 62.1, 53.5, 50.0, 48.7, 48.0, 44.8, 43.5, 38.2, 38.1, 34.1, 33.1, 33.0, 29.8, 28.6, 28.5, 28.2, 26.7, 22.4, 21.1, 20.9, 20.0; 10.5;

$[\alpha]_D^{22}$ = +135 (*c* 135, $CHCl_3$);

HRMS (ESI) *m/z*: $[M+Na]^+$ Cald for $C_{42}H_{64}N_6O_{11}S$, 883.4251; Found 883.4238.

Note: 1. At room temperature, the ^{1}H and ^{13}C spectra include signals from hydrogen and carbon atoms occupying multiple signal populations, possibly due to rotational restrictions. Variable-Temperature ^{1}H NMR experiments result in some signals coalescing into singlets or sharpening, revealing more clearly defined spin-spin coupling. Variable-Temperature ^{13}C NMR experiments result in the coalescing of several signals that is consistent with the number and type of carbon atoms present.

2. Percent yield is not reported in this procedure because of impurities present in the product. Pure **17** was obtained previously on smaller scales, for which data is reported here.

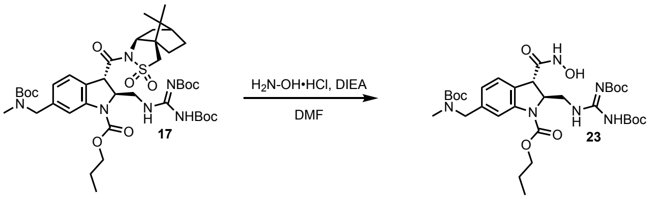

Compound 23

An air-dried 3 L round bottom flask equipped with magnetic stirring was charged with **17** (59.650 g, 69.274 mmol, 1 equiv), H_2N-OH•HCl (168.5 g, 2425 mmol, 35 equiv), DMF (346 mL), and DIEA (422 mL, 2430 mmol, 35 equiv), the flask was sealed with a septum and parafilm, and the mixture was vigorously stirred for 6 h. After, the reaction mixture was poured into 3750 mL dH_2O, leading to the precipitation of a gummy solid. The precipitate was filtered on a fritted funnel and allowed to dry. After, the gummy solid was washed off the filter with CH_2Cl_2, along with remaining precipitate on the walls of the round bottom flask. The CH_2Cl_2 extracts were dried over $MgSO_4$, gravity filtered, and concentrated. The crude material was suspended in minimal 30% EtOAc/Hexanes & loaded on a 1110 g silica plug, eluting first with 30% EtOAc/Hexanes to elute free camphorsultam, followed by flushing with 10% MeOH/CH_2Cl_2 to elute **23**. The MeOH/CH_2Cl_2 fractions were dried down to yield a foamy red solid, which was used in the next step without further purification. Yield = 38.92 g.

^{1}H NMR (500 MHz, $CDCl_3$) δ11.28 (s, 1H), 10.59 (bs, 1H), 9.09-8.18 (m, 2H), 7.58 (s, 1H), 7.08 (m, 1H), 6.89 (m, 1H), 4.48 (d, J = 7.6 Hz, 1H), 4.39 (m, 2H), 4.22 (t, J = 6.5 Hz, 2H), 4.13 (m, 1H), 4.03 (m, 2H), 2.79 (m, 3H), 1.76 (m, 2H), 1.50-1.42 (m, 27H), 0.99 (t, J = 7.4 Hz, 3H).

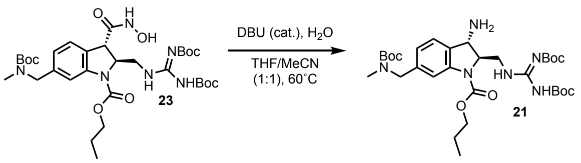

Compound 21

An air-dried 2 L round bottom flask equipped with magnetic stirring was charged with **23** (38.680 g, 56.984 mmol, 1 equiv), THF/MeCN (690 mL), and H_2O (62 mL, 3400 mmol, 60 equiv) in open air. The flask was fitted with a reflux condenser, purged with Ar, and carefully heated to 60 ˚C in a hot-water bath. Once the reaction mixture reached temperature, the reflux condenser was very quickly removed and 1,8-Diazabicyclo[5.4.0]undec-7-ene (DBU) (8.52 mL, 57.0 mmol, 1 equiv) added, and the reflux condenser immediately replaced. The reaction mixture was stirred at 60 ˚C for 2.5 h, after which the reaction mixture was concentrated *in vacuo* and on high vacuum to remove excess water. The crude, red gel-like solid was taken up in 885 mL Et_2O, washed with 885 mL 0.5 M HCl, and the aqueous phase and organic phase separated. The aqueous phase was **quickly** poured into 442 mL 1 M NaOH, and further basified to pH ~14. The aqueous phase was extracted with EtOAc (4 x 885 mL), and the combined organics dried over $MgSO_4$, gravity-filtered, and concentrated *in vacuo* to yield an off-white, foamy solid, which was used in the next reaction without further purification. Yield = 16.66 g.

Note: Precise temperature is crucial to obtaining the best yield!

^{1}H NMR (500 MHz, $CDCl_3$) δ11.39 (s, 1H), 8.48 (s, 1H), 7.88-7.42 (s, 1H), 7.23 (d, J = 7.8 Hz, 1H), 6.90 (m, 1H), 4.49-4.12 (m, 7H), 4.10 (s, 1H), 2.79 (m, 3H), 1.76 (m, 2H), 1.49-1.41 (m, 27 H), 0.98 (t, J = 7.42 Hz, 3H).

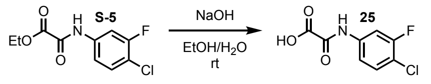

Compound 25

An air-dried 1 L round bottom flask equipped with magnetic stirring was charged with 1:1 EtOH:H_2O (370 mL) and NaOH (5.03 g, 126 mmol, 2 equiv) with stirring. **S-5** (15.445 g, 62.878 mmol, 1 equiv) was added, upon which the reaction mixture almost instantly solidified. An additional 65 mL of EtOH was added and stirring was increased to facilitate better mixing. The reaction mixture was stirred for 6 h, after which it was cooled to 0 ˚C in an ice/water bath and acidified to pH ~1 with 2 M HCl. The precipitate was filtered on a Büchner funnel, washed with 300 mL H_2O, and air-dried overnight to afford a white powder. Yield = 11.91 g (87%). Spectral data matches that previously reported data for **25**.[2]

^{1}H NMR (500 MHz, DMSO-d$_6$) δ10.83 (s, 1H), 7.93 (dd, *J* = 12.1 Hz, 1.5 Hz, 1H), 7.67 (d, *J* = 8.7 Hz, 1h), 7.52 (t, *J* = 8.6 Hz, 1H);

^{13}C NMR (125 MHz, DMSO-d$_6$) δ162.1, 161.0, 156.9 (d, *J* = 243.77 Hz), 139.0 (d, *J* = 10.12 Hz), 130.3, 117.0 (d, *J* = 2.98 Hz), 113.7 (d, *J* = 17.66 Hz), 108.1 (d, *J* = 25.53 Hz).

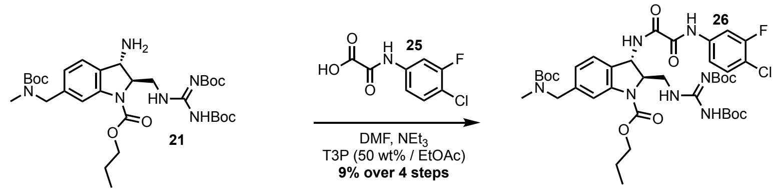

Compound 26

An air-dried 1 L round bottom flask equipped with magnetic stirring was charged with **21** (16.4 g, 25.8 mmol, 1 equiv), **25** (5.621 g, 25.84 mmol, 1 equiv), and the flask was sealed with a septum and evacuated and backfilled 3X with Ar. Then, DMF (182 mL), NEt_3 (10.8 mL, 77.5 mmol, 3 equiv), and T3P (11.53 mL, 19.38 mmol, 0.75 equiv) were added in that order. The reaction mixture was stirred overnight. After, the reaction mixture was poured into 300 mL 5 wt% aq LiCl, and the aqueous phase extracted with EtOAc (3x 300 mL). The combined organic layers were washed with 5 wt% aq LiC (3 x 300 mL), then 1L sat. aq brine, dried over $MgSO_4$, gravity filtered, and concentrated *in vacuo*. The crude material was

purified by flash column chromatography (20 % EtOAc/Hexanes, $R_f = 0.2$) to yield an off-white powder.

Yield = 5.66g (9% from **17**)

^{1}H NMR (600 MHz, $CDCl_3$, 26.85˚C) δ11.42 (s, 1H), 9.24 (s, 1H), 8.58 (bs, 1H), 7.89-7.55 (m, 3H), 7.37 (t, *J* = 8.3 Hz, 1H), 7.21 (dd, *J* = 8.6, 1.4 Hz, 1H), 7.03-6.87 (m, 1H), 5.14 (d, *J* = 7.2 Hz, 1H), 4.62 – 4.36 (m, 3H), 4.23 (m, 2H), 3.89 (bs, 1H), 3.77-3.63 (m, 1H), 2.94-2.74 (m, 3H), 1.79 (m, 2H), 1.52-1.44 (m, 27H), 1.00 (t, *J* = 6.6 Hz, 3H);

^{1}H NMR (500 MHz, $CDCl_3$, 36.85˚C) δ11.39 (s, 1H), 9.26 (s, 1H), 8.54 (bs, 1H), 7.89-7.55 (m, 3H), 7.37 (t, *J* = 8.21 Hz, 1H), 7.21 (m, 1H), 7.03-6.87 (bs, 1H), 5.14 (d, *J* = 7.62 Hz, 1H), 4.62 – 4.36 (m, 3H), 4.23 (m, 2H), 3.89 (m, 1H), 3.77-3.63 (m, 1H), 2.82 (s, 3H), 1.78 (m, 2H), 1.52-1.44 (m, 27H), 1.00 (t, *J* = 7.42 Hz, 3H);

^{1}H NMR (500 MHz, $CDCl_3$, 50˚C) δ11.39 (s, 1H), 9.24 (s, 1H), 8.54 (bs, 1H), 7.89-7.55 (m, 3H), 7.36 (t, *J* = 8.25 Hz, 1H), 7.21 (m, 1H), 6.94 (d, *J* = 6.46 Hz, 1H), 5.17 (d, *J* = 7.54 Hz, 1H), 4.62 – 4.36 (m, 3H), 4.23 (m, 2H), 3.89 (m, 1H), 3.77-3.67 (m, 1H), 2.82 (s, 3H), 1.79 (sx, *J* = 7.16 Hz, 2H), 1.52-1.44 (m, 27H), 1.00 (t, *J* = 7.46 Hz, 3H);

^{13}C NMR (125 MHz, $CDCl_3$, 26.85˚C) δ159.0, 158.1 (d, *J* = 248.31 Hz), 157.1, 157.0, 156.5, 156.3, 152.9, 141.4,138.1, 136.1 (d, *J* = 9.58 Hz),131.0, 126.8, 126.1, 123.4, 122.6, 117.3 (d, *J* = 17.20 Hz), 115.9 (d, *J* = 3.47 Hz), 115.6, 108.4 (d, *J* = 26.26 Hz), 83.9, 80.1, 79.8, 68.2, 65.9, 65.8, 53.9, 53.5, 52.9, 52.2, 43.6, 34.6, 34.1, 34.0, 31.1, 28.6, 28.3, 28.1, 22.3, 10.6;

^{13}C NMR (125 MHz, $CDCl_3$, 50˚C) δ163.3, 159.0, 158.4 (d, *J* = 248.83 Hz), 157.2, 156.9, 153.1, 153.0, 141.5, 136.3 (d, *J* = 9.09 Hz), 131.1, 126.6, 126.0, 122.9, 117.5 (d, *J* = 18.09 Hz), 116 (d, *J* = 3.64 Hz), 115.5, 108.7 (d, *J* = 25.49 Hz), 83.4, 79.9, 79.6, 68.1, 66.1, 54.0, 52.6, 43.0, 34.1, 31.4, 29.8, 28.6, 28.5, 28.2, 22.3;

$[\alpha]_D^{22}$ = +17.2 (*c* 2.33, $CHCl_3$);

HRMS (ESI) *m/z*: $[M+H]^+$ Cald for $C_{39}H_{54}ClFN_7O_{10}$, 834.359923; Found 834.361533.

Note: At room temperature, the ^{1}H and ^{13}C spectra include signals from hydrogen and carbon atoms occupying multiple signal populations, possibly due to rotational restrictions. Variable-Temperature ^{1}H NMR experiments result in some signals coalescing into singlets or sharpening, revealing more clearly defined spin-spin coupling. Variable-Temperature ^{13}C NMR experiments result in the coalescing of several signals that is consistent with the number and type of carbon atoms present.

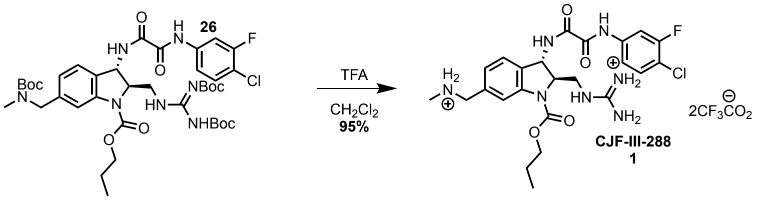

Compound 1

A half-liter round-bottom flask equipped with magnetic stirring was charged with **26** (5.66 g, 6.78 mmol, 1 equiv,), and the flask was purged with Ar, followed by addition of CH_2Cl_2 (67.8 mL). The reaction mixture was cooled to 0 ˚C in an ice/water bath, after which TFA (15.6 mL, 203 mmol, 30 equiv) was added. The ice bath was removed, and the reaction mixture was allowed to warm to room temperature with stirring overnight. After, the reaction mixture was concentrated *in vacuo*, and the crude material loaded onto 30 g of celite and purified by column chromatography (15 % MeOH/CH_2Cl_2 + 0.4% TFA, R_f = 0.25) to yield a red, mobile oil. The product was continuously taken up in methanol and concentrated and dried on high-vacuum until it became an immobile oil. After, the product was taken up in Et_2O with mixing and stirring, and the Et_2O carefully decanted to yield an off-white, amorphous solid. Yield = 4.90437 g (95%). Spectral data matches that previously reported for **1**.[3]

^{1}H NMR (500 MHz, CD_3OD) δ8.05-7.75 (m, 2H), 7.52-7.41 (m, 3H), 7.22 (d, *J* = 7.6, 1H), 5.24 (s, 1H), 4.56 (m, 1H), 4.33-4.17 (m, 4H), 3.59 (dd, *J* = 14.1, 5.3 Hz, 1H), 3.53 (dd, *J* = 13.9, 7.0 Hz, 1H), 2.73 (s, 3H), 1.81 (m, 2H), 1.03 (t, *J* = 7.4 Hz, 3H)

^{13}C NMR (125 MHz, CD_3OD) δ162.9, 161.2, 159.3, 159.3, 159.0 (d, *J* = 245.50 Hz), 154.7, 144.4, 138.9 (d, *J* = 9.67 Hz), 134.5, 131.6, 131.2, 127.8, 126.4, 119.3, 118.4, 118.1 (d, *J* = 3.49 Hz), 117.2 (d, *J* = 18.24 Hz), 109.7 (d, *J* = 26.09 Hz), 69.4, 66.8, 54.4, 53.5, 44.3, 33.0, 23.2, 10.7;

$[\alpha]_D^{22}$ = +39.9 (*c* 1.18, MeOH);

HRMS (ESI) *m/z*: $[M+H]^+$ Cald for $C_{24}H_{30}ClFN_7O_4$, 534.202635; Found 534.202792.

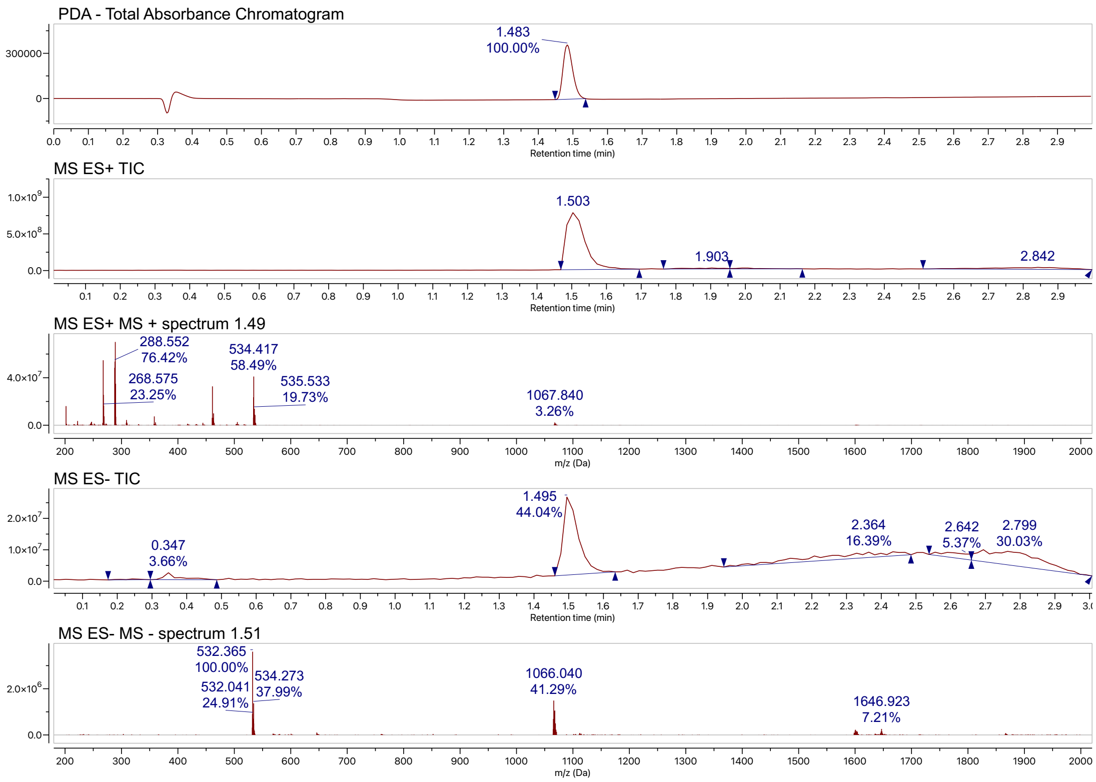

Figure S-2 LC-MS Data for CJF-III-288.

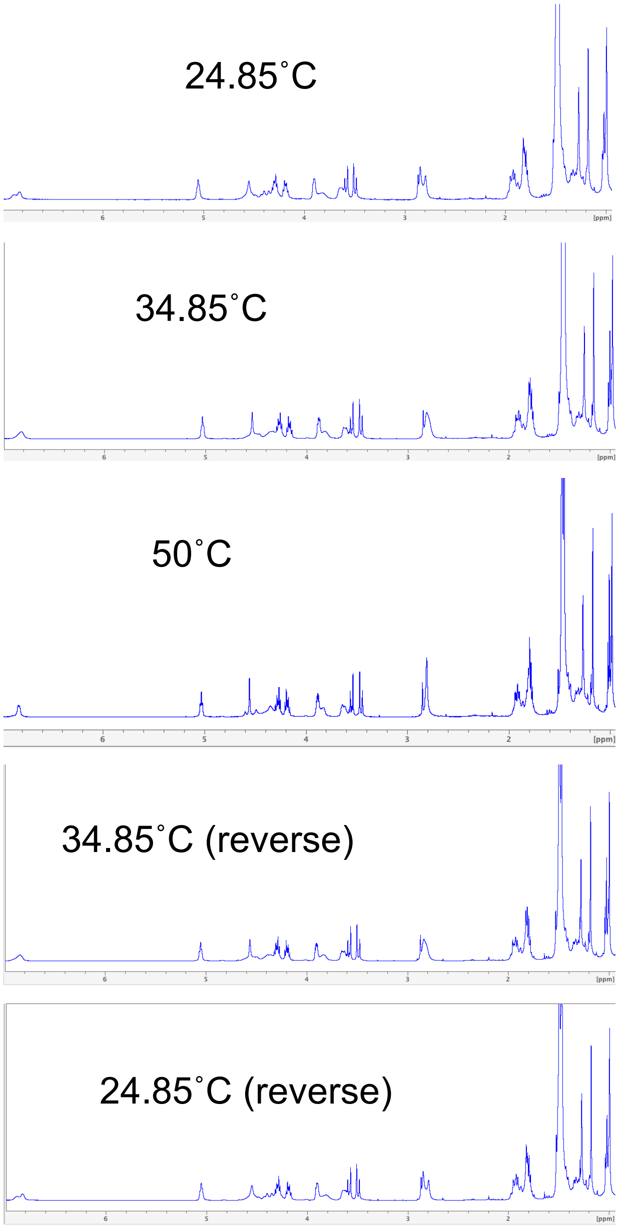

Figure S-3 Segment of the Variable-Temperature NMR experiment for compound 17. The sample was slowly heated, and spectra were collected at 24.85˚C, 34.85˚C, 50˚C, and then the same temperatures in the reverse direction while allowing the sample to cool to demonstrate the reversibility of the process. The coalescence of the methyl proton signal into a singlet and peak sharpening of other signals provides evidence for rotational constriction being the cause of peak broadness in the ^{1}H NMR spectra, and the excessive number of ^{13}C signals in the room-temperature ^{13}C spectrum. Please note, an unknown impurity

appearing in later batches of compound **17** appears at δ2.84 and overlaps with the methyl singlet at δ2.81. Attempts at removing this impurity via silica gel chromatography were unsuccessful.

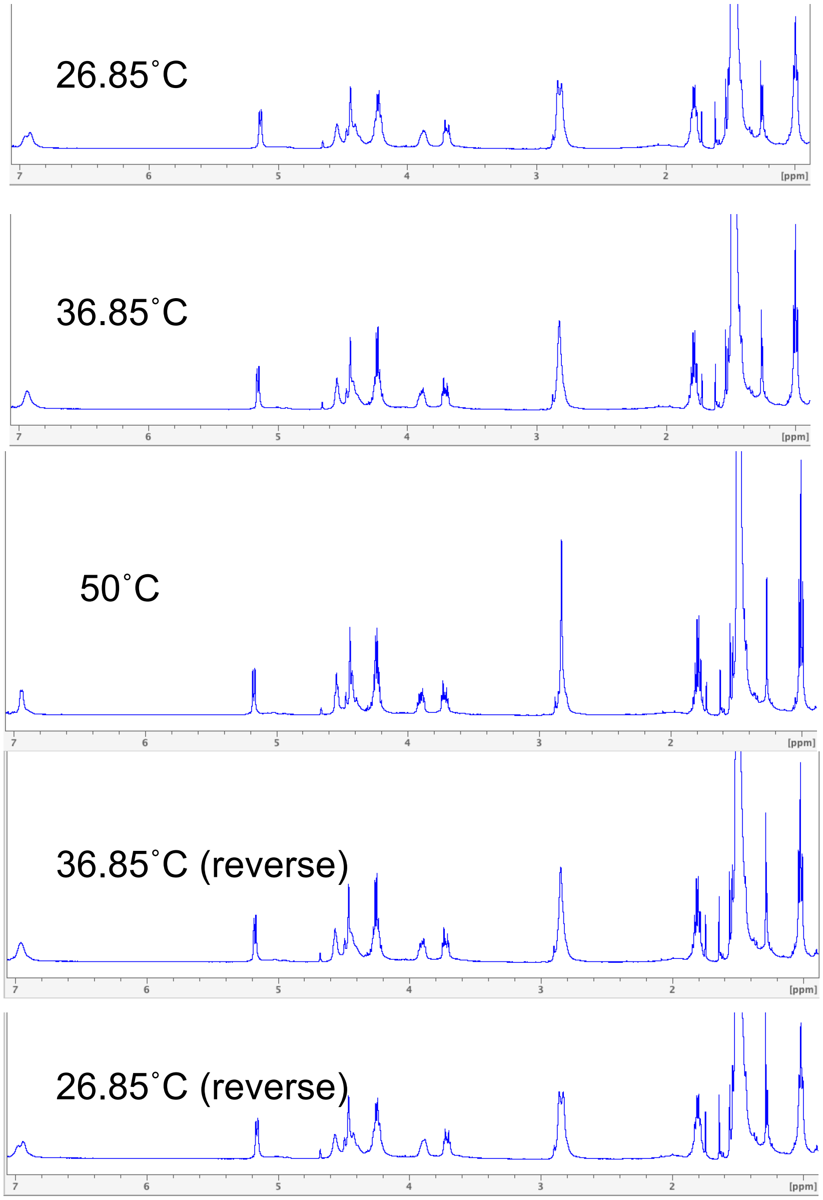

Figure S-4 Segment of the Variable-Temperature NMR experiment for compound 26. The sample was slowly heated, and spectra were collected at 26.85˚C, 36.85˚C, 50˚C, and then the same temperatures in the reverse direction while allowing the sample to cool to demonstrate the reversibility of the process. The coalescence of the methyl proton signal into a singlet and peak sharpening of other signals provides evidence for rotational constriction being the cause of peak broadness in the ^{1}H NMR spectra, and the excessive number of ^{13}C signals in the room-temperature ^{13}C spectrum.

3. Additional Discussion of the Optimization of the Giese Addition

The conversion to **15** was optimized (see Figures S-5 through S-7). The first investigation was into the reaction solvent using liquid chromatography area percent (LCAP) as a guide for observing changes in yield. Using five equivalents of Giese donor **14** and 2 mol% of TBADT, a solvent screen was performed. Coincidentally, this investigation was the first time **14** was purified before use, revealing that a major factor for the low conversion described previously was insoluble impurities present. The solvent screen revealed the very limited solubility of TBADT. The catalyst only had appreciable solubility in acetonitrile and acetone; in all other solvents it was totally insoluble, observable visually and evident by the complete lack of conversion to **15**. Full consumption of **11** was observed in both acetonitrile and acetone, but the greatest conversion to **15** was observed in acetonitrile. Additional investigations were made to further optimize reaction conditions. Decreasing or increasing the reaction concentration had no positive affect, but it was discovered that the reaction can be run at 0.2 molarity instead of 0.1, which improves the ease of scale-up. At the scales used for this synthesis, changing the lamp distance and light intensity had no effect on the reaction. Solvent effects were further investigated by the addition of a cosolvent in a 1:4 ratio with acetonitrile. At best, no change was observed to the conversion by LC-MS. In the worst cases, conversion stagnated or protodehalogenation began to predominate (toluene, DMSO, nitromethane), or addition of the solvent into **11** resulted in a greatly diminished yield (THF, DMA, NMP). The addition of 15 mol % of a Lewis Acid was used to probe the effects of disrupting or enhancing the transition state proposed for radical addition into camphorsultam-bearing indoles.[9] No effect was observed on the diastereoselectivity of the reaction, but conversion deteriorated, possibly due to the insolubility of the additives in MeCN. The addition of 30 mol % of tetrabutylammonium salts was used to investigate counteranion effects, but no improvement in yield by LC-MS was observed.

The efficient isolation of **15** proved a formidable challenge. The use of a large excess of radical donor **14**, with similar retention to **15** on silica-gel, made chromatography untenable on scale. Initially, slow evaporation from acetone was found to be a reliable method to separate **15** from **14** in high

diastereoselectivity, but as a days-long process was incompatible with creating an expeditious synthesis of **1** on scale. Pleasingly, during solubility investigations, the trituration of **15** from methanol was discovered, facilitating the rapid isolation of **15** without recrystallization or chromatography.

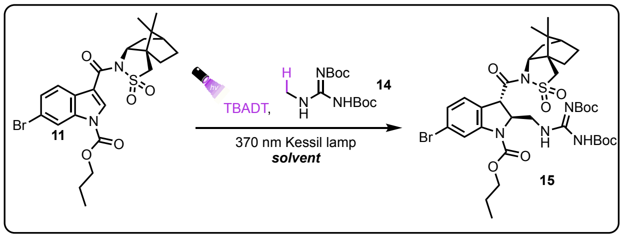

Entry	solvent	LCAP[a]
1	Acetonitrile	84
2	Ethyl Acetate	0
3	Acetone	60
4	Tert-butanol	0
5	Trifluorotoluene	0
6	Hexafluoroisopropanol	0
7	Trifluoroethanol	0
8	Benzene	0

[a]Liquid chromatography area percentage of **15**.

Figure S-5 Optimization of the Giese Addition

Entry	Solvent	Molarity	Lamp Distance	Additive	LCAP[a]
1	MeCN[b]	0.1	2 cm	-	84
2	MeCN[b]	0.05	2 cm	-	77
3	MeCN[b]	0.2	2 cm	-	76
4	MeCN[b]	0.1	5 cm	-	83
5	MeCN[b]	0.1	10 cm	-	83
6	MeCN[b]	0.1	2 cm	-	77
7	MeCN[c]	0.1	2 cm	PhCN	83
8	MeCN[c]	0.1	2 cm	Toluene	50
9	MeCN[c]	0.1	2 cm	TFT	83
10	MeCN[c]	0.1	2 cm	TFE	83
11	MeCN[c]	0.1	2 cm	Acetone	83
12	MeCN[c]	0.1	2 cm	THF	1
13	MeCN[c]	0.1	2 cm	DMA	1
14	MeCN[c]	0.1	2 cm	DMSO	8
15	MeCN[c]	0.1	2 cm	DCE	83

[a]Liquid Chromatography Area Percentage of 15.
[b]MeCN dried over 4Å molecular sieves or dry MeCN purchased from Millipore-Sigma.
[c]Reagent-grade MeCN used without further drying.

Figure S-6 Additional optimization of the Giese Addition

Entry	Solvent	Molarity	Lamp Distance	Additive	LCAP[a]
15	MeCN[c]	0.1	2 cm	DCE	83
16	MeCN[c]	0.1	2 cm	*t*-BuOH	82
17	MeCN[c]	0.1	2 cm	CH_3NO_2	16
18	MeCN[c]	0.1	2 cm	NMP	0
19	MeCN[c]	0.1	2 cm	$EuCl_3$ (15 mol%)	40
20	MeCN[c]	0.1	2 cm	$SmCl_2$ (15 mol%)	20
21	MeCN[c]	0.1	2 cm	$ScCl_3$ (15 mol%)	30
22	MeCN[c]	0.1	2 cm	$MgCl_2$ (15 mol%)	38
23	MeCN[c]	0.1	2 cm	TBACl	72
24	MeCN[c]	0.1	2 cm	TBAB	79
25	MeCN[c]	0.1	2 cm	TBAI	66
26	MeCN[c]	0.1	2 cm	$TBAPF_6$	69
27	MeCN[c]	0.1	2 cm	$TBAClO_4$	74

[a]Liquid Chromatography Area Percentage of 15.
[b]MeCN dried over 4Å molecular sieves or dry MeCN purchased from Millipore-Sigma.
[c]Reagent-grade MeCN used without further drying.

Figure S-7 Additional optimization of the Giese Addition (Continued)

4. Additional Discussion of the Optimization of the Photoredox Cross-Coupling

An optimization of the photoredox cross-coupling was performed (see Figures S-8 through S-10). Conversion (LCAP) improved to nearly 70% upon increasing the equivalents of **20** from 1.2 to 2, but increasing the equivalents any further resulted in a decrease in consumption of **15** and enhanced protodehalogenation. Next, the effect of the ligand for the nickel catalyst was investigated. Utilizing electronically neutral ligands such as 2,2'-bipyridine and 1,10-phenanthroline achieved the greatest LCAP by LC-MS analysis (72% and 53%, respectively), while electronically deficient and rich ligands achieved no conversion. This effect may be due to the need to keep the metal center sufficiently electronically balanced and capable of both oxidative addition and reductive elimination during the catalytic cycle. Next, the balance between photo- and metal cocatalysts was investigated. When CzIPN is present in greater quantities than the nickel catalyst, protodehalogenation begins to predominate. The ideal balance was determined to be 1.5 mol% CzIPN to 12 mol% of Ni catalyst. Increasing the nickel loading to 20 mol% was found to be necessary to maintain good yields on scale. Finally, use of the nickel precatalyst $NiCl_2$(bpy) instead of *in-situ* formation of the liganded nickel catalyst was found to achieve full consumption of **15**, and the desired adduct **17** was obtained in 75% yield after flash column chromatography on 7.6 mmol scale.

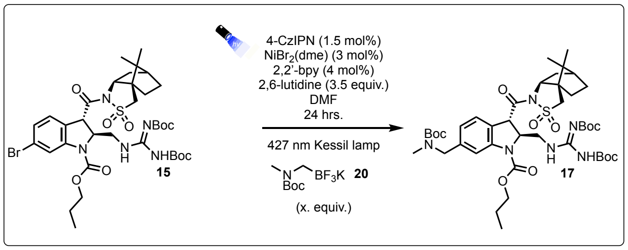

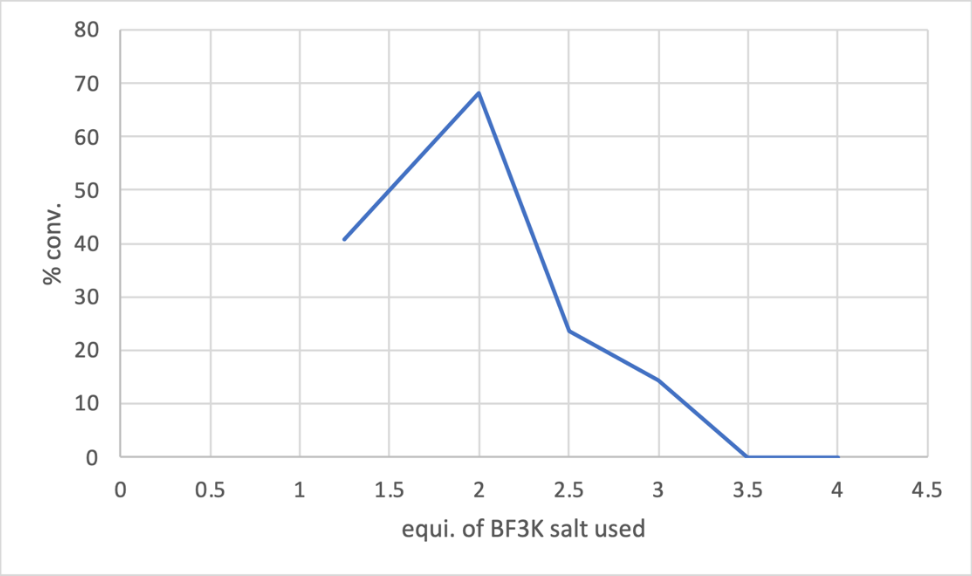

Figure S-8 Optimization of the equivalents of trifluoroborate in the photoredox cross-coupling

Entry	Ligand (4 mol%)	LCAP[a]
1	2,2-bipyridine	72
2	phen	53
3	4,4'-*t*Bu_2bpy	0
4	5,5'-dCF_3bpy	0
5	MeO_2bpy	0

[a]Liquid chromatography area percentage of **17**.

Figure S-9 Optimization of the nickel catalyst ligand in the photoredox cross coupling

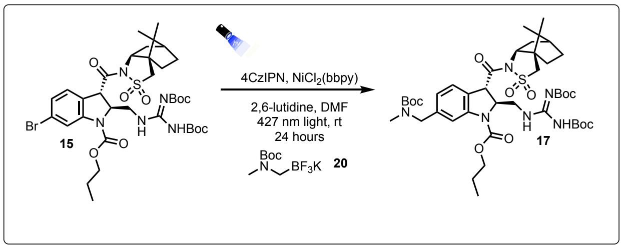

Entry	$NiCl_2$(bpy) (mol %)	4CzIPN (mol %)	CC/ PDH[a]
1	3	1.5	3.77
2	6	1.5	4.89
3	9	1.5	5.67
4	12	1.5	6.15
5	15	1.5	6.15
6	3	3	2.34
7	3	4.5	1.17
8	3	6	0.3
9	3	6.5	0
10	3	7	0

[a]The LCAP ratio of cross-coupled (CC) product **17** to protodehalogenation byproduct (PDH) was compared.

Figure S-10 Optimization of the ratio of nickel catalyst and photocatalyst in the photoredox cross coupling

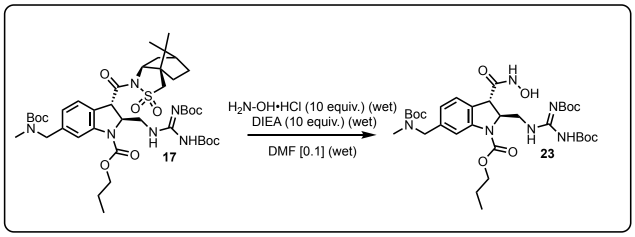

Entry	Solvent	Equiv H_2N-OH•HCl + DIEA	Molarity	LCAP[a]
1	wet DMF[b]	10	0.1	65
2	**dry DMF[c]**	10	0.1	69
3	**dry DMF, dry DIEA[d]**	10	0.1	71
5	wet DMF	**50**	0.1	82
6	wet DMF	50	**0.2**	85
7	wet DMF	50	**0.4**	78
8	wet DMF	50	**0.8**	72
9	wet DMF	**25**	0.2	83
10	wet DMF	**75**	0.2	79
11	wet DMF	**100**	0.2	0
12	wet DMF	**35**	0.2	82

[a]Liquid chromatography area percentage of **23.**
[b]Reagent-grade DMF used directly from the bottle without further drying.
[c]Reagent-grade DMF dried over 4Å molecular sieves.
[d]Reagent-grade DIEA dried over 4Å molecular sieves.

Figure S-11 Optimization of the Aminolysis

Entry	Water content[a]	Molarity	Temperature (˚C)	Solvent	LCAP[b]
1	3 equiv H_2O	0.0825	60	THF/ MeCN	49
2	**none[c]**	0.0825	60	THF/ MeCN	44
3	**wet solvent[d]**	0.0825	60	THF/ MeCN	50
4	**30 equiv H_2O**	0.0825	60	THF/ MeCN	56
5	**wet solvent + 30 equiv H_2O**	0.0825	60	THF/ MeCN	56
6	**wet solvent + 60 equiv H_2O**	0.0825	60	THF/ MeCN	64
7	**wet solvent + 120 equiv H_2O**	0.0825	60	THF/ MeCN	60
8	**wet solvent + 60 equiv H_2O**	**0.04**	60	THF/ MeCN	55
9	wet solvent + 60 equiv H_2O	**0.17**	60	THF/ MeCN	44
10	wet solvent + 60 equiv H_2O	**0.0825**	**50**	THF/ MeCN	44
11	wet solvent + 60 equiv H_2O	0.0825	**70**	THF/ MeCN	38
12	wet solvent + 60 equiv H_2O	0.0825	**60**	**MeCN**	59
13	wet solvent + 60 equiv H_2O	0.0825	60	**DMA/ MeCN**	52

[a]Description of the source of solvent, and whether or not additional H_2O was added.
[b]Liquid chromatography area percentage of **21**.
[c]THF and MeCN dried over 4Å molecular sieves.
[d]Reagent-grade THF and MeCN used directly from the bottle without further drying.

Figure S-12 Optimization of the Lössen Rearrangement

References

(1) Schäfer, D.; Weiß, P.; Ermert, J.; Castillo Meleán, J.; Zarrad, F.; Neumaier, B. Preparation of No-carrier-added 6-[18f]Fluoro-l-tryptophan via Cu-mediated Radiofluorination. *European Journal of Organic Chemistry* **2016**, *2016* (27), 4621–4628.

(2) Curreli, F.; Choudhury, S.; Pyatkin, I.; Zagorodnikov, V. P.; Bulay, A. K.; Altieri, A.; Kwon, Y. D.; Kwong, P. D.; Debnath, A. K. Design, Synthesis, and Antiviral Activity of Entry Inhibitors That Target the CD4-Binding Site of HIV-1. *Journal of Medicinal Chemistry* **2012**, *55* (10), 4764–4775.

(3) Fritschi, C. J.; Anang, S.; Gong, Z.; Mohammadi, M.; Richard, J.; Bourassa, C.; Severino, K. T.; Richter, H.; Yang, D.; Chen, H.-C.; et al. Indoline CD4-Mimetic Compounds Mediate Potent and Broad HIV-1 Inhibition and Sensitization to Antibody-Dependent Cellular Cytotoxicity. *Proc. Natl. Acad. Sci.* **2023**, *120* (13).

5. ^{1}H-NMR and ^{13}C-NMR spectra

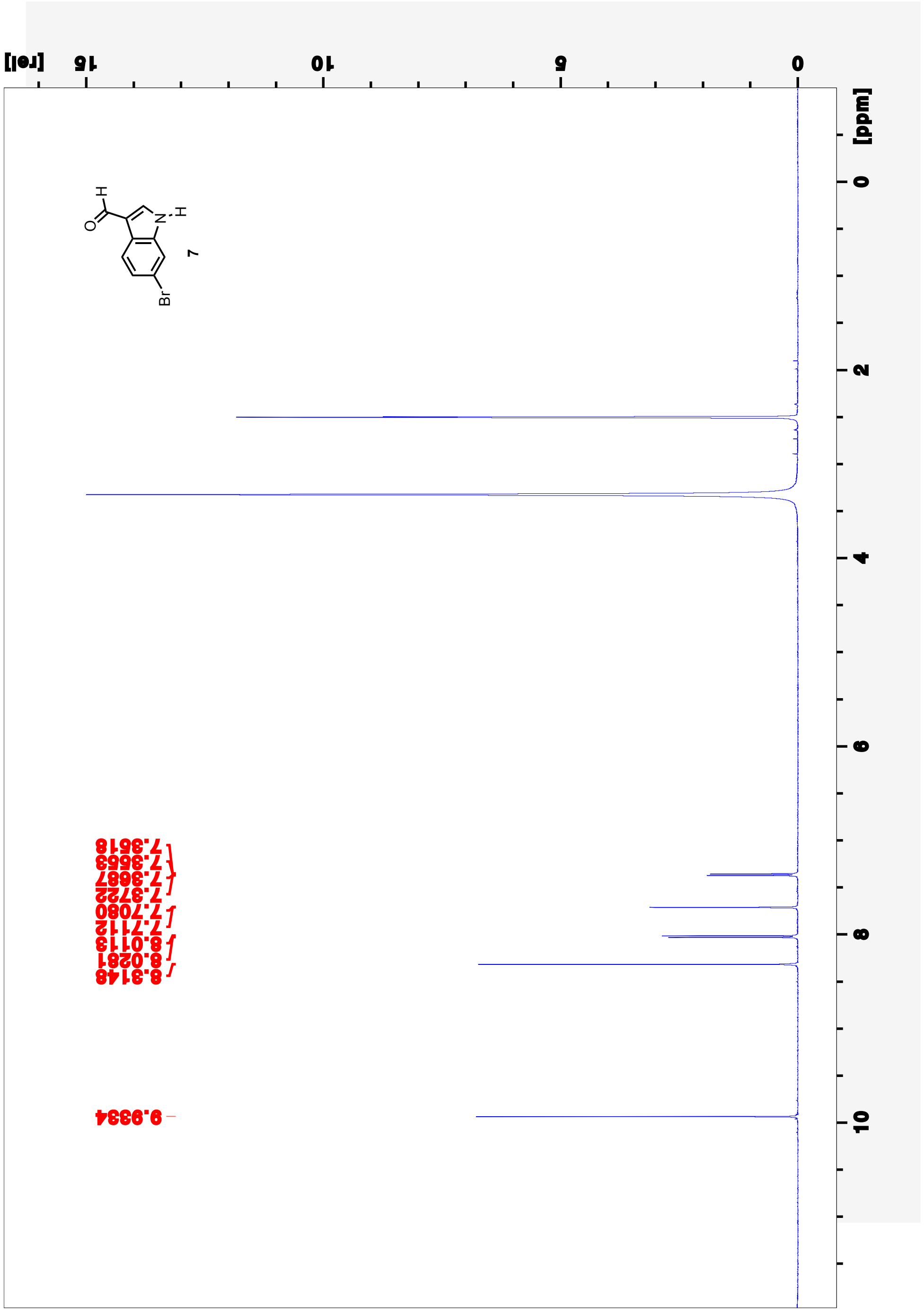

Figure A.2.1 The 500 MHz ^{1}H NMR Spectrum of compound **7**

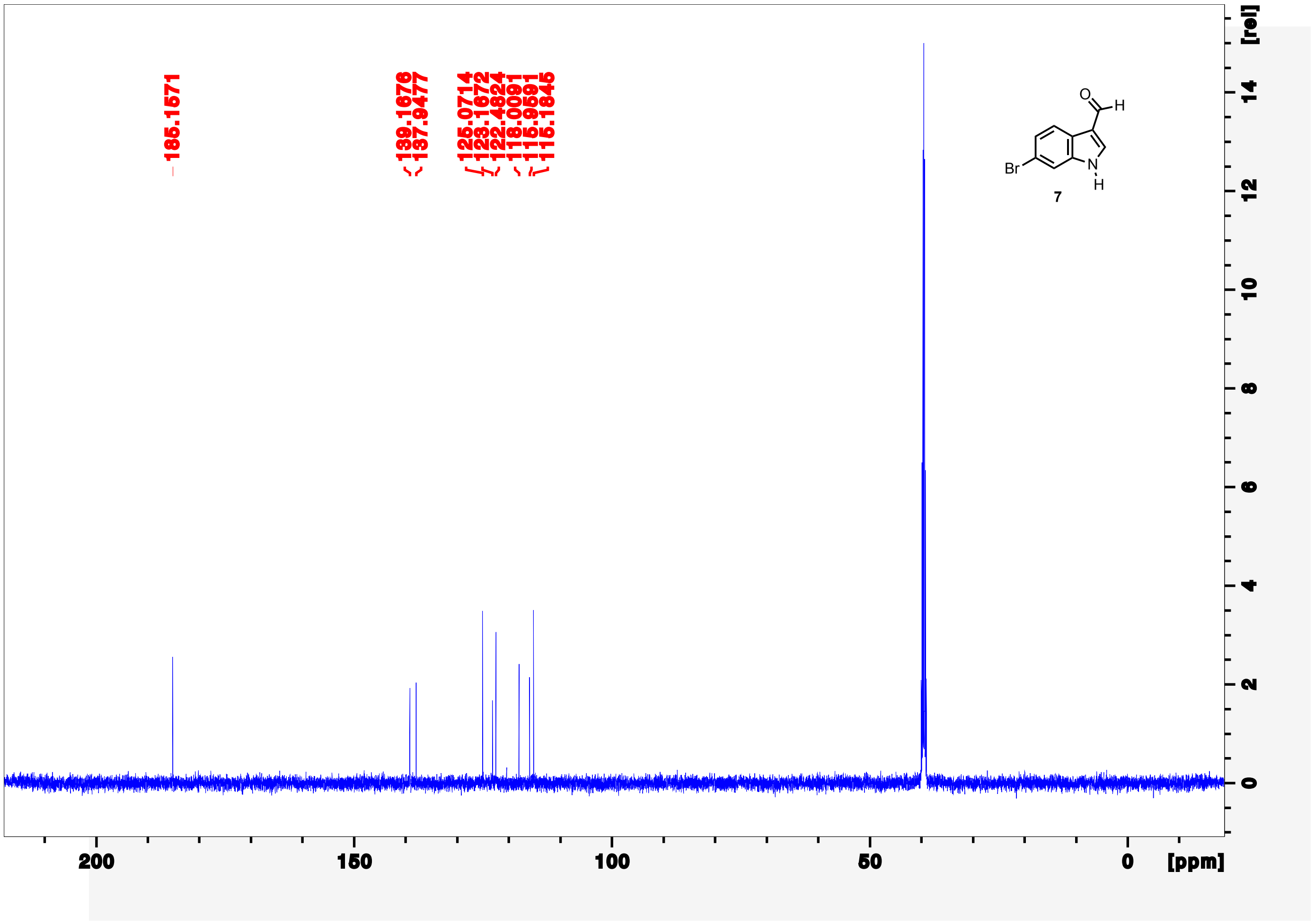

Figure A.2.2 The 125 MHz ^{13}C NMR Spectrum of compound **7**

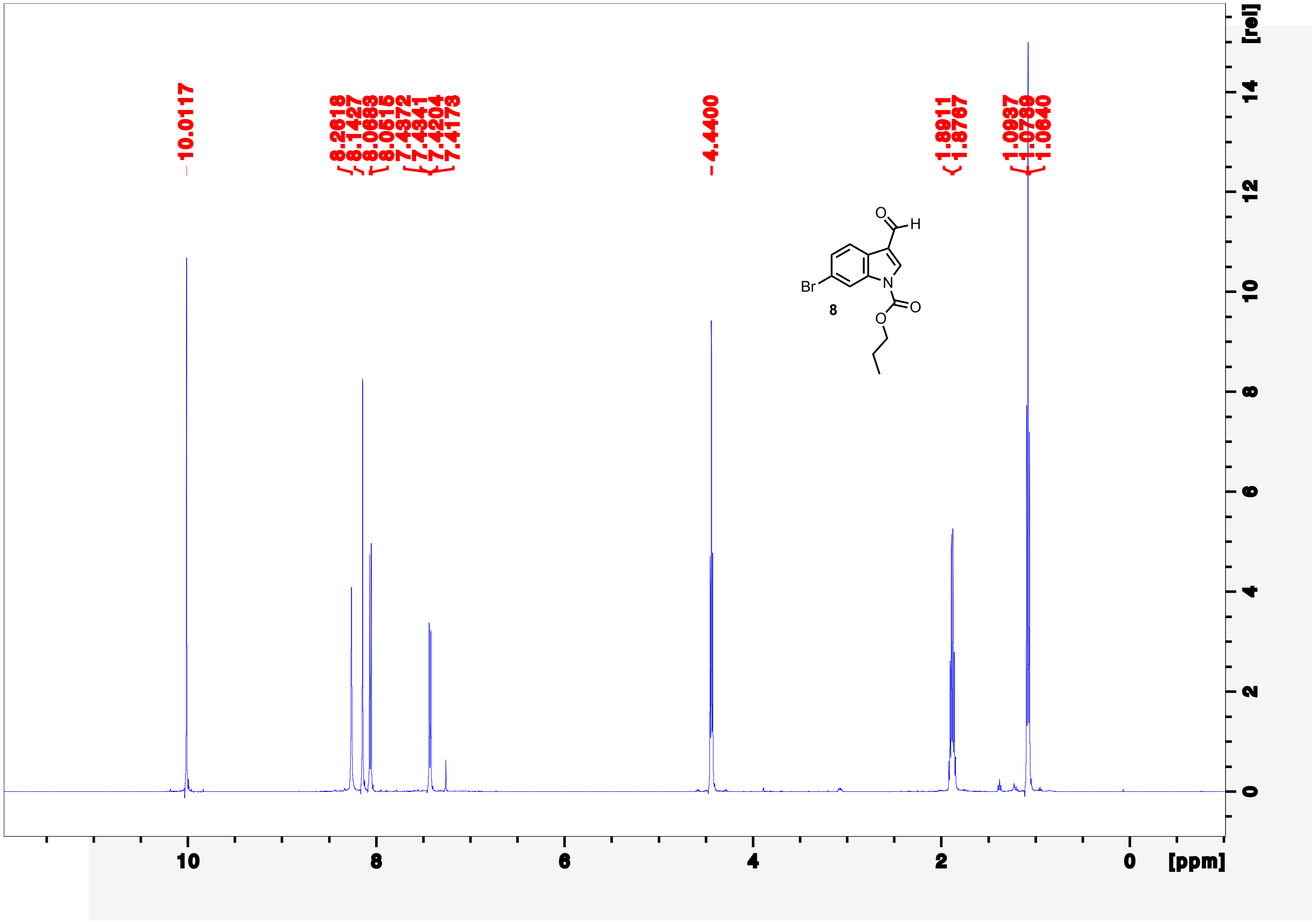

Figure A.2.3 The 500 MHz ^{1}H NMR Spectrum of compound **8**

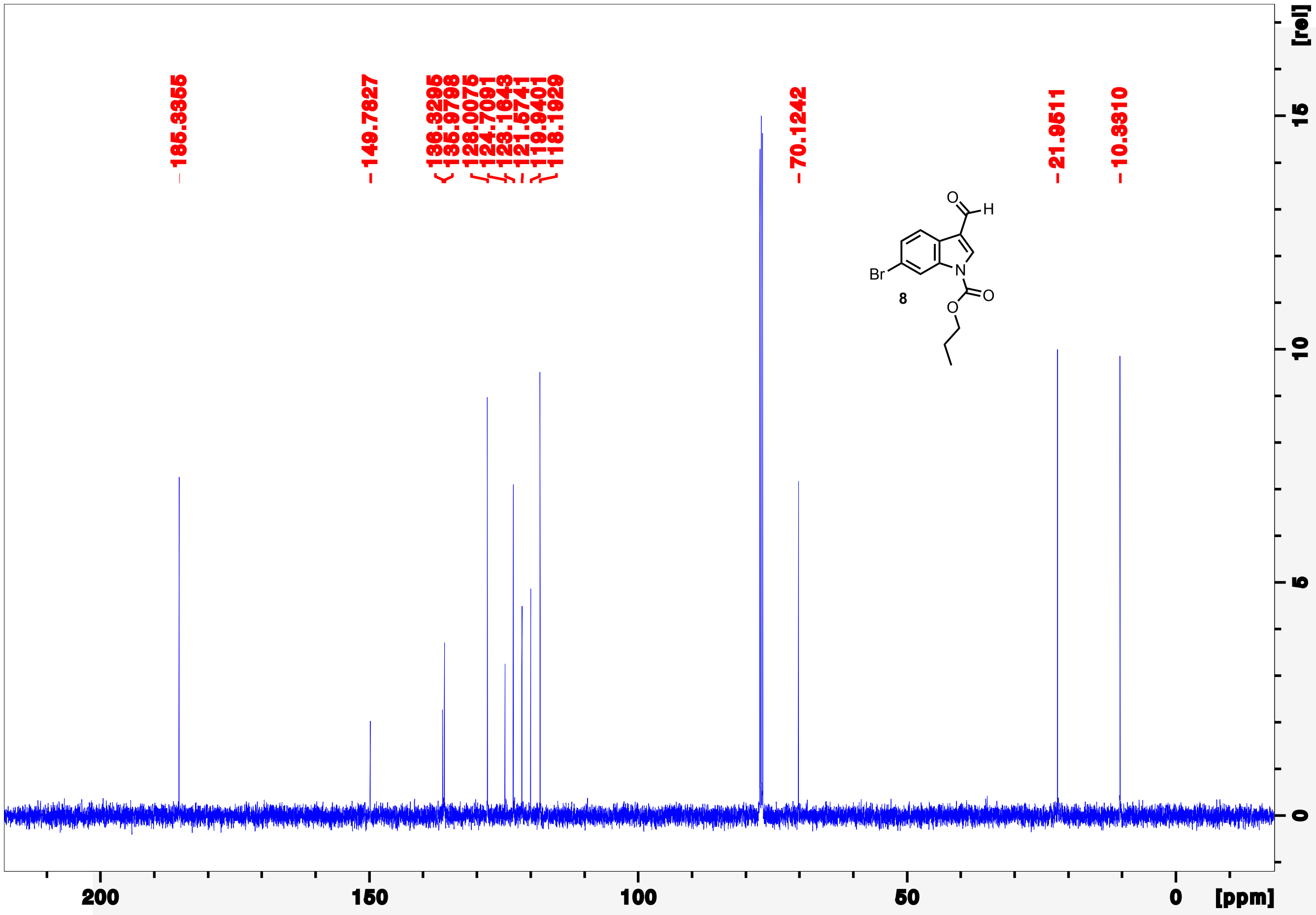

Figure A.2.4 The 125 MHz ^{13}C NMR Spectrum of compound **8**

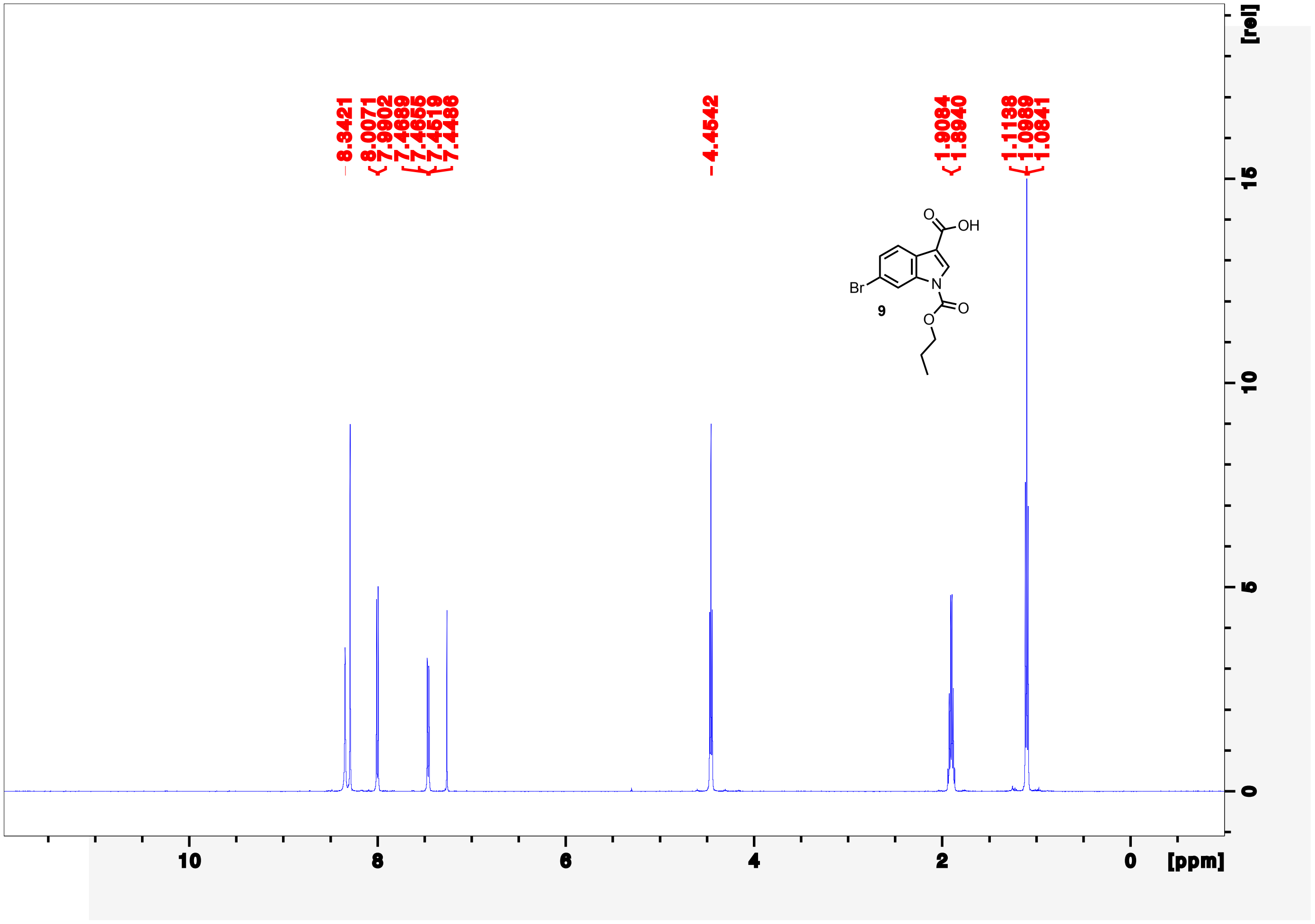

Figure A.2.5 The 500 MHz ^{1}H NMR Spectrum of compound **9**

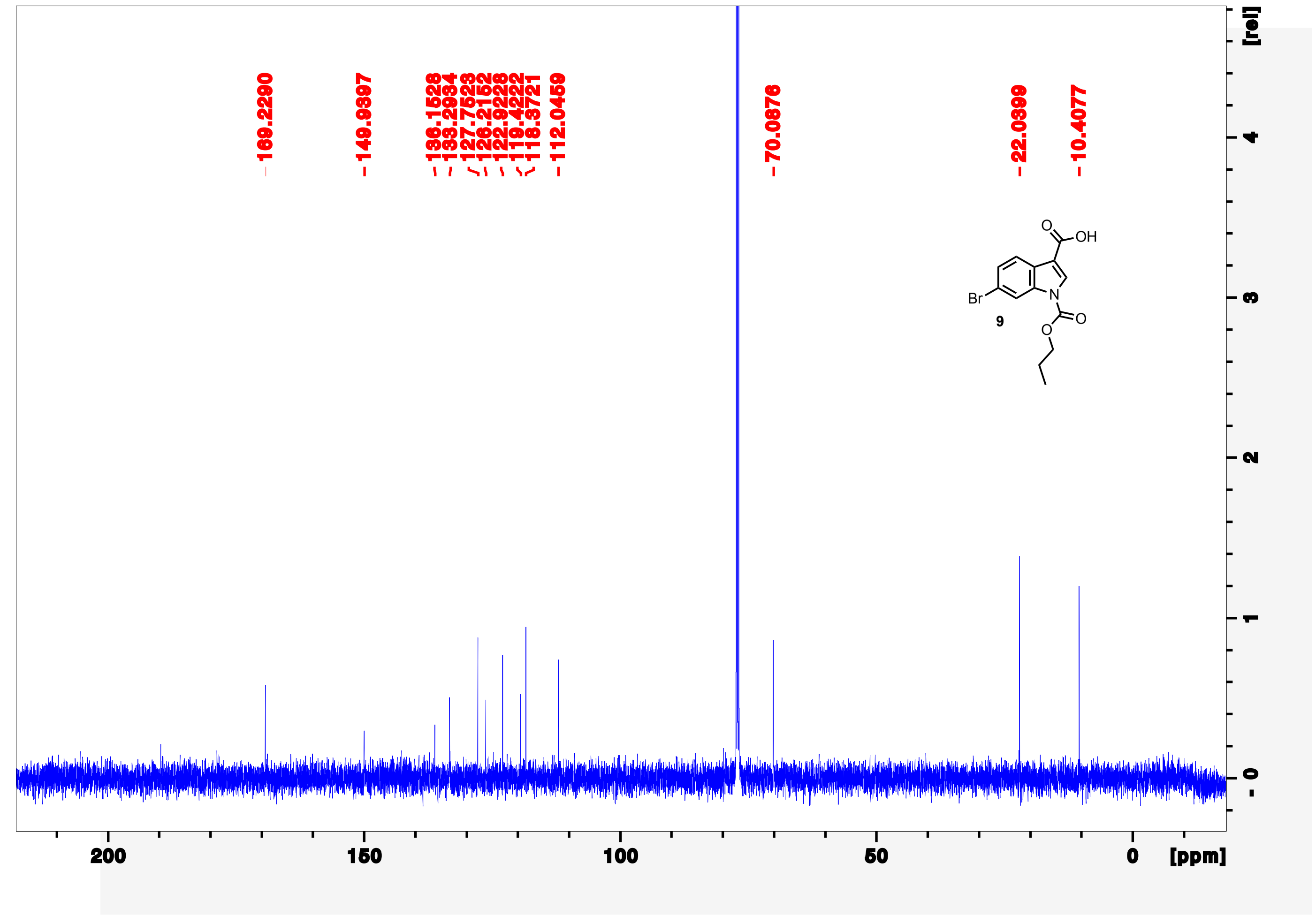

Figure A.2.6 The 125 MHz ^{13}C NMR Spectrum of compound 9

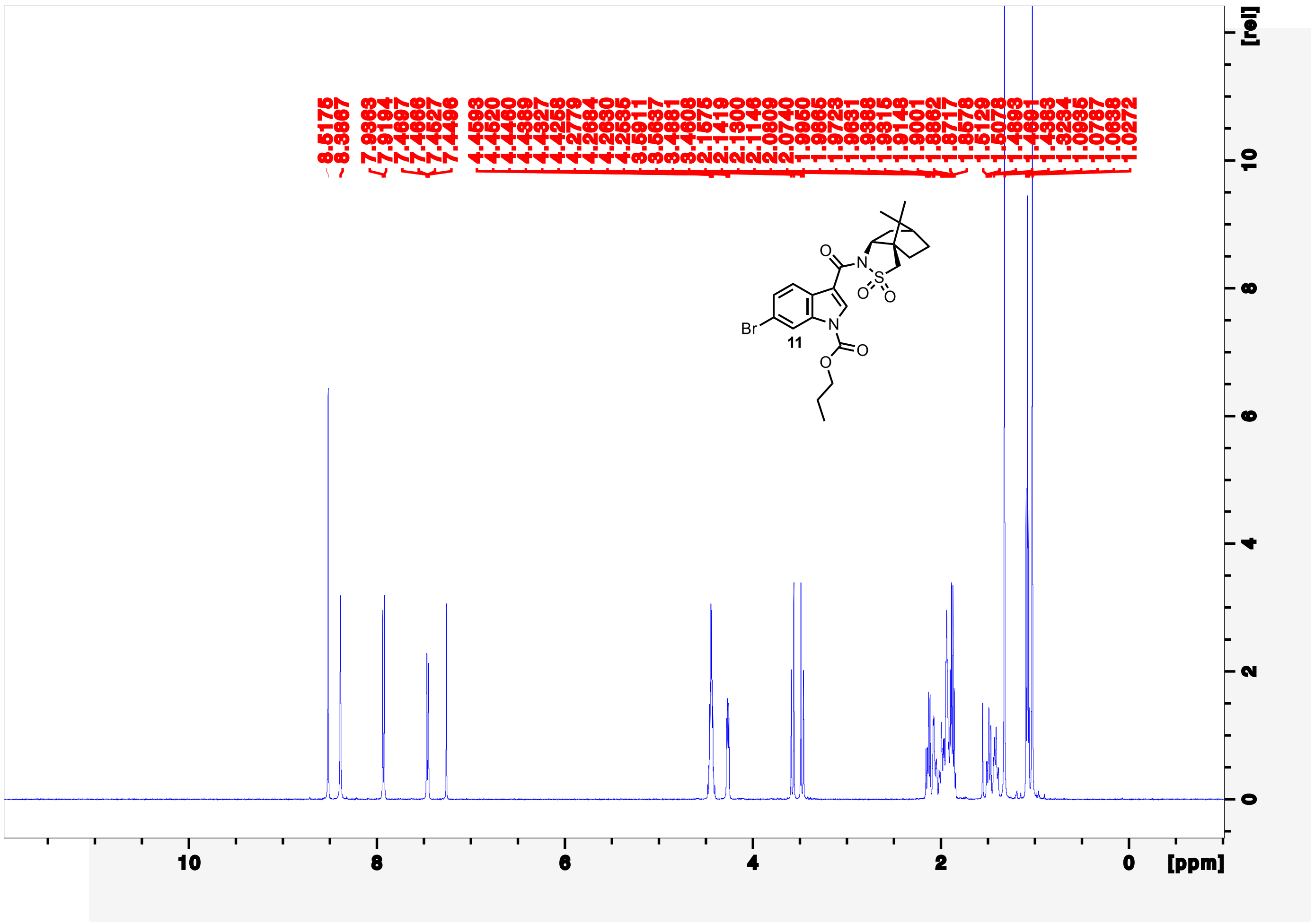

Figure A.2.7 The 500 MHz ^{1}H NMR Spectrum of compound **11**

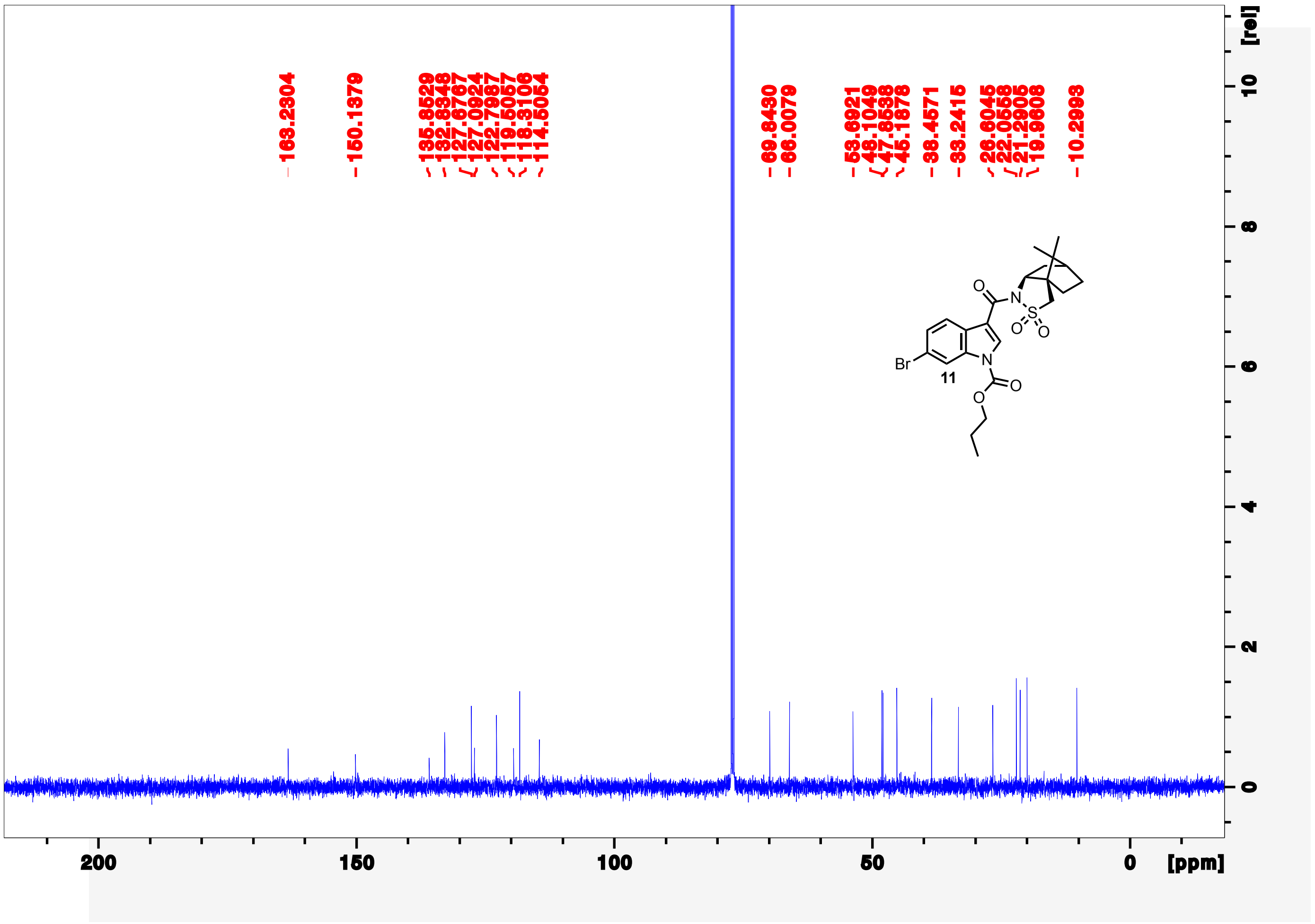

Figure A.2.8 The 125 MHz ^{13}C NMR Spectrum of compound **11**

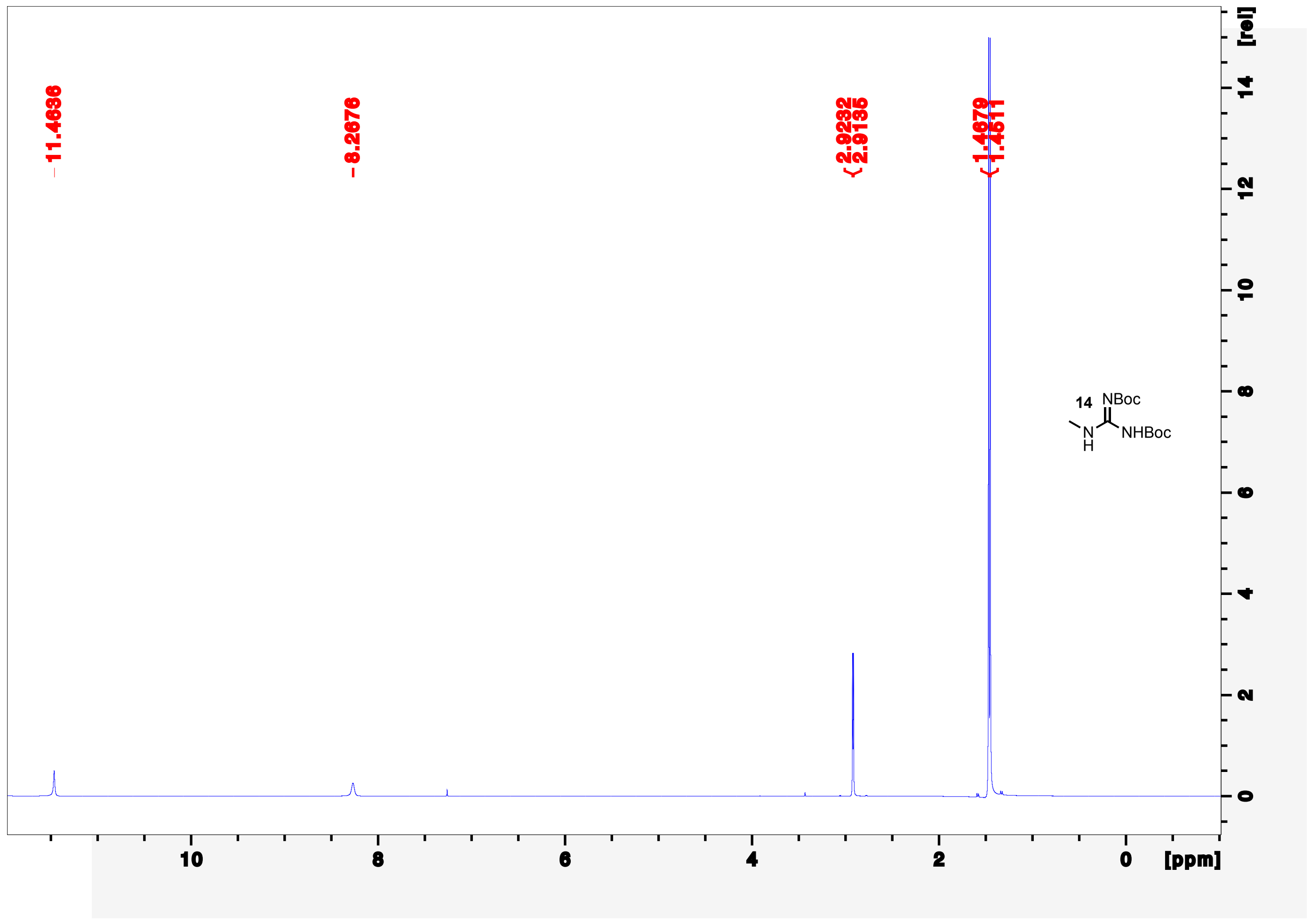

Figure A.2.9 The 500 MHz ^{1}H NMR Spectrum of compound **14**

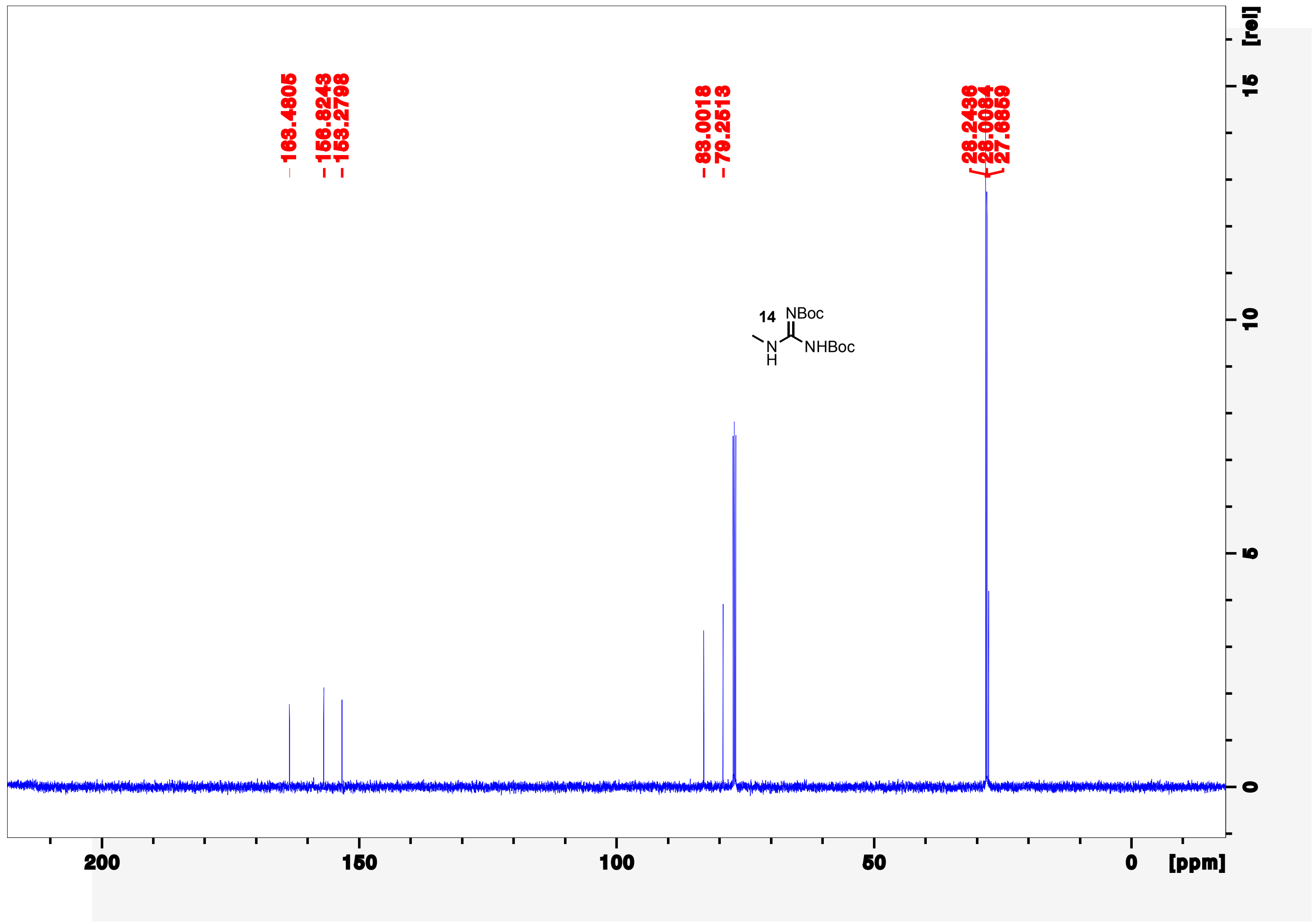

Figure A.2.10 The 125 MHz ^{13}C NMR Spectrum of compound **14**

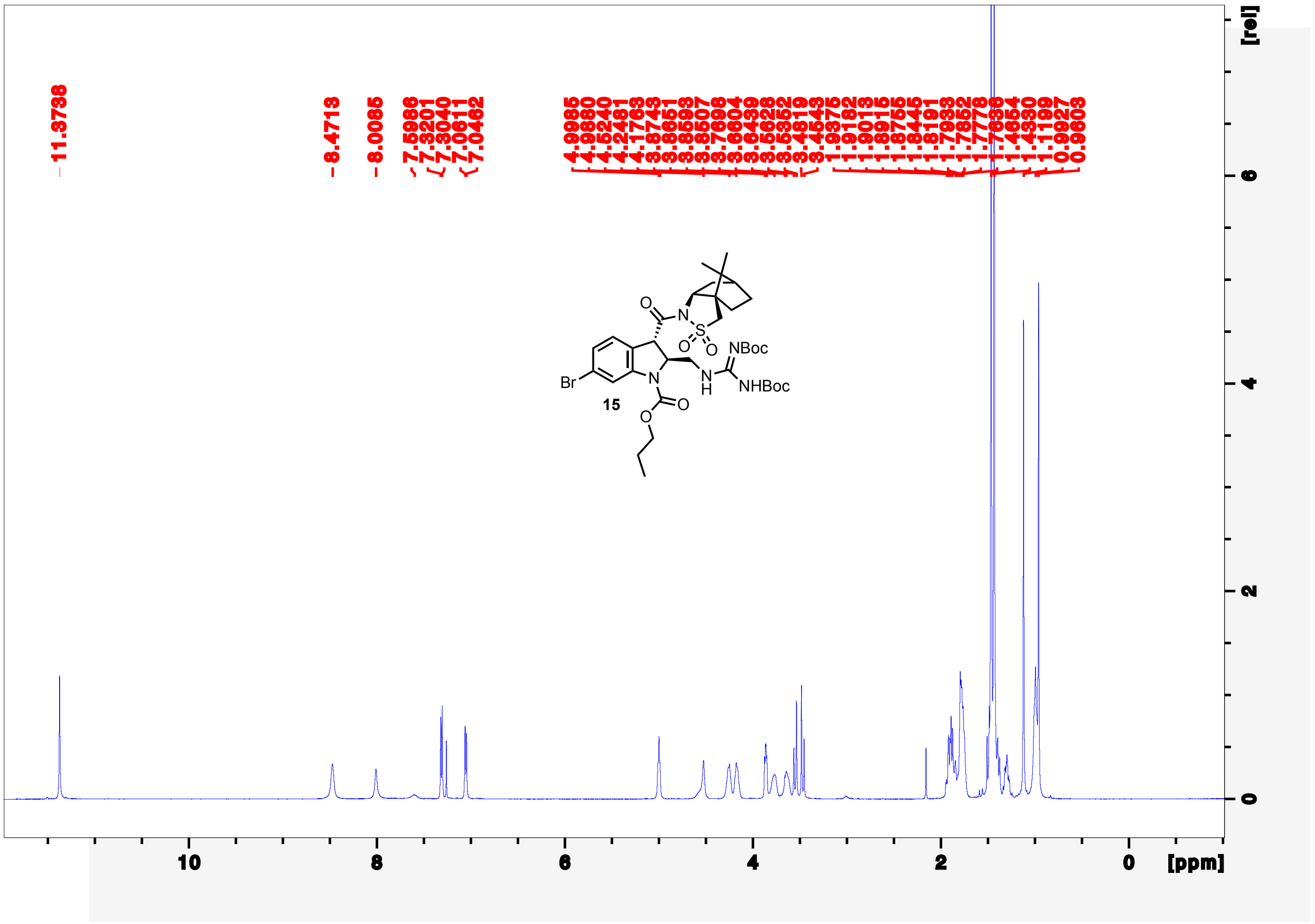

Figure A.2.11 The 500 MHz ^{1}H NMR Spectrum of compound **15**

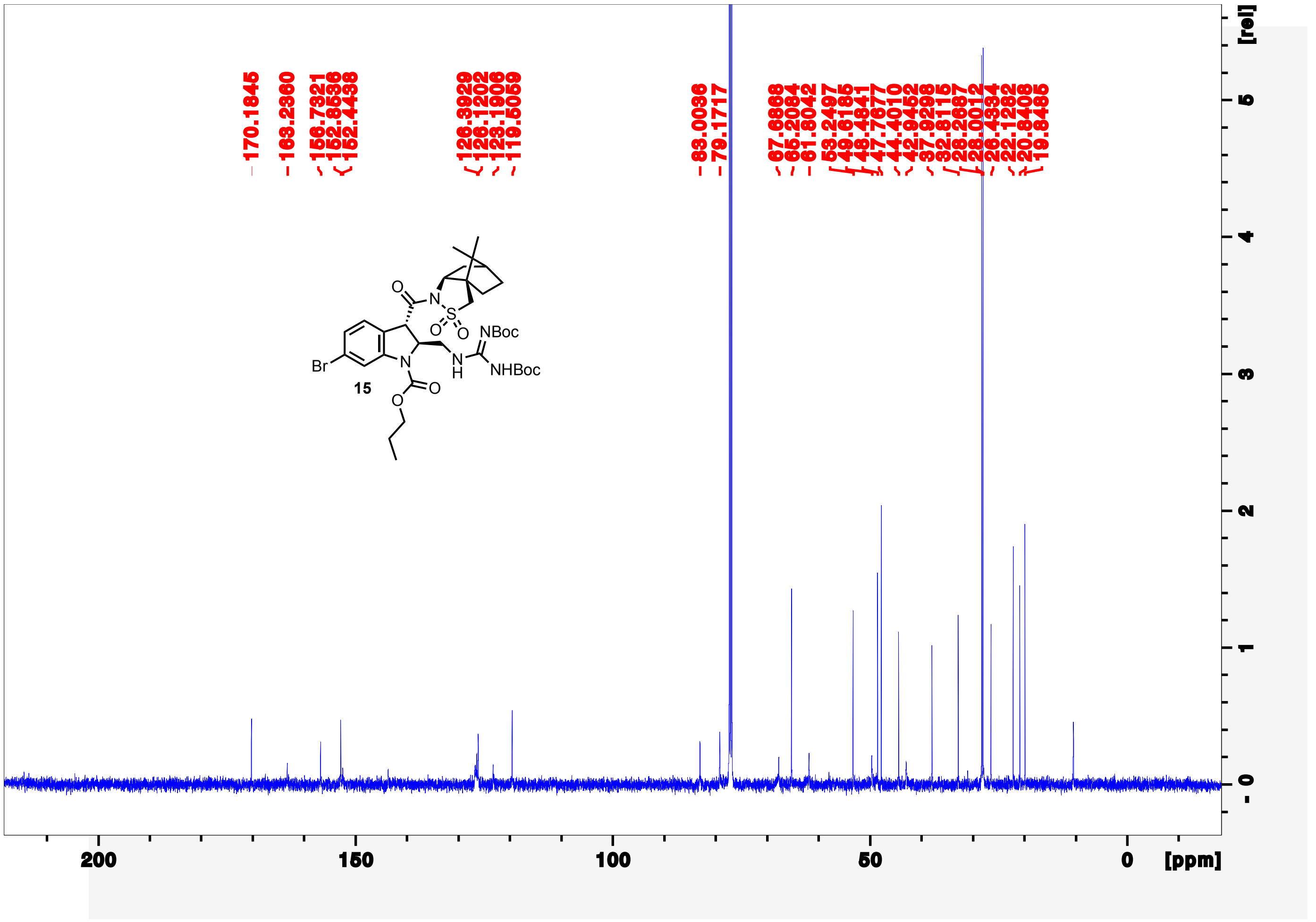

Figure A.2.12 The 125 MHz ^{13}C NMR Spectrum of compound **15**

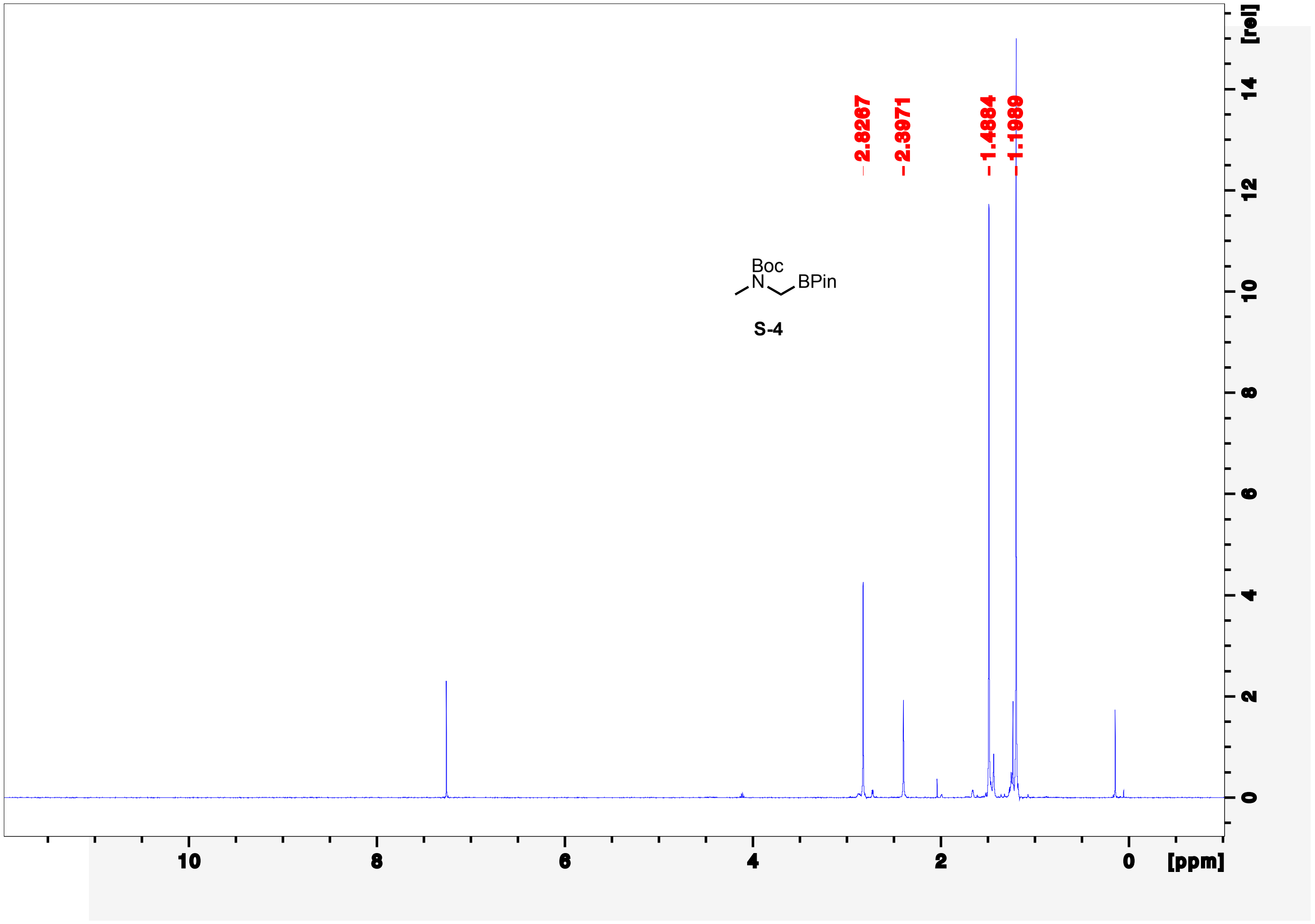

Figure A.2.13 The 500 MHz ^{1}H NMR Spectrum of compound **S-4**

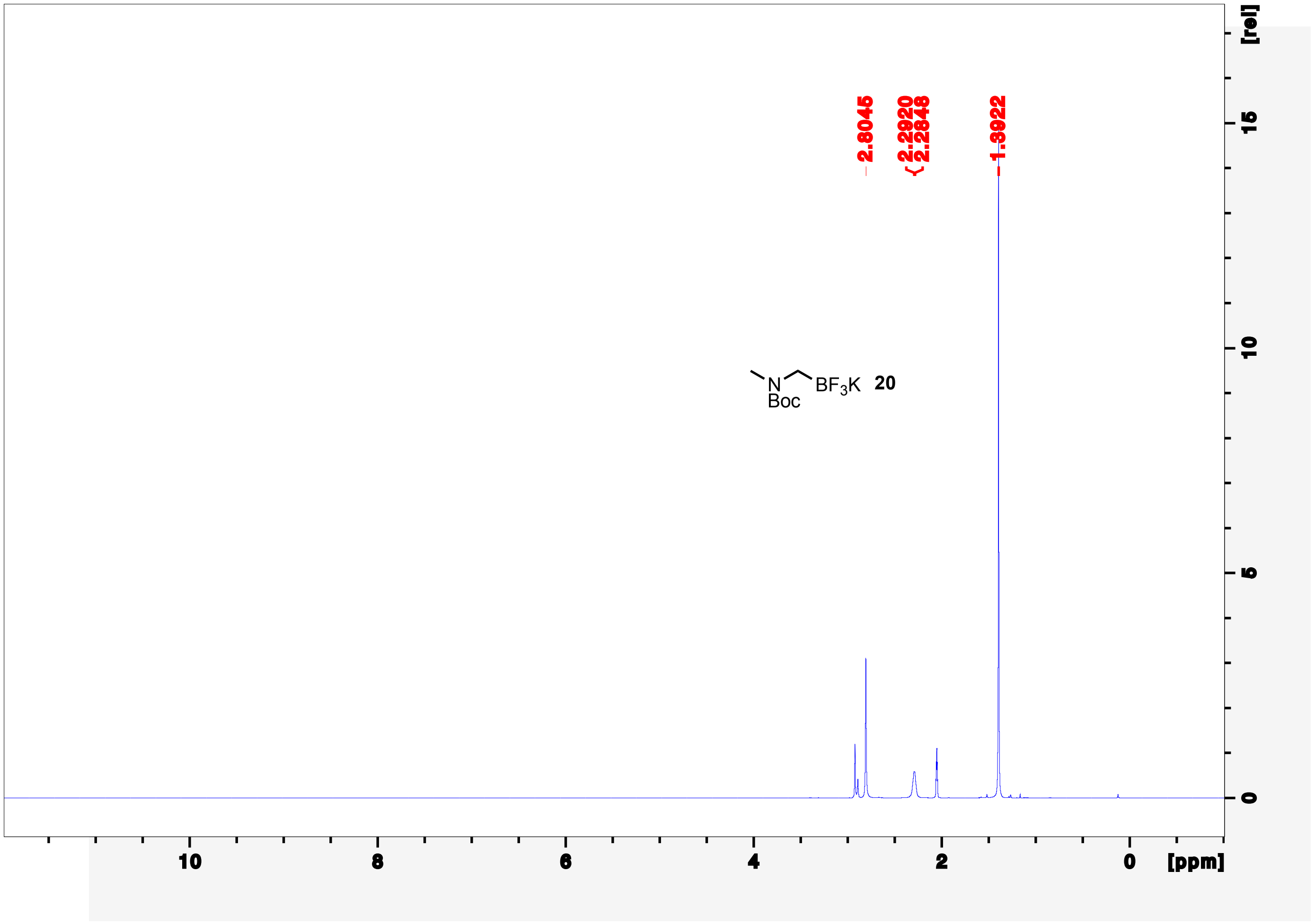

Figure A.2.14 The 500 MHz ^{1}H NMR Spectrum of compound **20**

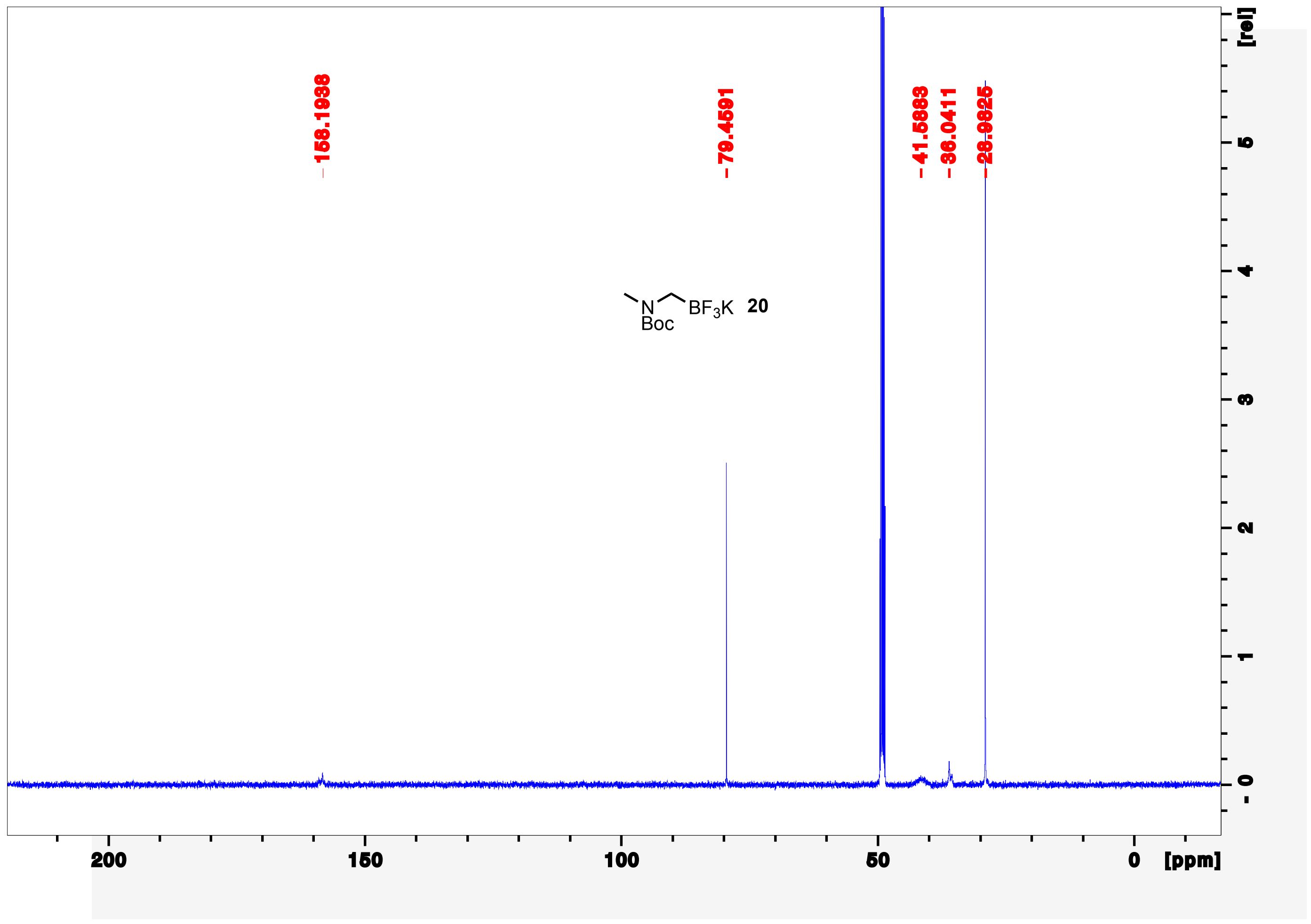

Figure A.2.15 The 125 MHz ^{13}C NMR Spectrum of compound **20**

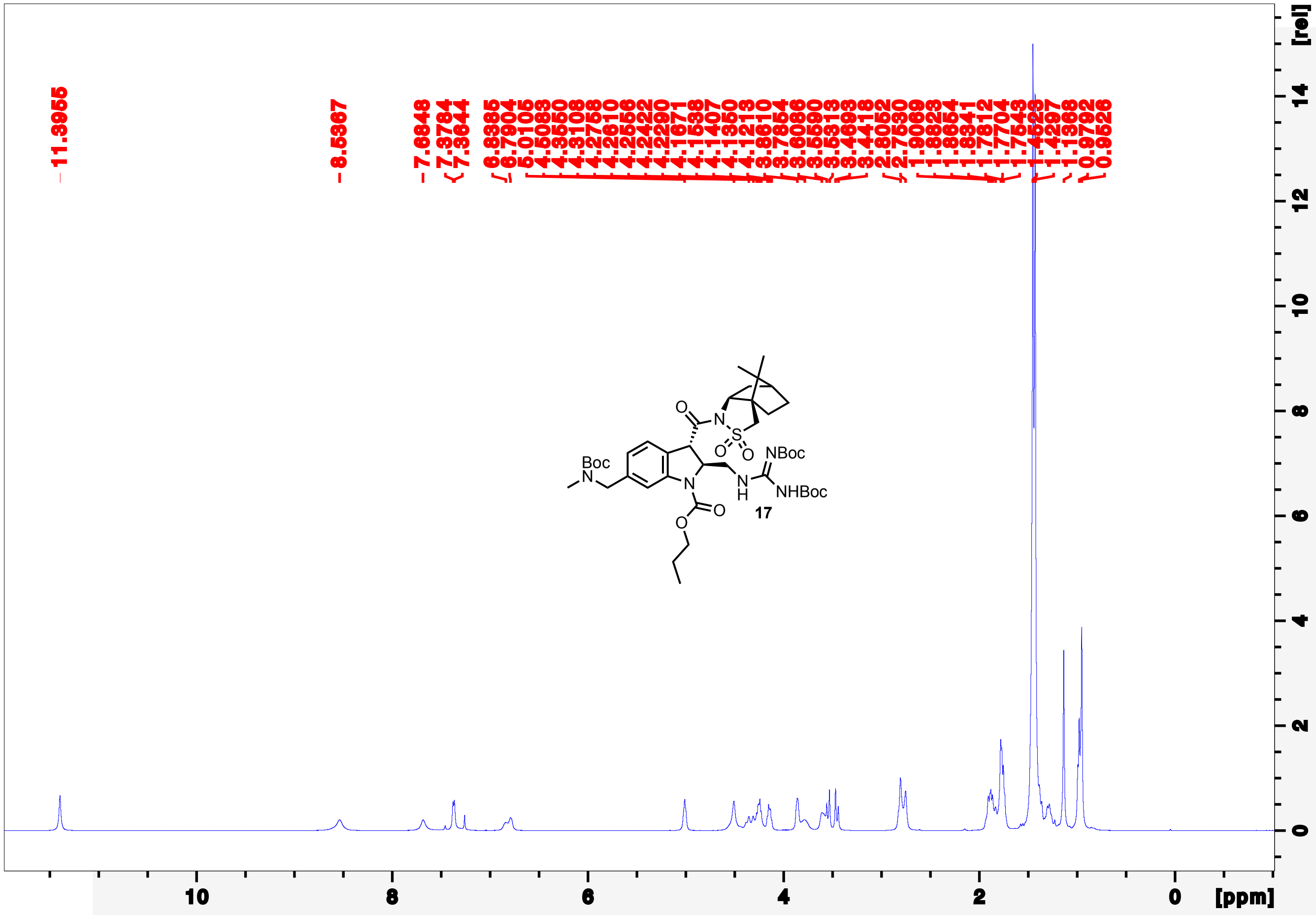

Figure A.2.16 The 500 MHz ^{1}H NMR Spectrum of compound **17** at 24.85˚C

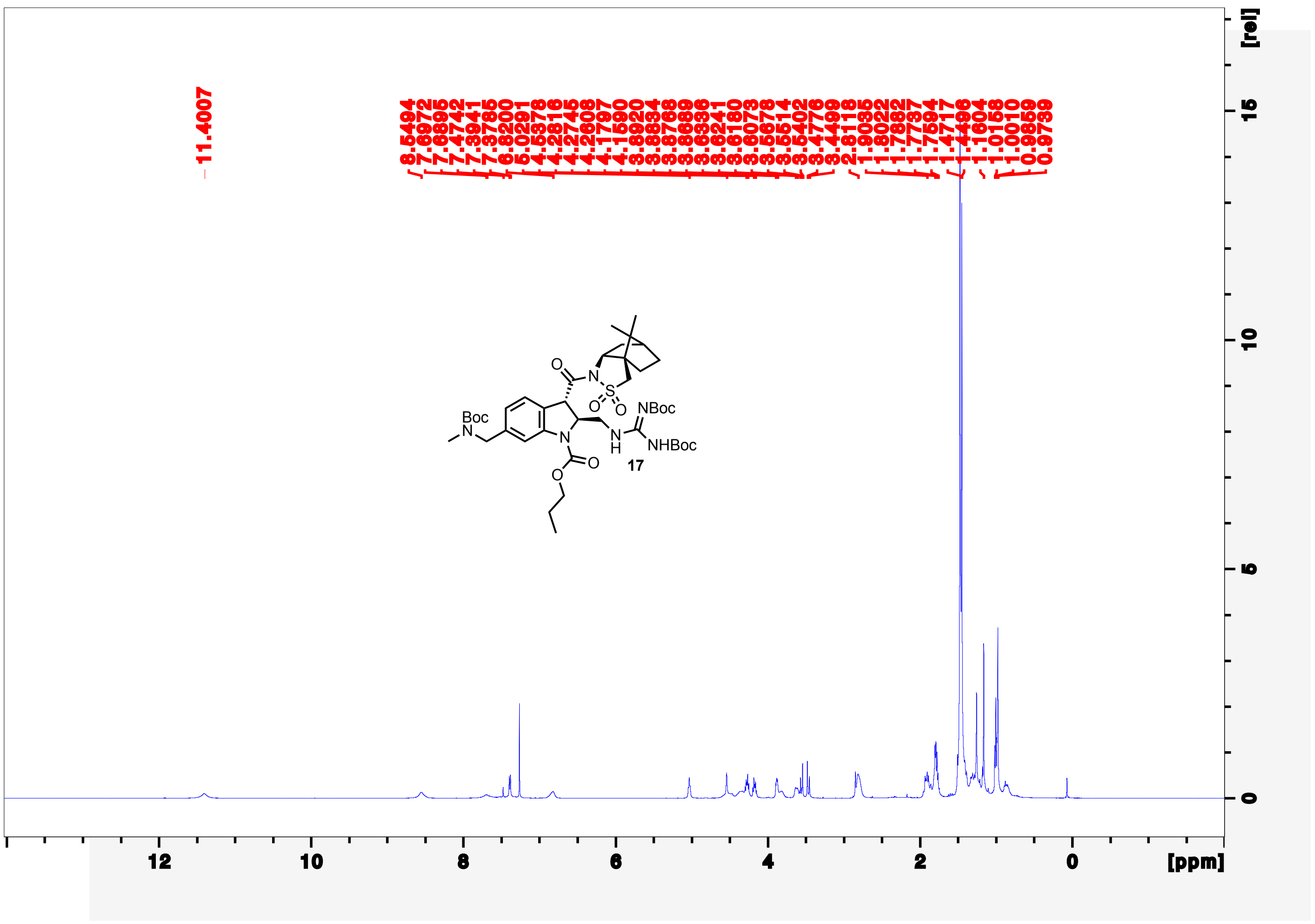

Figure A.2.17 The 500 MHz ^{1}H NMR Spectrum of compound **17** at 34.85˚C

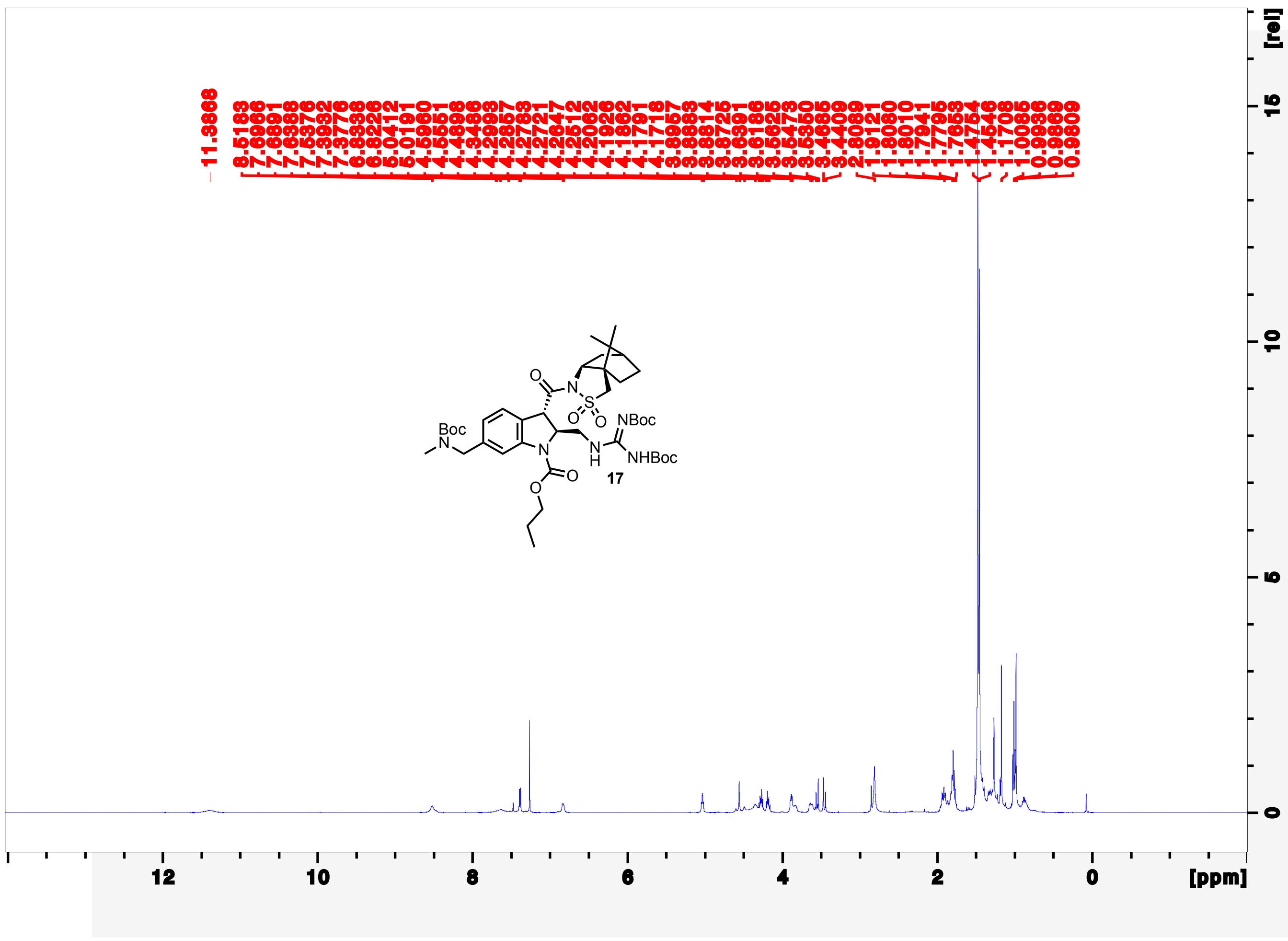

Figure A.2.18 The 500 MHz ^{1}H NMR Spectrum of compound **17** at 50˚C

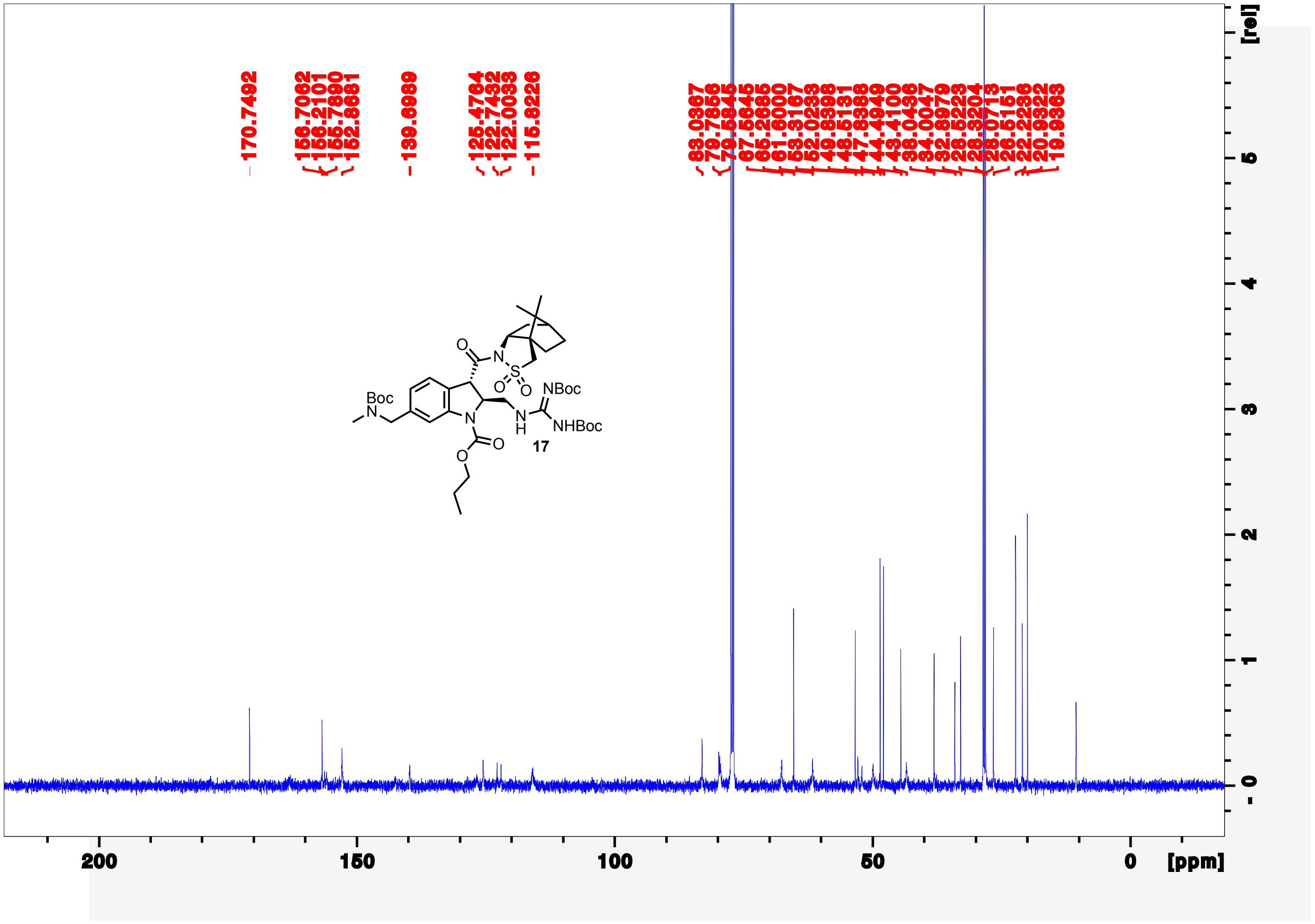

Figure A.2.19 The 125 MHz ^{13}C NMR Spectrum of compound **17** at 24.85˚C

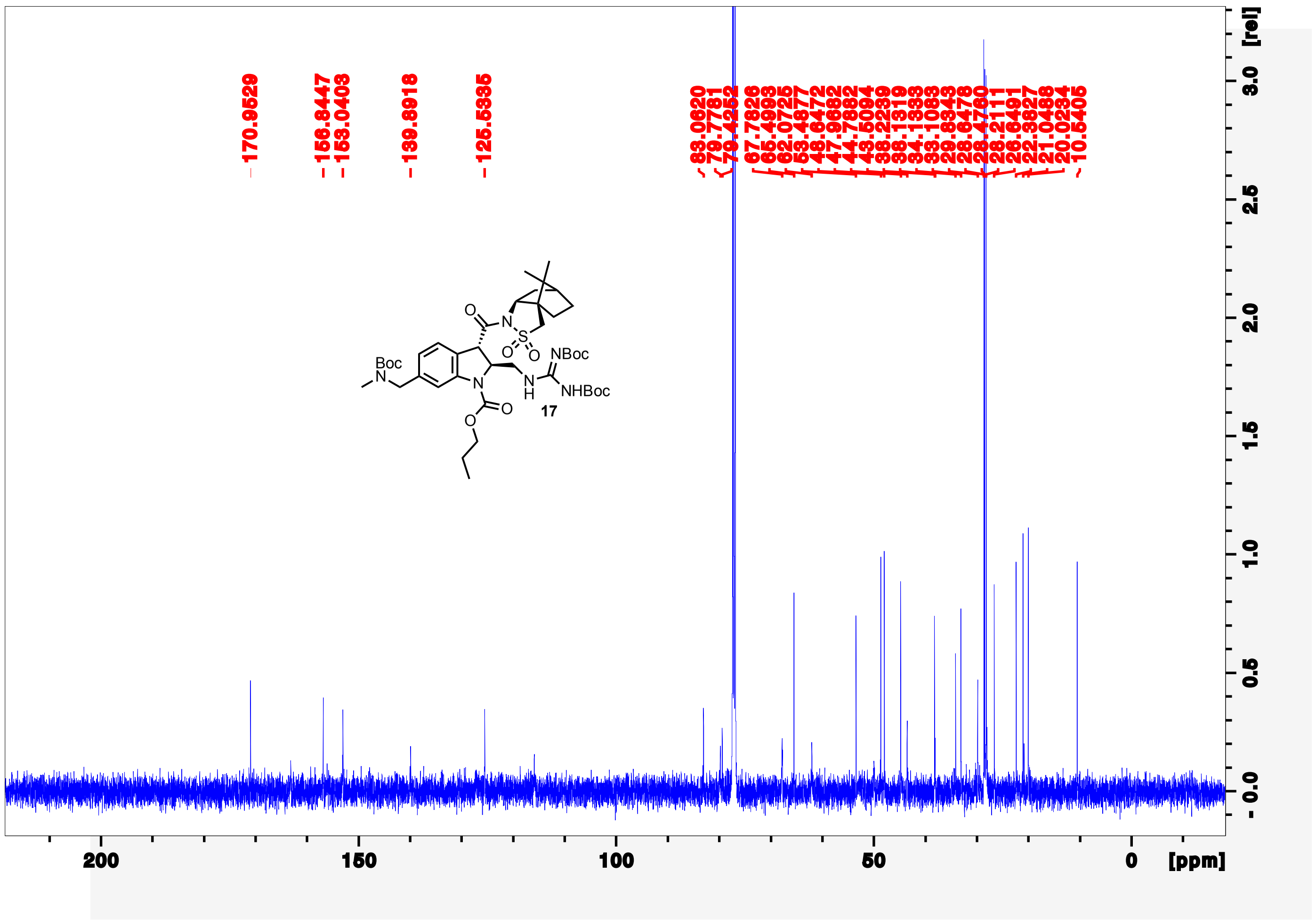

Figure A.2.20 The 125 MHz ^{13}C NMR Spectrum of compound **17** at 50˚C

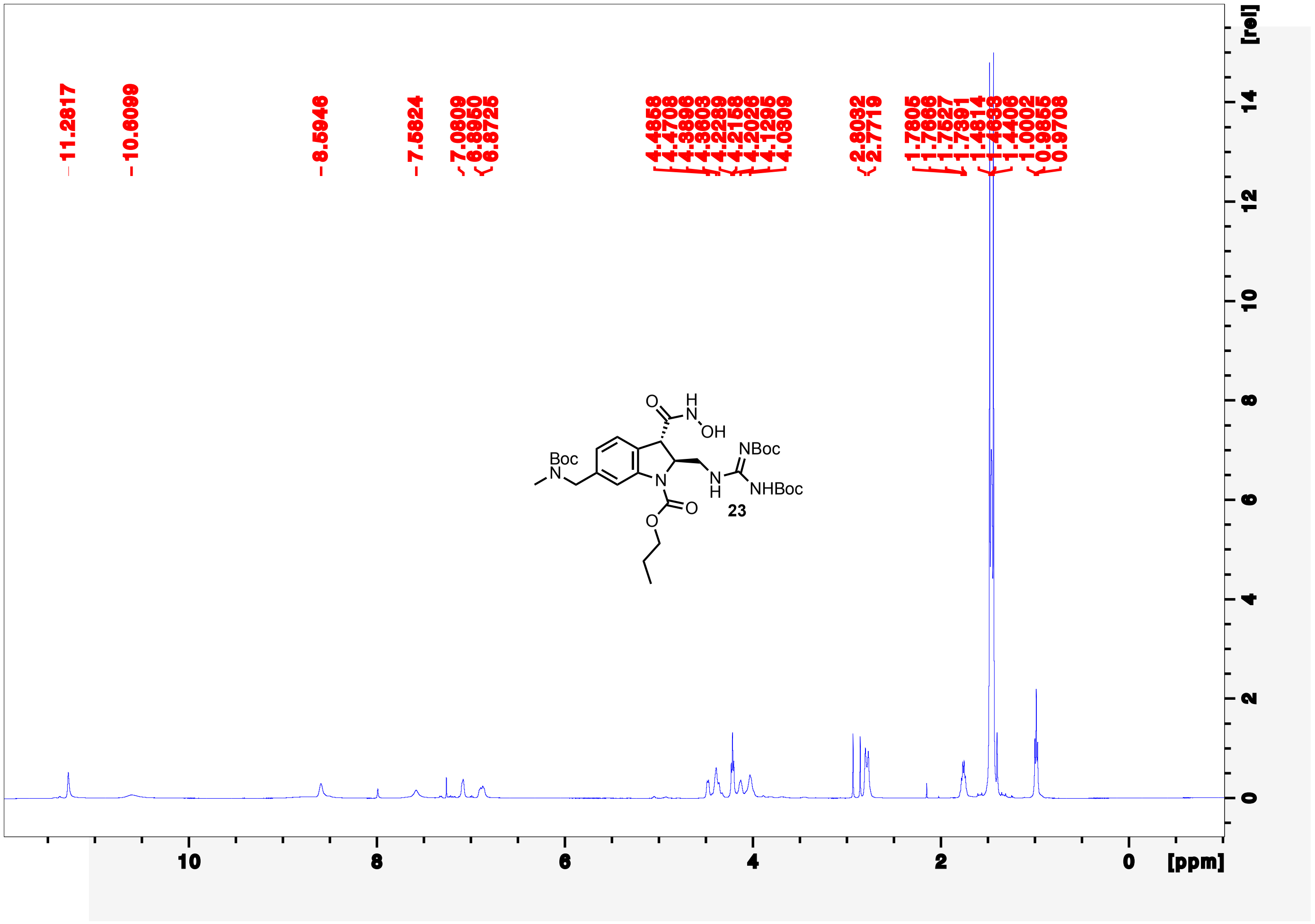

Figure A.2.21 The 500 MHz ^{1}H NMR Spectrum of compound **23**

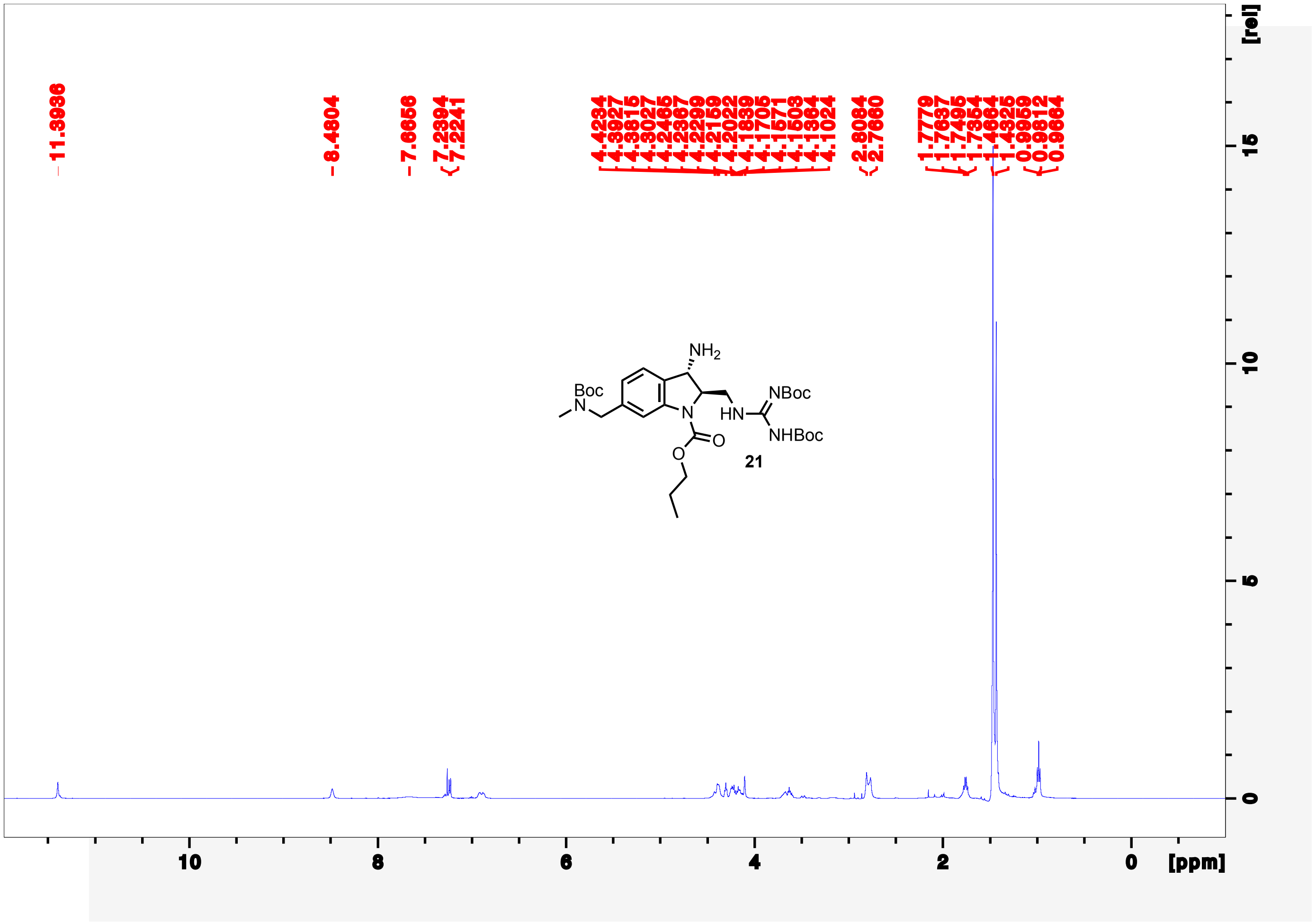

Figure A.2.22 The 500 MHz ^{1}H NMR Spectrum of compound **21**

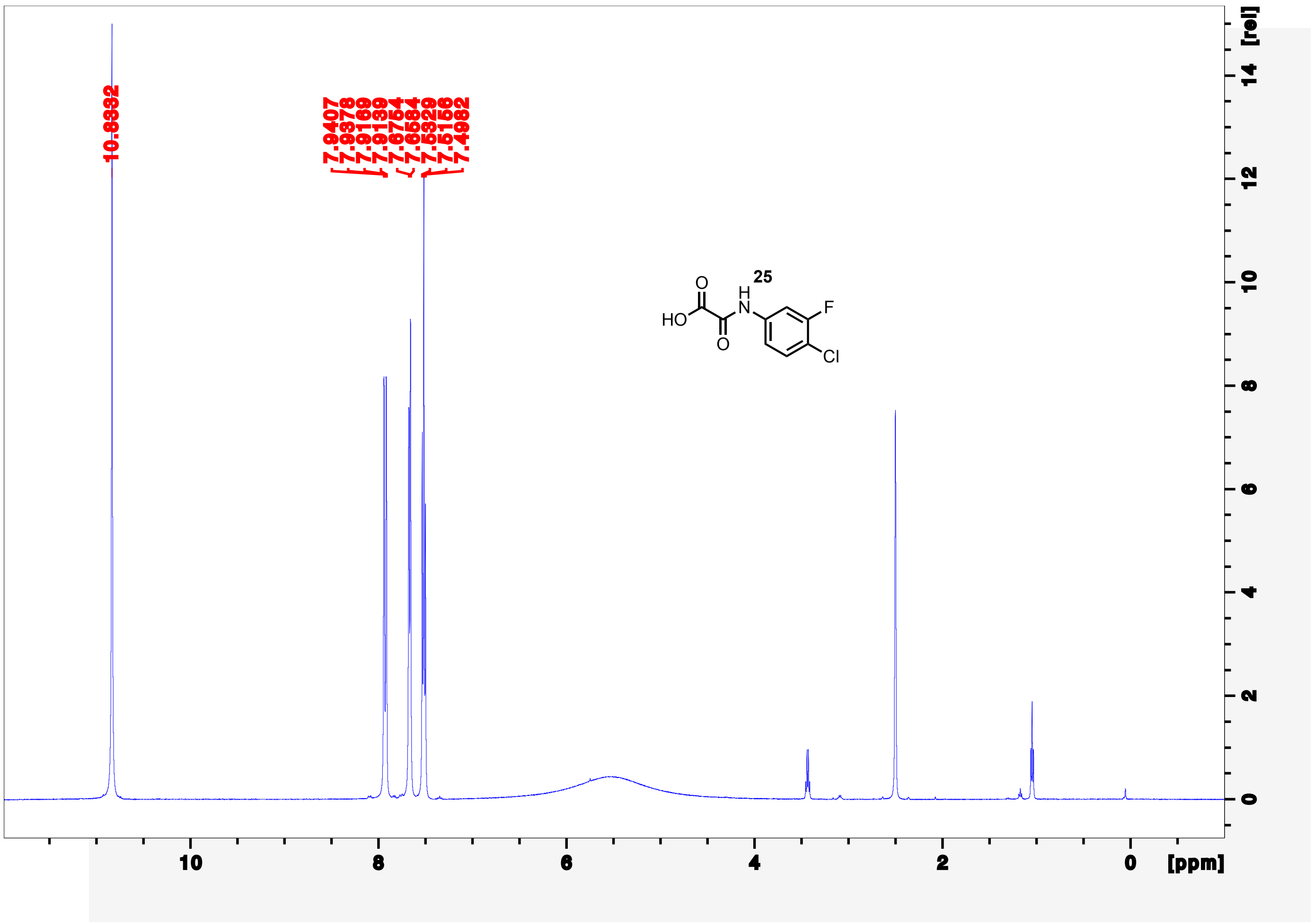

Figure A.2.23 The 500 MHz ^{1}H NMR Spectrum of compound **25**

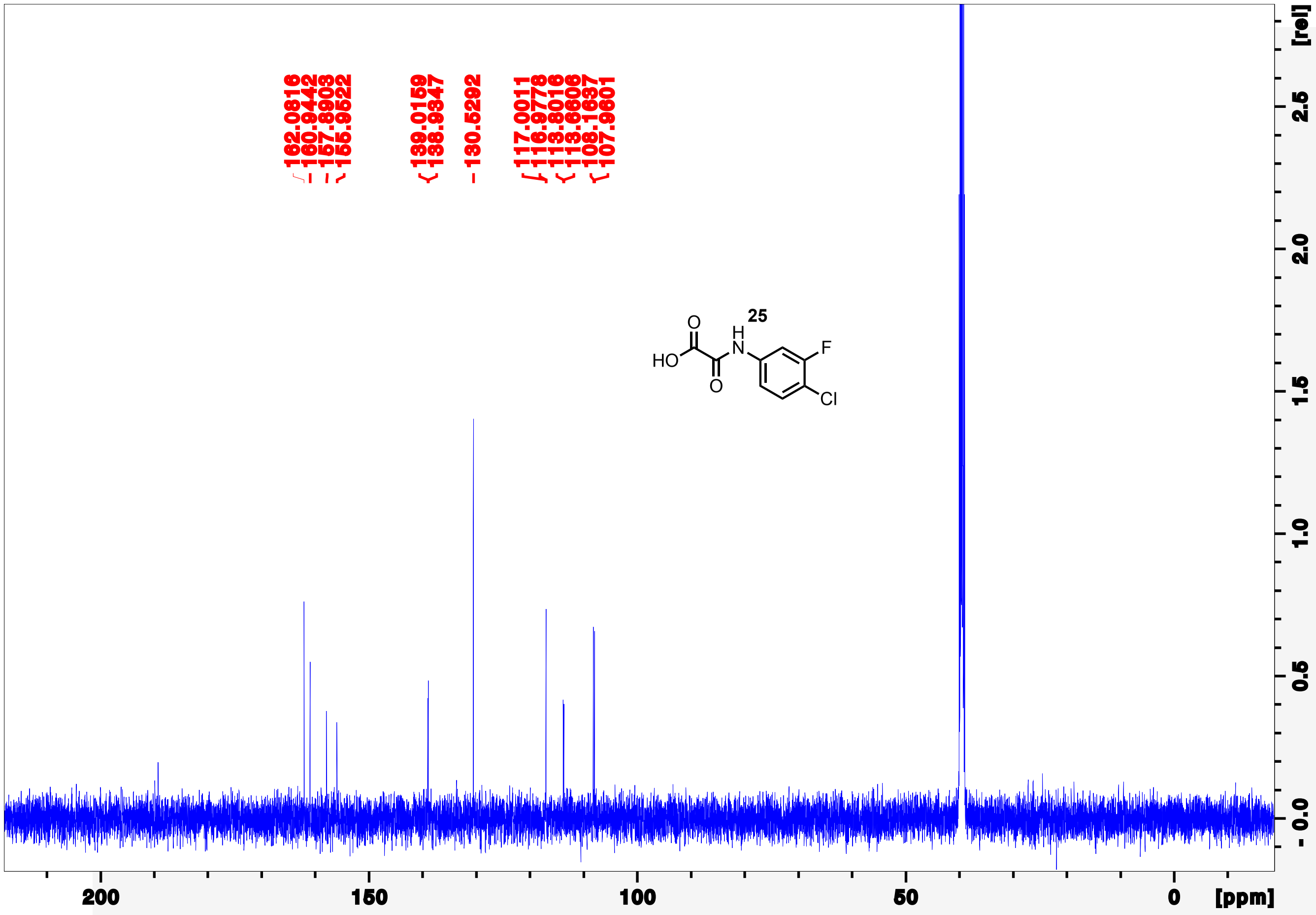

Figure A.2.24 The 125 MHz ^{13}C NMR Spectrum of compound **25**

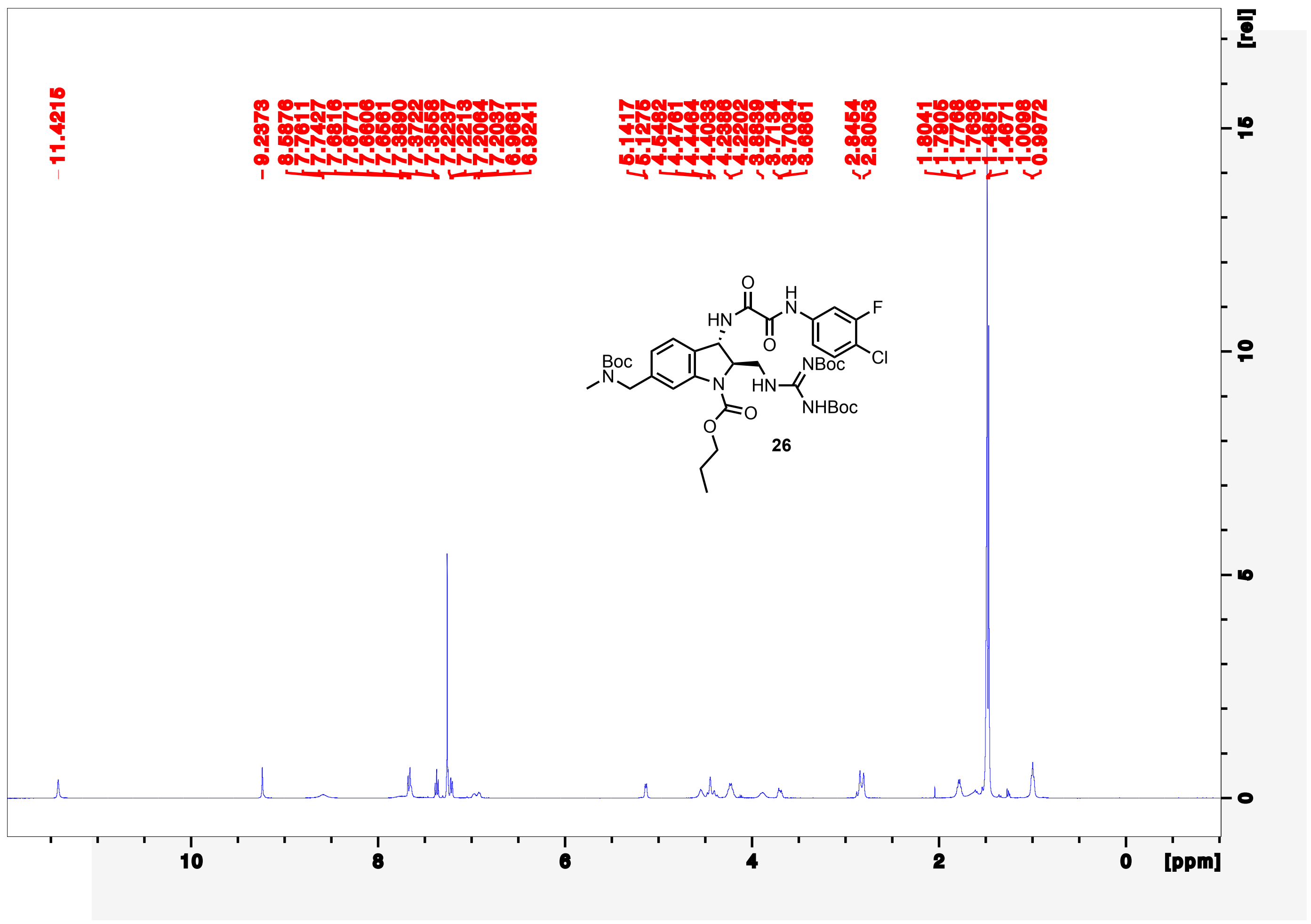

Figure A.2.25 The 500 MHz ^{1}H NMR Spectrum of compound **26** at 26.85˚C

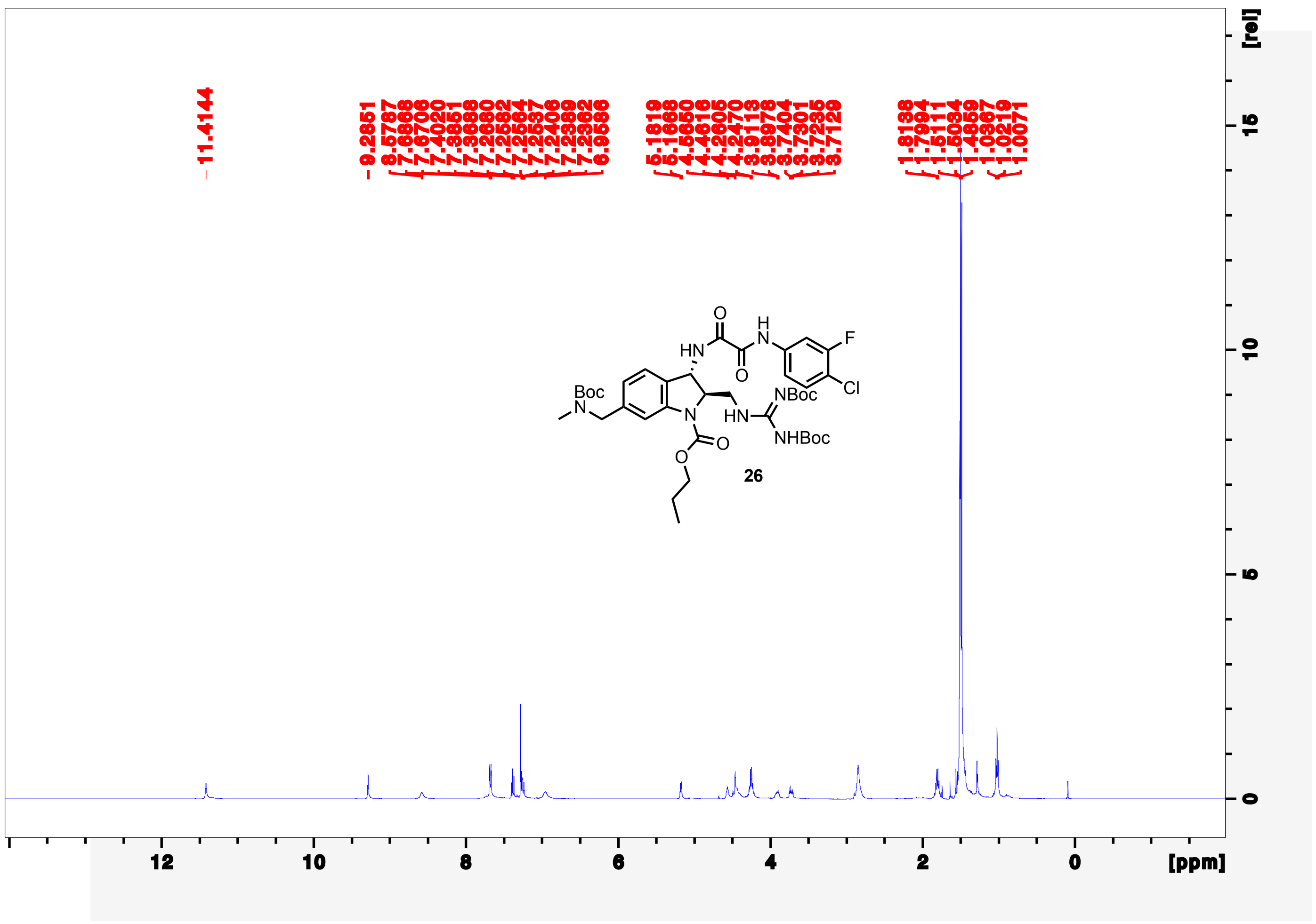

Figure A.2.26 The 500 MHz ^{1}H NMR Spectrum of compound **26** at 36.85˚C

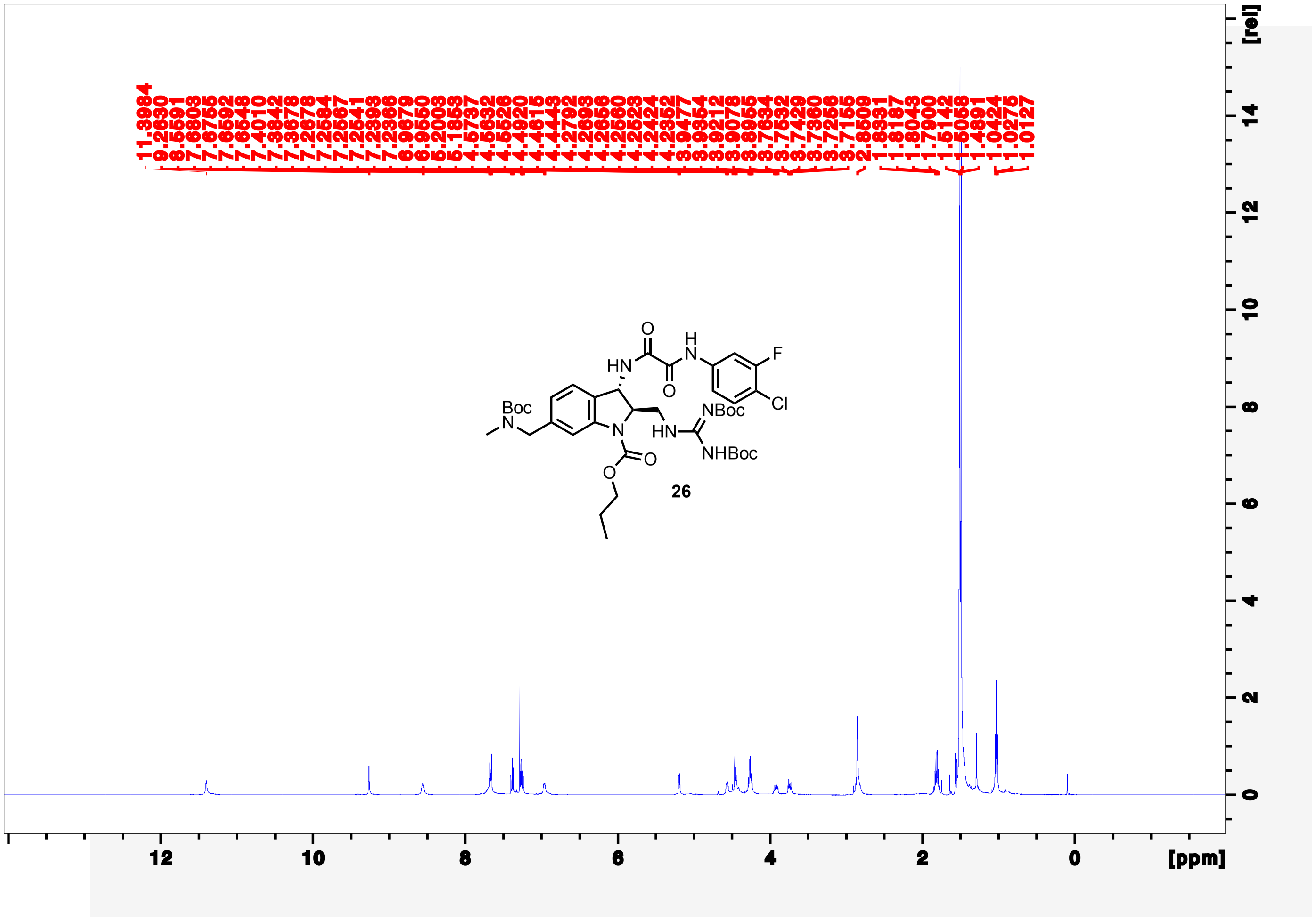

Figure A.2.27 The 500 MHz ^{1}H NMR Spectrum of compound **26** at 50˚C

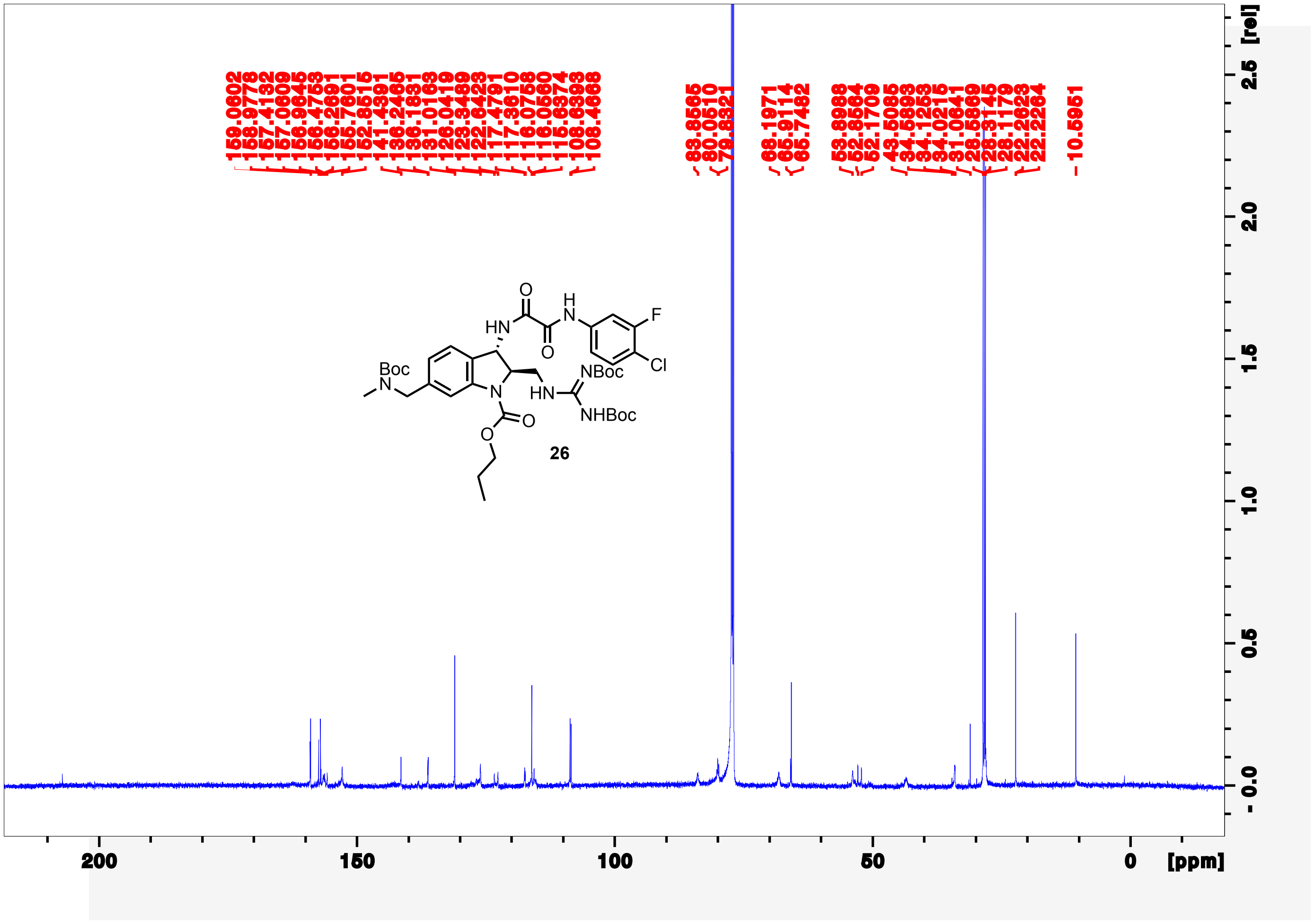

Figure A.2.28 The 125 MHz ^{13}C NMR Spectrum of compound **26** at 26.85˚C

S62

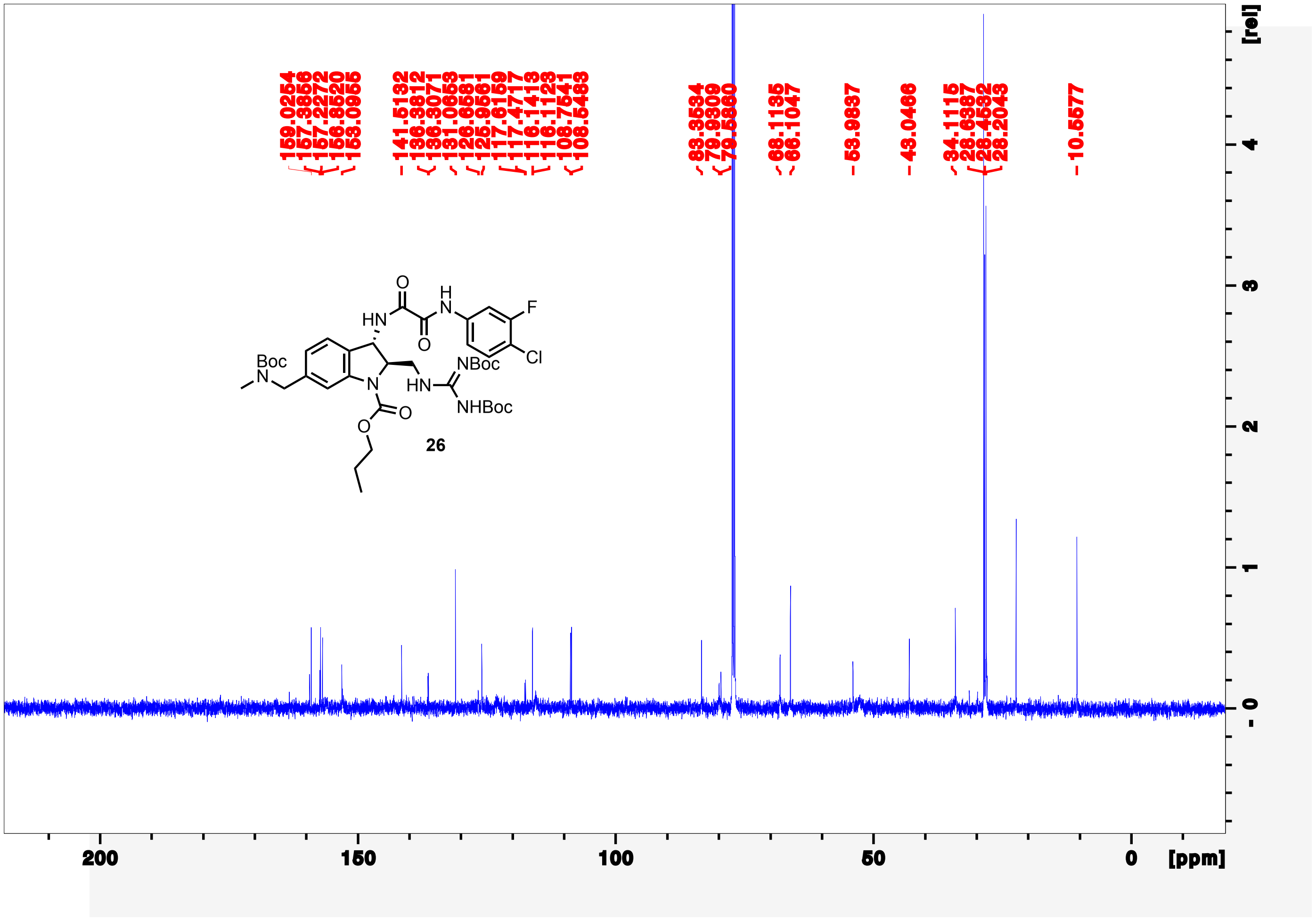

Figure A.2.29 The 125 MHz ^{13}C NMR Spectrum of compound **26** at 50˚C

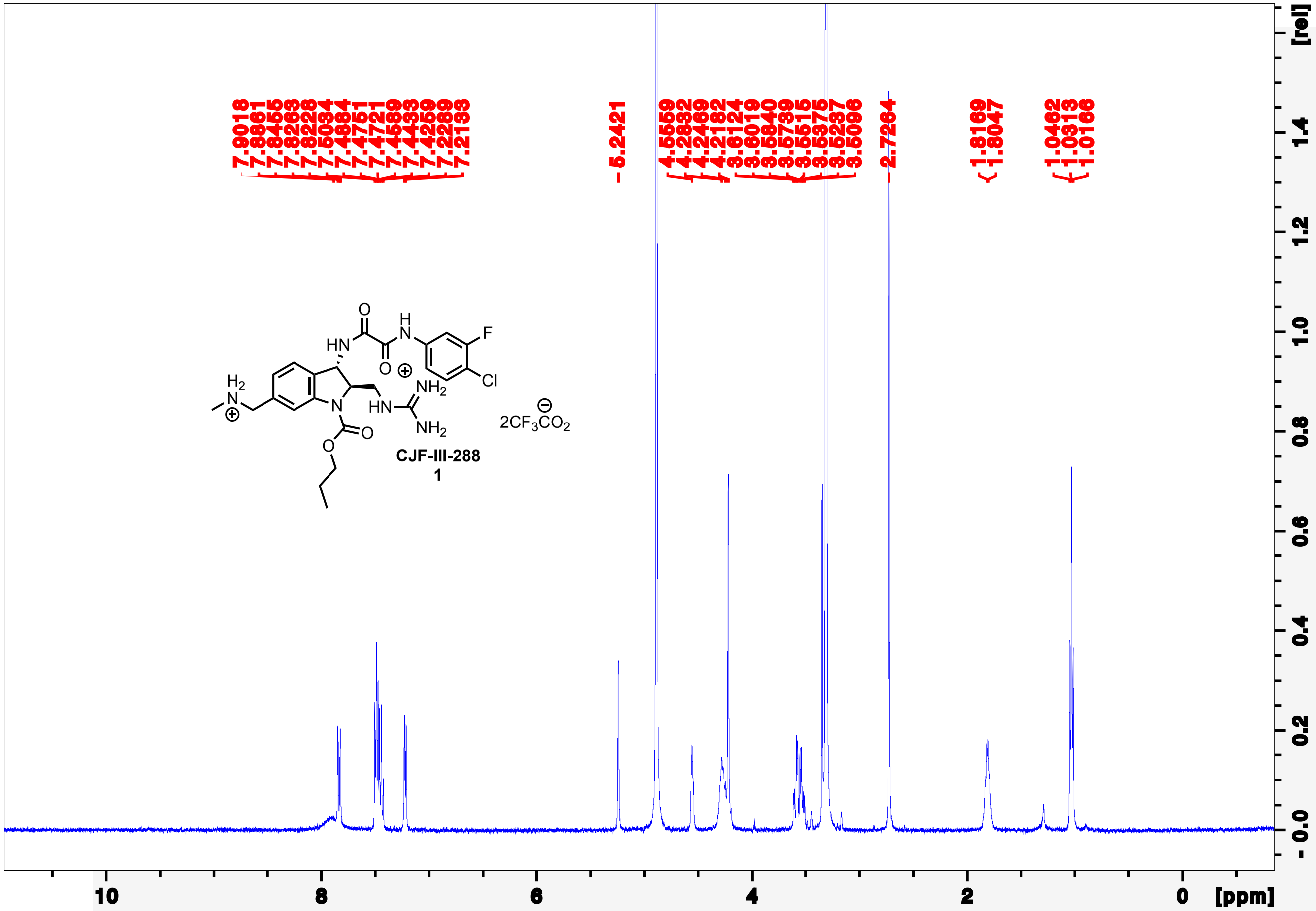

Figure A.2.30 The 500 MHz ^{1}H NMR Spectrum of compound **1**

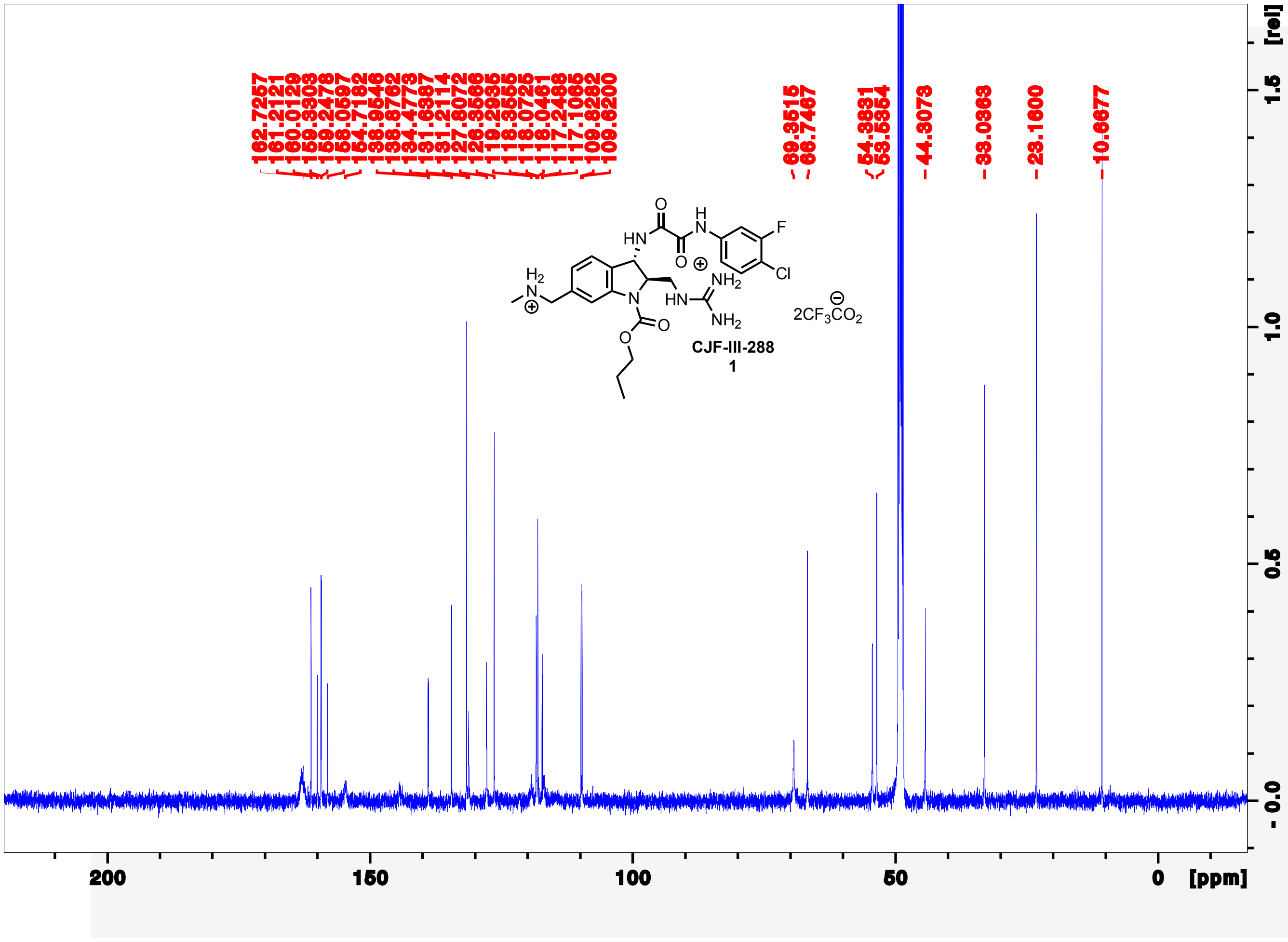

Figure A.2.31 The 500 MHz ^{13}C NMR Spectrum of compound **1**

6. X-Ray Structure Determination of Compound 15

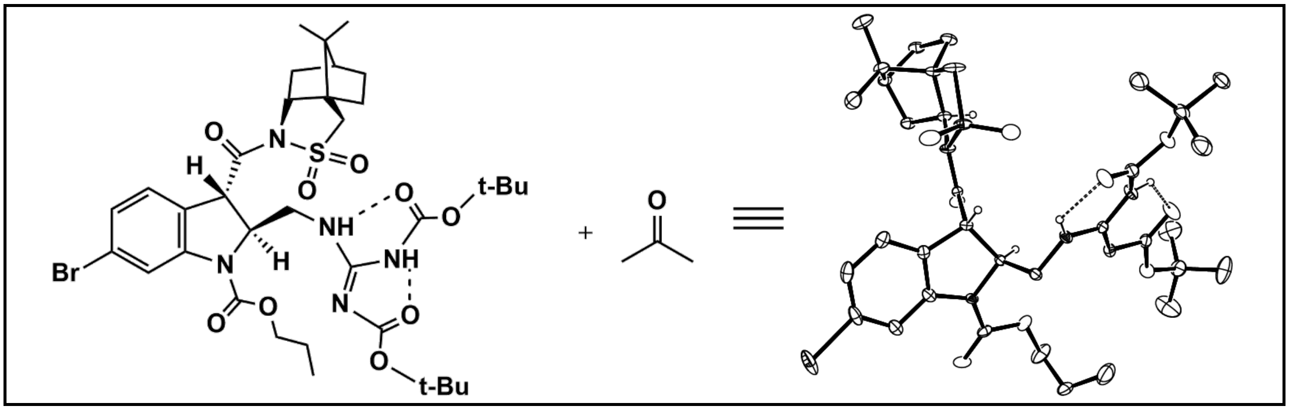

Compound **15**, $C_{38}H_{56}BrN_5O_{10}S$, crystallizes in the monoclinic space group $P2_1$ (systematic absences 0k0: k=odd) with a=12.1491(3)Å, b=12.2447(3)Å, c=14.3618(4)Å, α=90°, β=100.839(2)°, γ=90°, V=2098.38(9)Å^3, Z=2, and d_{calc}=1.353 g/cm^3. X-ray intensity data were collected on a Rigaku XtaLAB Synergy-S diffractometer [1] equipped with an HPC area detector (Dectris Pilatus3 R 200K) and employing confocal multilayer optic-monochromated Mo-Kα radiation (λ=0.71073 Å) at a temperature of 100K. Preliminary indexing was performed from a series of thirty 0.5° rotation frames with exposures of 2.5 sec. A total of 1200 frames (11 runs) were collected employing ω scans with a crystal to detector distance of 34.0 mm, rotation widths of 0.5° and exposures of 15 sec.

Rotation frames were integrated using CrysAlisPro [2], producing a listing of unaveraged F^2 and $\sigma(F^2)$ values. A total of 43167 reflections were measured over the ranges $4.036 \leq 2\theta \leq 56.564°$, $-16 \leq h \leq 14$, $-16 \leq k \leq 16$, $-19 \leq l \leq 19$ yielding 10356 unique reflections (R_{int} = 0.0362). The intensity data were corrected for Lorentz and polarization effects and for absorption using SCALE3 ABSPACK [3] (minimum and maximum transmission 0.79499, 1.00000). The structure was solved by dual methods - SHELXT [4]. Refinement was by full-matrix least squares based on F^2 using SHELXL [5]. All reflections were used during refinement. The weighting scheme used was $w=1/[\sigma^2(F_o^2)+(0.0239P)^2+1.3446P]$ where $P=(F_o^2+2F_c^2)/3$. Non-hydrogen atoms were refined anisotropically and hydrogen atoms were refined using a riding model. Refinement converged to R1=0.0332 and wR2=0.0719 for 9495 observed reflections for which $F > 4\sigma(F)$ and R1=0.0389 and wR2=0.0736 and GOF =1.053 for all 10356 unique, non-zero reflections and 516 variables. The maximum Δ/σ in the final cycle of least squares was 0.002 and the two most prominent peaks in the final difference Fourier were +0.63 and -0.56 e/Å^3.

Table 1. lists cell information, data collection parameters, and refinement data. Final positional and equivalent isotropic thermal parameters are given in Tables 2. and 3. Anisotropic thermal parameters are in Table 4. Tables 5. and 6. list bond distances and bond angles. Figure 1. is an ORTEP representation of the molecule with 50% probability thermal ellipsoids displayed.

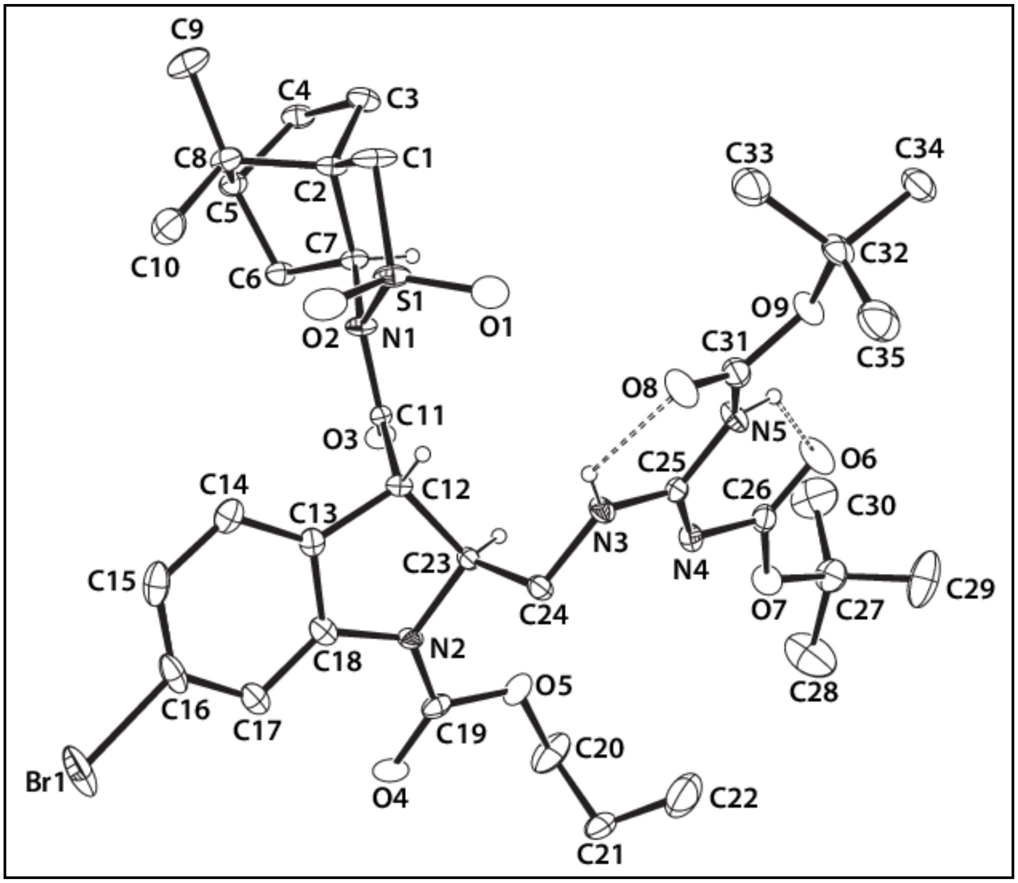

Figure A1. ORTEP drawing of the title compound with 50% thermal ellipsoids.

Table 1. Summary of Structure Determination of Compound 1490

Empirical formula	$C_{38}H_{56}BrN_5O_{10}S$
Formula weight	854.84
Diffractometer	Rigaku XtaLAB Synergy-S (Dectris Pilatus3 R 200K)
Temperature/K	100
Crystal system	monoclinic
Space group	$P2_1$
a	12.1491(3)Å
b	12.2447(3)Å
c	14.3618(4)Å
α	90°
β	100.839(2)°
γ	90°
Volume	2098.38(9)Å^3
Z	2
d_{calc}	1.353 g/cm^3
μ	1.089 mm^{-1}
F(000)	900.0
Crystal size, mm	0.28 × 0.23 × 0.07
2θ range for data collection	4.036 - 56.564°
Index ranges	-16 ≤ h ≤ 14, -16 ≤ k ≤ 16, -19 ≤ l ≤ 19
Reflections collected	43167
Independent reflections	10356[R(int) = 0.0362]
Data/restraints/parameters	10356/7/516
Goodness-of-fit on F^2	1.053

Final R indexes [I>=2σ (I)]	R_1 = 0.0332, wR_2 = 0.0719
Final R indexes [all data]	R_1 = 0.0389, wR_2 = 0.0736
Largest diff. peak/hole	0.63/-0.56 eÅ^{-3}
Flack parameter	0.008(2)

Table 2. Refined Positional Parameters for Compound 1490

Atom	*x*	*y*	*z*	U(eq)
Br1	0.81021(3)	0.75308(3)	0.73237(2)	0.03836(11)
S1	0.18375(5)	0.53966(6)	0.47077(5)	0.01694(14)
O1	0.17190(17)	0.53976(19)	0.36961(16)	0.0255(5)
O2	0.21605(18)	0.64042(17)	0.51906(17)	0.0243(5)
O3	0.42837(16)	0.34026(16)	0.51208(14)	0.0157(4)
O4	0.81096(16)	0.45808(19)	0.46613(16)	0.0231(5)
O5	0.68672(17)	0.38610(18)	0.34318(14)	0.0215(5)
O6	0.31076(18)	0.3106(2)	0.04366(17)	0.0299(5)
O7	0.49382(16)	0.2680(2)	0.09111(14)	0.0227(5)
O8	0.19760(18)	0.66138(18)	0.15929(15)	0.0225(5)
O9	0.08967(16)	0.55545(17)	0.04799(14)	0.0200(4)
N1	0.27432(18)	0.44049(19)	0.51897(17)	0.0139(5)
N2	0.62421(18)	0.4986(2)	0.44391(16)	0.0136(5)
N3	0.38700(18)	0.55801(19)	0.24241(16)	0.0146(5)
N4	0.43055(19)	0.4059(2)	0.16433(16)	0.0160(5)
N5	0.25129(19)	0.4899(2)	0.11981(17)	0.0171(5)
C1	0.0623(2)	0.4802(3)	0.5044(3)	0.0236(7)
C2	0.1019(2)	0.3830(2)	0.5676(2)	0.0148(5)
C3	0.0271(2)	0.2810(2)	0.5550(2)	0.0199(6)
C4	0.0807(2)	0.2091(2)	0.6409(2)	0.0183(6)
C5	0.1745(2)	0.2827(2)	0.6957(2)	0.0167(6)
C6	0.2708(2)	0.2847(2)	0.6396(2)	0.0167(6)
C7	0.2160(2)	0.3434(2)	0.5469(2)	0.0135(5)

C8	0.1251(2)	0.3989(2)	0.6778(2)	0.0182(6)
C9	0.0177(3)	0.4177(3)	0.7173(3)	0.0282(7)
C10	0.2060(3)	0.4909(3)	0.7166(2)	0.0289(7)
C11	0.3804(2)	0.4271(2)	0.49675(19)	0.0118(5)
C12	0.4336(2)	0.5249(2)	0.45742(19)	0.0123(5)
C13	0.5148(2)	0.5821(2)	0.53596(19)	0.0140(5)
C14	0.4924(3)	0.6451(2)	0.6102(2)	0.0189(6)
C15	0.5820(3)	0.6935(2)	0.6721(2)	0.0233(6)
C16	0.6893(3)	0.6763(2)	0.6563(2)	0.0229(7)
C17	0.7149(2)	0.6105(2)	0.5848(2)	0.0179(6)
C18	0.6242(2)	0.5638(2)	0.5244(2)	0.0150(6)
C19	0.7165(2)	0.4481(2)	0.4212(2)	0.0160(6)
C20	0.7766(3)	0.3266(3)	0.3111(3)	0.0326(8)
C21	0.8290(4)	0.3921(4)	0.2451(3)	0.0264(9)
C22	0.7488(4)	0.4232(4)	0.1549(3)	0.0459(11)
C23	0.5093(2)	0.4879(2)	0.38766(18)	0.0117(5)
C24	0.4985(2)	0.5632(2)	0.30101(19)	0.0141(5)
C25	0.3581(2)	0.4822(2)	0.17510(19)	0.0135(5)
C26	0.4021(2)	0.3280(2)	0.09450(19)	0.0168(6)
C27	0.4906(3)	0.1742(3)	0.0270(2)	0.0261(7)
C28	0.6123(3)	0.1372(4)	0.0459(4)	0.0551(13)
C29	0.4537(4)	0.2080(4)	-0.0755(3)	0.0511(11)
C30	0.4174(4)	0.0859(3)	0.0560(3)	0.0435(10)
C31	0.1805(2)	0.5783(2)	0.1138(2)	0.0174(6)
C32	0.0017(2)	0.6398(3)	0.0188(2)	0.0199(6)
C33	-0.0480(3)	0.6766(3)	0.1028(2)	0.0297(7)

C34	-0.0842(3)	0.5769(3)	-0.0516(2)	0.0253(7)
C35	0.0511(3)	0.7336(3)	-0.0286(2)	0.0323(8)
O10	0.4393(2)	0.8276(2)	0.25821(18)	0.0370(6)
C36	0.3456(2)	0.8488(2)	0.2716(2)	0.0200(6)
C37	0.2650(3)	0.9142(3)	0.2007(2)	0.0235(6)
C38	0.3048(3)	0.8101(3)	0.3577(2)	0.0212(6)
C21*	0.7627(13)	0.3071(14)	0.2233(11)	0.038(3)

Table 3. Positional Parameters for Hydrogens in Compound 1490.

Atom	*x*	*y*	*z*	U(eq)
H3	0.336667	0.606512	0.251595	0.017
H5	0.226561	0.432489	0.08527	0.021
H1a	0.024647	0.53415	0.539062	0.028
H1b	0.008606	0.456218	0.447443	0.028
H3c	0.029446	0.243827	0.494162	0.024
H3b	-0.051565	0.299575	0.557664	0.024
H4a	0.025105	0.190335	0.680616	0.022
H4b	0.11203	0.140947	0.619517	0.022
H5a	0.19828	0.263384	0.764216	0.02
H6a	0.294876	0.209852	0.626665	0.02
H6b	0.335995	0.326056	0.673799	0.02
H7	0.202951	0.289766	0.493486	0.016
H9a	-0.017138	0.486579	0.692399	0.042
H9b	0.036228	0.421307	0.786642	0.042
H9c	-0.034632	0.357377	0.698042	0.042
H10a	0.275207	0.483309	0.691428	0.043
H10b	0.223439	0.486725	0.785947	0.043
H10c	0.171181	0.561526	0.697233	0.043
H12	0.374732	0.57681	0.425769	0.015
H14	0.41759	0.65538	0.619067	0.023
H15	0.569164	0.737038	0.723714	0.028
H17	0.78998	0.598004	0.577475	0.021
H20a	0.746009	0.258488	0.279193	0.039

H20b	0.834221	0.306236	0.366718	0.039
H20c	0.846725	0.368904	0.329574	0.039
H20d	0.787084	0.256161	0.345464	0.039
H21a	0.892001	0.350111	0.228199	0.032
H21b	0.860245	0.459582	0.277784	0.032
H22a	0.719202	0.356912	0.120914	0.069
H22b	0.78858	0.466877	0.114514	0.069
H22c	0.686726	0.466046	0.170801	0.069
H22d	0.705726	0.477854	0.182772	0.069
H22e	0.709502	0.405541	0.090606	0.069
H22f	0.823175	0.452546	0.152172	0.069
H23	0.493104	0.41038	0.367633	0.014
H24a	0.514553	0.639352	0.322635	0.017
H24b	0.554646	0.541861	0.26259	0.017
H28a	0.659162	0.194748	0.026215	0.083
H28b	0.619391	0.070489	0.009827	0.083
H28c	0.636658	0.122709	0.11371	0.083
H29a	0.374338	0.228464	-0.086821	0.077
H29b	0.464352	0.146828	-0.116988	0.077
H29c	0.498535	0.270446	-0.089149	0.077
H30a	0.441265	0.07051	0.123765	0.065
H30b	0.424205	0.019521	0.019436	0.065
H30c	0.339244	0.110419	0.043707	0.065
H33a	-0.068391	0.612545	0.136709	0.045
H33b	-0.115046	0.720771	0.080298	0.045
H33c	0.007221	0.720426	0.145542	0.045

H34a	-0.050542	0.554082	-0.105393	0.038
H34b	-0.14907	0.623736	-0.074283	0.038
H34c	-0.108288	0.512243	-0.020546	0.038
H35a	0.107818	0.770951	0.018159	0.049
H35b	-0.008463	0.785206	-0.054515	0.049
H35c	0.085856	0.705303	-0.079944	0.049
H37a	0.258579	0.988084	0.2253	0.035
H37b	0.191357	0.878872	0.189561	0.035
H37c	0.292708	0.918275	0.140896	0.035
H38a	0.256725	0.745976	0.341549	0.032
H38b	0.261955	0.868405	0.38114	0.032
H38c	0.369057	0.790432	0.407058	0.032
H21c	0.694767	0.261526	0.204647	0.046
H21d	0.82748	0.264555	0.210401	0.046

Table 4. Refined Thermal Parameters (U's) for Compound 1490

Atom	U_{11}	U_{22}	U_{33}	U_{23}	U_{13}	U_{12}
Br1	0.0512(2)	0.03020(17)	0.02514(15)	-0.00292(16)	-0.01461(14)	-0.01619(18)
S1	0.0104(3)	0.0139(3)	0.0275(4)	0.0070(3)	0.0061(3)	0.0034(3)
O1	0.0195(10)	0.0299(12)	0.0264(11)	0.0105(10)	0.0024(8)	0.0047(10)
O2	0.0220(11)	0.0139(10)	0.0391(13)	0.0020(9)	0.0114(9)	0.0037(8)
O3	0.0145(9)	0.0125(9)	0.0212(10)	0.0034(8)	0.0061(8)	0.0032(8)
O4	0.0094(9)	0.0323(13)	0.0280(12)	0.0071(10)	0.0043(8)	0.0031(9)
O5	0.0205(10)	0.0283(12)	0.018(1)	0.0004(9)	0.0096(8)	0.0104(9)
O6	0.0206(11)	0.0332(13)	0.0316(12)	-0.0145(10)	-0.0065(9)	0.0066(10)
O7	0.0178(9)	0.0263(12)	0.0237(10)	-0.0085(9)	0.0035(8)	0.0048(9)
O8	0.0209(10)	0.0189(11)	0.0238(11)	-0.0049(9)	-0.0056(8)	0.0032(8)
O9	0.0158(9)	0.0214(11)	0.0199(10)	-0.0060(8)	-0.0039(8)	0.0039(8)
N1	0.0088(10)	0.0119(11)	0.0218(12)	0.0031(9)	0.0051(9)	0.0015(8)
N2	0.0082(10)	0.0183(12)	0.0138(11)	0.0023(9)	0.0009(8)	0.0007(9)
N3	0.0109(10)	0.0170(12)	0.0146(11)	-0.0015(9)	-0.0006(8)	0.0027(9)
N4	0.0169(11)	0.0174(12)	0.0128(11)	-0.0004(9)	0.0008(9)	-0.0004(9)
N5	0.0148(11)	0.0166(12)	0.0174(12)	-0.0034(10)	-0.0036(9)	0.0000(9)
C1	0.0100(12)	0.0218(16)	0.0413(19)	0.0131(14)	0.0102(12)	0.0021(11)
C2	0.0086(12)	0.0135(13)	0.0228(14)	0.0029(11)	0.0042(10)	-0.0004(10)
C3	0.0135(13)	0.0197(16)	0.0260(15)	0.0033(11)	0.0026(11)	-0.0045(11)
C4	0.0148(13)	0.0145(13)	0.0269(15)	0.0018(11)	0.0069(11)	-0.0018(11)
C5	0.0149(13)	0.0186(15)	0.0177(13)	0.0021(10)	0.0054(10)	-0.0008(10)
C6	0.0133(12)	0.0173(14)	0.0198(13)	0.0046(10)	0.0037(10)	0.0002(10)
C7	0.0113(12)	0.0117(12)	0.0185(13)	0.0009(10)	0.0052(10)	-0.0006(10)

C8	0.0162(13)	0.0164(14)	0.0242(15)	-0.0020(12)	0.0092(11)	-0.0008(11)
C9	0.0271(16)	0.0237(17)	0.0397(19)	-0.0017(14)	0.0214(15)	0.0043(13)
C10	0.0349(18)	0.0237(17)	0.0310(18)	-0.0098(14)	0.0137(14)	-0.0104(14)
C11	0.0098(12)	0.0147(13)	0.0113(12)	-0.0022(10)	0.0027(10)	-0.0009(10)
C12	0.0100(11)	0.0119(13)	0.0152(12)	0.0009(10)	0.0027(9)	-0.0002(10)
C13	0.0188(13)	0.0100(12)	0.0132(12)	0.0018(10)	0.0025(10)	-0.0024(10)
C14	0.0269(14)	0.0141(13)	0.0172(13)	0.0014(11)	0.0077(11)	0.0009(11)
C15	0.0428(18)	0.0128(14)	0.0144(14)	-0.0006(11)	0.0053(13)	0.0006(13)
C16	0.0329(17)	0.0154(14)	0.0157(14)	0.0035(11)	-0.0073(12)	-0.0102(12)
C17	0.0205(14)	0.0144(14)	0.0164(13)	0.0048(11)	-0.0028(11)	-0.0038(11)
C18	0.0162(13)	0.0112(13)	0.0158(13)	0.0047(10)	-0.0016(10)	-0.0006(10)
C19	0.0154(13)	0.0170(14)	0.0172(13)	0.0070(11)	0.0066(11)	0.0025(11)
C20	0.0358(18)	0.035(2)	0.0329(18)	0.0062(15)	0.0208(15)	0.0212(16)
C21	0.022(2)	0.036(2)	0.025(2)	-0.0066(18)	0.0138(17)	-0.0053(18)
C22	0.044(2)	0.070(3)	0.0269(19)	0.0127(19)	0.0141(17)	0.008(2)
C23	0.0101(11)	0.0124(13)	0.0121(12)	-0.0014(10)	0.0006(9)	-0.0002(10)
C24	0.0125(12)	0.0148(14)	0.0141(13)	0.0004(10)	0.0003(10)	-0.0023(10)
C25	0.0141(12)	0.0158(13)	0.0107(12)	0.0021(10)	0.0025(10)	-0.0016(10)
C26	0.0182(13)	0.0189(14)	0.0133(13)	-0.0011(11)	0.0032(10)	0.0007(11)
C27	0.0273(16)	0.0263(17)	0.0263(16)	-0.0107(13)	0.0092(13)	0.0026(13)
C28	0.032(2)	0.055(3)	0.076(3)	-0.037(2)	0.004(2)	0.0138(19)
C29	0.081(3)	0.052(3)	0.0240(19)	-0.0093(18)	0.020(2)	0.010(2)
C30	0.051(2)	0.0270(19)	0.058(3)	-0.0134(18)	0.023(2)	-0.0048(18)
C31	0.0171(13)	0.0184(14)	0.0162(13)	-0.0003(11)	0.0017(11)	-0.0004(11)
C32	0.0136(13)	0.0237(15)	0.0203(15)	-0.0006(12)	-0.0022(11)	0.0064(11)
C33	0.0228(16)	0.0363(19)	0.0290(17)	-0.0083(15)	0.0022(13)	0.0045(14)

C34	0.0164(14)	0.0332(18)	0.0237(16)	-0.0059(13)	-0.0030(12)	0.0018(12)
C35	0.0270(16)	0.036(2)	0.0312(17)	0.0098(15)	-0.0028(13)	-0.0011(14)
O10	0.0255(12)	0.0525(17)	0.0346(14)	0.0068(12)	0.0095(10)	0.0109(12)
C36	0.0219(14)	0.0151(14)	0.0224(15)	-0.0017(12)	0.0022(12)	-0.0012(11)
C37	0.0250(15)	0.0208(16)	0.0247(16)	0.0030(12)	0.0048(12)	0.0008(12)
C38	0.0258(15)	0.0184(15)	0.0190(14)	-0.0002(12)	0.0032(12)	-0.0004(12)
C21*	0.033(7)	0.043(7)	0.040(7)	-0.008(6)	0.013(6)	0.014(6)

Table 5. Bond Distances in Compound 1490, Å

Br1-C16	1.906(3)	S1-O1	1.433(2)	S1-O2	1.433(2)
S1-N1	1.696(2)	S1-C1	1.791(3)	O3-C11	1.212(3)
O4-C19	1.212(4)	O5-C19	1.345(4)	O5-C20	1.457(4)
O6-C26	1.227(4)	O7-C26	1.343(4)	O7-C27	1.469(4)
O8-C31	1.205(4)	O9-C31	1.341(3)	O9-C32	1.489(3)
N1-C7	1.478(3)	N1-C11	1.394(3)	N2-C18	1.405(4)
N2-C19	1.373(4)	N2-C23	1.482(3)	N3-C24	1.455(3)
N3-C25	1.338(4)	N4-C25	1.313(4)	N4-C26	1.380(4)
N5-C25	1.392(3)	N5-C31	1.375(4)	C1-C2	1.519(4)
C2-C3	1.536(4)	C2-C7	1.548(4)	C2-C8	1.567(4)
C3-C4	1.555(4)	C4-C5	1.547(4)	C5-C6	1.540(4)
C5-C8	1.547(4)	C6-C7	1.548(4)	C8-C9	1.535(4)
C8-C10	1.530(4)	C11-C12	1.519(4)	C12-C13	1.522(4)
C12-C23	1.550(4)	C13-C14	1.384(4)	C13-C18	1.388(4)
C14-C15	1.400(4)	C15-C16	1.381(5)	C16-C17	1.386(5)
C17-C18	1.390(4)	C20-C21	1.474(5)	C20-C21*	1.264(16)
C21-C22	1.517(6)	C22-C21*	1.719(17)	C23-C24	1.535(4)
C27-C28	1.520(5)	C27-C29	1.514(5)	C27-C30	1.507(5)
C32-C33	1.515(4)	C32-C34	1.519(4)	C32-C35	1.515(5)
O10-C36	1.217(4)	C36-C37	1.504(4)	C36-C38	1.494(4)

Table 6. Bond Angles in Compound 1490, °

O1-S1-O2	117.13(14)	O1-S1-N1	110.37(13)	O1-S1-C1	110.05(15)
O2-S1-N1	108.73(13)	O2-S1-C1	112.58(15)	N1-S1-C1	95.91(13)
C19-O5-C20	116.4(2)	C26-O7-C27	121.8(2)	C31-O9-C32	120.4(2)
C7-N1-S1	112.28(17)	C11-N1-S1	123.19(19)	C11-N1-C7	118.6(2)
C18-N2-C23	110.7(2)	C19-N2-C18	125.3(2)	C19-N2-C23	124.0(2)
C25-N3-C24	122.5(2)	C25-N4-C26	119.7(2)	C31-N5-C25	126.4(2)
C2-C1-S1	107.17(19)	C1-C2-C3	116.9(2)	C1-C2-C7	109.0(2)
C1-C2-C8	118.9(3)	C3-C2-C7	104.7(2)	C3-C2-C8	102.1(2)
C7-C2-C8	103.6(2)	C2-C3-C4	102.5(2)	C5-C4-C3	103.5(2)
C4-C5-C8	103.0(2)	C6-C5-C4	107.6(2)	C6-C5-C8	102.0(2)
C5-C6-C7	102.2(2)	N1-C7-C2	107.0(2)	N1-C7-C6	117.2(2)
C2-C7-C6	103.7(2)	C5-C8-C2	92.5(2)	C9-C8-C2	112.9(3)
C9-C8-C5	114.1(2)	C10-C8-C2	116.1(2)	C10-C8-C5	114.4(2)
C10-C8-C9	106.7(3)	O3-C11-N1	119.7(2)	O3-C11-C12	122.4(2)
N1-C11-C12	117.9(2)	C11-C12-C13	110.4(2)	C11-C12-C23	110.8(2)
C13-C12-C23	103.5(2)	C14-C13-C12	129.3(3)	C14-C13-C18	120.8(3)
C18-C13-C12	109.8(2)	C13-C14-C15	118.9(3)	C16-C15-C14	118.5(3)
C15-C16-Br1	118.8(2)	C15-C16-C17	124.1(3)	C17-C16-Br1	117.1(2)
C16-C17-C18	116.0(3)	C13-C18-N2	109.7(2)	C13-C18-C17	121.6(3)
C17-C18-N2	128.6(3)	O4-C19-O5	125.4(3)	O4-C19-N2	124.2(3)
O5-C19-N2	110.4(2)	O5-C20-C21	111.8(3)	C21*-C20-O5	116.3(7)
C20-C21-C22	113.6(3)	N2-C23-C12	103.6(2)	N2-C23-C24	108.5(2)
C24-C23-C12	112.2(2)	N3-C24-C23	111.7(2)	N3-C25-N5	117.1(2)
N4-C25-N3	119.2(2)	N4-C25-N5	123.7(3)	O6-C26-O7	123.2(3)

O6-C26-N4	128.9(3)	O7-C26-N4	108.0(2)	O7-C27-C28	102.2(3)
O7-C27-C29	111.3(3)	O7-C27-C30	109.6(3)	C29-C27-C28	110.8(3)
C30-C27-C28	110.0(3)	C30-C27-C29	112.5(3)	O8-C31-O9	126.4(3)
O8-C31-N5	126.1(3)	O9-C31-N5	107.6(2)	O9-C32-C33	110.8(2)
O9-C32-C34	101.7(2)	O9-C32-C35	109.2(2)	C33-C32-C34	110.7(3)
C35-C32-C33	112.5(3)	C35-C32-C34	111.5(3)	O10-C36-C37	121.0(3)
O10-C36-C38	121.9(3)	C38-C36-C37	117.0(3)	C20-C21*-C22	113.2(11)

This report has been created with Olex2 [6], compiled on 2022.04.07 svn.rca3783a0 for OlexSys.

References

[1] CrysAlisPro 1.171.42.72a: Rigaku Oxford Diffraction, Rigaku Corporation, Oxford, UK. (2021).
[2] CrysAlisPro 1.171.42.72a: Rigaku Oxford Diffraction, Rigaku Corporation, Oxford, UK. (2021).
[3] SCALE3 ABSPACK v1.0.7: an Oxford Diffraction program; Oxford Diffraction Ltd: Abingdon, UK, 2005.
[4] SHELXT v2018/2: Sheldrick, G.M., Acta Cryst., A, 71, 3-8 (2015).
[5] SHELXL-2018/3: Sheldrick, G.M., Acta Cryst., A, 71, 3-8 (2015).
[6] Olex2: Dolomanov,O.V., Bourhis, L.J., Gildea, R.J., Howard, J.A.K., Puschmann, H., J. Appl. Cryst., 42, 339-341 (2009).

7. References

(1) Schäfer, D.; Weiß, P.; Ermert, J.; Castillo Meleán, J.; Zarrad, F.; Neumaier, B. Preparation of No-carrier-added 6-[18f]Fluoro-l-tryptophan via Cu-mediated Radiofluorination. *European Journal of Organic Chemistry* **2016**, *2016* (27), 4621–4628.

(2) Curreli, F.; Choudhury, S.; Pyatkin, I.; Zagorodnikov, V. P.; Bulay, A. K.; Altieri, A.; Kwon, Y. D.; Kwong, P. D.; Debnath, A. K. Design, Synthesis, and Antiviral Activity of Entry Inhibitors That Target the CD4-Binding Site of HIV-1. *Journal of Medicinal Chemistry* **2012**, *55* (10), 4764–4775.

(3) Fritschi, C. J.; Anang, S.; Gong, Z.; Mohammadi, M.; Richard, J.; Bourassa, C.; Severino, K. T.; Richter, H.; Yang, D.; Chen, H.-C.; et al. Indoline CD4-Mimetic Compounds Mediate Potent and Broad HIV-1 Inhibition and Sensitization to Antibody-Dependent Cellular Cytotoxicity. *Proc. Natl. Acad. Sci.* **2023**, *120* (13).
